# Comparative study on the clinical effectiveness of low-concentration cyclosporine eye drops in patients with dry eye disease

**DOI:** 10.1371/journal.pone.0358007

**Published:** 2026-09-28

**Authors:** Jae Hyun Kim, Hui Kyung Kim, Young-Joo Shin, Chul Young Choi, Hong Kyun Kim, Eunchul Kim, Jae Yong Kim, Ji Eun Lee, SunWoong Kim, Moonwon Hwang, Jong-Seok Song, Dong Hyun Kim, Jong Soo Lee

**Affiliations:** 1 Department of Ophthalmology, Pusan National University School of Medicine, Busan, South Korea; 2 Department of Ophthalmology, Hallym University Kangnam Sacred Heart Hospital, Seoul, South Korea; 3 Department of Ophthalmology, Kangbuk Samsung Hospital, Seoul, South Korea; 4 Department of Ophthalmology, Kyungpook National University Hospital, Daegu, South Korea; 5 Department of Ophthalmology, The Catholic University of Korea, Bucheon ST. Mary’s Hospital, Bucheon, South Korea; 6 Department of Ophthalmology, Asan Medical Center, Seoul, South Korea; 7 Department of Ophthalmology, Pusan National University Yangsan Hospital, Yangsan, South Korea; 8 Department of Ophthalmology, Yonsei University Wonju Severance Christian Hospital, Wonju, South Korea; 9 Department of Ophthalmology, Inje University Busan Paik Hospital, Busan, South Korea; 10 Department of Ophthalmology, Korea University Guro Hospital, Seoul, South Korea; 11 Department of Ophthalmology, Korea University Anam Hospital, Seoul, South Korea; 12 Biomedical Research Institute, Pusan National University Hospital, Busan, South Korea; Harvard Medical School, UNITED STATES OF AMERICA

## Abstract

**Purpose:**

This study aimed to evaluate the efficacy and safety of low-concentration, nanoparticle-based cyclosporine eye drops (SCAI-001 0.01% and SCAI-001 0.02%) compared to those of the conventional Restasis^®^ (cyclosporine 0.05%) emulsion for dry eye disease (DED).

**Methods:**

In this multicenter, randomized, double-blind, Phase 2 trial, patients with DED were assigned to three groups: SCAI-001 0.01%, SCAI-001 0.02%, and Restasis. The primary efficacy endpoint was the change in tear volume after 12 weeks. Secondary efficacy endpoints included changes in tear volume at 4 and 8 weeks, corneal and conjunctival surface staining scores, tear film breakup time (TBUT), standard patient evaluation of eye dryness questionnaire score (SPEED), and ocular surface disease index (OSDI) at 4, 8, and 12 weeks.

**Results:**

Tear volume increased in all groups at 12 weeks without intergroup differences. SCAI-001 0.01% and SCAI-001 0.02% achieved earlier improvements in tear volume at 4 weeks than that of Restasis, although no significant between-group difference was observed at any time point. Conjunctival surface scores improved in all groups at 4, 8, and 12 weeks, excluding 4 weeks of Restasis. Conjunctival staining improved to a greater extent with SCAI-001 0.01% than with Restasis at weeks 4 and 8. Corneal surface scores, TBUT, SPEED, and OSDI scores improved in all groups at 4, 8, and 12 weeks without intergroup differences.

**Conclusions:**

Low-concentration cyclosporine (SCAI-001 0.01% and SCAI-001 0.02%) demonstrated efficacy and safety comparable to that of Restasis in improving DED measures.

**Trial registration:**

ClinicalTrials.gov Identifier: NCT05733624.

## Introduction

Dry eye disease (DED) is a multifactorial disease characterized by the loss of homeostasis of the tear film and ocular surface with symptoms such as ocular discomfort and blurred vision. Tear film instability and hyperosmolality cause ocular surface inflammation, leading to damage to the ocular surface [1]. It has a reported prevalence of approximately 5–50% in Asia while 5–30% in United States, Australia, and Europe [2,3]. Topical steroids are commonly used to manage inflammation of the ocular surface, but their prolonged use is restricted owing to side effects such as increased intraocular pressure, ocular infections, and cataracts [4,5].

Cyclosporine is widely recognized as an anti-inflammatory agent effective in treating DED. It achieves this by reducing the number of inflammatory cells, increasing the number of goblet cells, and increasing tear production [5]. Cyclosporine binds intracellular cyclophilin to form a complex that inhibits calcineurin phosphatase, thereby suppressing transcription of pro-inflammatory cytokines such as interleukin-2, a key driver of T-cell-mediated ocular surface inflammation in DED [6]. This immunomodulatory action reduces conjunctival lymphocyte infiltration and inflammatory cytokine expression while increasing goblet cell density [7]. It also restores tear production suppressed by inflammation through recovery of corneal nerve function which strengthens reflex arcs to stimulate tear secretion [8].

Cyclopsorine has lipophilic characteristics and low water-solubility, owing to its amino acid ring structure and strong intramolecular attraction. Regarding 0.05% micro-emulsion cyclosporine, (Restasis, Allergan Inc., Irvine, CA, USA), organic solvents are used to improve its water-solubility [9–11]. However, being an anionic emulsion, its bioavailability may be compromised when applied to the negative charged corneal surface [11]. To address this, a 0.1% cyclosporine cationic emulsion (I-Kervis, Santen SAS, Evry, France) utilizing cetalkonium chloride, a cationic surfactant, has been introduced [10,12]. Recently, 0.05% nano-emulsion cyclosporine (Cyclosporine N, Taejoon, Seoul, Korea) with reduced particle size and utilizing a self-nanoemulsifying drug delivery system has been developed and is being used in clinical practice [13].

Since the introduction of cyclosporine eye drops, studies have demonstrated improvements in tear volume, tear film breakup time, and corneal staining scores in patients with DED [11–17]. Even when compared to diquafosol, cyclosporine has shown excellent improvement in corneal staining [2,18,19]. However, the burning sensation and discomfort associated with the use of cyclosporine have been identified as factors that reduce treatment compliance. This discomfort arises because cyclosporine is almost completely insoluble in water, thus needing to be dissolved in an organic solvent and assisted by a surfactant [9,13,20]. Regarding Restasis, castor oil and polysorbate 80 are the main side effect-inducing additives [11,16,21], and in I-kervis, surfactants such as tyloxapol and poloxamer 188, were reported to induce pain on the ocular surface [22–24].

SCAI therapeutics (Daejeon, Republic of Korea) has developed a novel cyclosporine ophthalmic formulation, SCAI-001 eye drops, with lower cyclosporine concentration (0.01% and 0.02%) than conventional cyclosporine eye drop such as 0.05% cyclosporine (Restasis, Allergan Inc.) but with greater solubility and permeability only with a small amount of surfactant. To improve the poor solubility of cyclosporine, SCAI eye drops employ a proprietary technology called Molecular Associated Innovative Substance that generates nanosized molecular complexes as the dissolved raw materials pass through physically confined space. This allows for the creation of a transparent formulation and reduces the need for additives. This Phase 2 study aimed to evaluate the efficacy and safety of SCAI-001 eye drops (cyclosporine 0.01% and 0.02%) in DED treatment by comparing them with Restasis (cyclosporine 0.05%).

## Participants and methods

### Study design

This was a Phase 2, multicenter, randomized, double-blind, active-controlled trial conducted at 11 medical institutions to compare the efficacy and safety of SCAI-001 0.01% and 0.02% with Restasis 0.05% in patients with DED. Participants were prospectively recruited from 4 January 2023 to 15 May 2023 with different initiation dates but a common end date at 11 medical institutions in South Korea. Recruitment began on the following dates: Pusan National University Hospital (18 January 2023); Hallym University Kangnam Sacred Heart Hospital (11 January 2023); Kangbuk Samsung Hospital (8 March 2023); Kyungpook National University Hospital (19 January 2023); The Catholic University of Korea, Bucheon St. Mary’s Hospital (28 February 2023); Asan Medical Center (21 February 2023); Pusan National University Yangsan Hospital (20 March 2023); Yonsei University Wonju Severance Christian Hospital (13 January 2023); Inje University Busan Paik Hospital (4 January 2023); Korea University Guro Hospital (26 January 2023); Korea University Anam Hospital (9 February 2023). All recruitment ended on 15 May 2023. As it was Phase 2 trial, the sample size was not based on statistical power calculation. A target of 30 participants per group was set, and accounting for an anticipated 20% dropout rate, 38 participants per group (114 in total) were planned for enrollment. The study consisted of a 3-week screening period (visit 1 and washout period), 12-week double-blind treatment period (Visits 2–6), and a 2-week safety follow-up period. This study was approved by the Internal Institutional Review Board (IRB) and adhered to relevant ethical guidelines. All research procedures and treatments were performed in accordance with the 1964 Declaration of Helsinki and its later amendments, or equivalent ethical standards. All participants provided written informed consent (IRB number: 2209-003-129). This clinical trial was registered at ClinicalTrials.gov (Identifier: NCT05733624; https://clinicaltrials.gov/study/NCT05733624) and was first posted on February 17, 2023. The protocol was finalized before enrollment, and no changes were made to the protocol after participant enrollment began. The timing of the ClinicalTrials.gov posting did not reflect any modification of the study design.

### Study population

This study consecutively included adult men and women aged 19 years who were diagnosed to have DED and met the eligibility criteria. To be eligible, at least one of both eyes had to meet all of the following criteria at the screening: 1) Corneal staining score (Oxford grading) of ≥2 and corneal conjunctival inflammation, 2) non-anesthesia tear secretion test (Schirmer’s test) result of ≤10 mm/5 min (or nasal stimulation tear volume test result of ≥3 mm/5 min if the non-anesthesia tear volume test is 0 mm/5 min), and 3) tear film break-up time (TBUT) test result of ≤10 s, with the maximum corrected visual acuity of both eyes being 0.7 LogMAR. The visual acuity criterion was applied to exclude participants with visually significant ocular comorbidities that could confound the assessment of dry eye–related outcomes, and visual acuity was monitored throughout the study as a safety measure to detect any blurred vision associated with study medication use. Participants were required to understand the clinical trial and provide written voluntary consent. Participants registering for this clinical trial were excluded if they meet any of the criteria listed in S1 Appendix.

### Study protocol

Following the screening examination (Visit 1), registered participants underwent a discontinuation period (washout period) for at least 2 weeks from the previously administered treatment to eliminate the residual effects of prior dry eye treatments. If there is no ongoing dry eye treatment at clinical trial participation, the washout period may be omitted. At the baseline visit (Visit 2), the criteria were reevaluated, and eligible participants were randomly assigned to the following: study group 1 (SCAI-001 0.01%), study group 2 (SCAI-001 0.02%), or the control group (Restasis) in a 1:1:1 ratio. The randomization table was designed by an independent statistician using web-based statistical software (SAS, Version 9.4; SAS Institute, Cary, NC, USA) using the block randomization method with variable block size of 6 and 9. Investigators enrolling participants and those assigning interventions had no access to the sequence. Using an interactive web response system, the investigators assigned randomization numbers following the order of when the participants provided written consent. Participants, investigators, and outcome assessors were blinded to the group assignments until the database was locked. All study medications were identical in packaging to maintain blinding. Randomization codes were generated and kept by an independent statistician and were not disclosed to investigators or participants until study completion. Participants received the clinical trial medicine, one drop per eye, twice a day at 12-h intervals for 12 weeks and visited the hospital for examinations at weeks 1, 4, 8, and 12 (Visits 3–6) from the baseline visit. The visit, 12 weeks after baseline (Visit 6) marked the end of treatment for the investigational drug.

### Efficacy assessments

The primary efficacy endpoint was the change in tear volume (Schirmer’s test) at 12 weeks compared with that of the baseline. Secondary efficacies included changes in tear volume at 4 and 8 weeks and changes in corneal staining score, conjunctival staining score, TBUT, standard patient evaluation of eye dryness questionnaire (SPEED) score, and ocular surface disease index (OSDI) scores at 4, 8, and 12 weeks compared to that of the baseline. Additionally, the study and control groups (SCAI-001 0.01% vs Restasis, SCAI-001 0.02% vs Restasis) were compared for evaluation variables at each time point.

#### Schirmer’s test.

To assess tear volume, a Schirmer’s test strip was placed inside the inferior temporal conjunctival sac for 5 minutes without topical anesthesia. The length of the tear-soaked area was measured in millimeters.

#### Corneal fluorescein staining.

Corneal staining was performed after fluorescein staining under slit-lamp illumination with a cobalt blue light source using a yellow filter. The degree of corneal staining was assessed on a 6-point scale according to the Oxford grading system.

#### Conjunctival Lissamine green staining.

After lissamine green staining, a drop of preservative-free normal saline was added to the stained area. The patient then blinked 3–4 times to evenly spread the dye over the tear film. The degree of conjunctival staining was then evaluated on a 6-point scale according to the Oxford grading system under a slit-lamp microscope.

#### TBUT.

TBUT was measured by observing the eyes after fluorescein staining under slit-lamp illumination using a cobalt blue light source. Spots, lines, or absences of fluorescein in the tear layer were noted from the time of the last blink, and the time until these observations was measured in seconds. The measurement was repeated three times in succession on the same eye, with the results recorded down to 1/100 seconds.

#### SPEED.

SPEED is designed to assess symptom severity, consisting of three items of symptom duration, frequency, and severity, with total scores ranging from 0 to 28 points. It evaluates the frequency (never occurs, 0 point; sometimes, 1 point; often, 2 points; and constant, 3 points) and severity (no special problems, 0 point; tolerable, 1 point; uncomfortable, 2 points; bothersome, 3 points; and intolerable, 4 points) of the dry eye symptoms experienced by the patient.

#### OSDI.

OSDI evaluates ocular symptoms (five questions), vision-related functions (four questions), and environmental factors that may cause symptoms (three questions). Each 12 questions are rated on a 5-point scale: none of the time, 0 point; some of the time, 1 point; half of the time, 2 points; most of the time, 3 points; and all of the time, 4 points.

### Safety assessments

Ocular tolerability was assessed at 1, 4, 8, and 12 weeks of administration and extended for up to 14 weeks during the safety follow-up period. The severity of seven symptoms, including stinging/burning, itching, blurred vision, foreign body sensation, dryness, glare, and pain, was evaluated on a 4-level scale and scored (none, 0 point; mild, 1 point; moderate, 2 points; and severe, 3 points). Best-corrected visual acuity (BCVA), fundus examination, slit-lamp examination, conjunctival hyperemia, and intraocular pressure (IOP) were assessed at 1, 4, 8, 12 and 14 weeks. Abnormal findings at screening were collected as medical history, and after visit 1, clinically significant abnormal findings compared with that of the last visit were documented as adverse reactions.

### Statistical analysis

Statistical analyses were performed using SAS (version 9.4 or higher, SAS Institute, Cary, NC, USA). Efficacy analyses were performed primarily on the full analysis set, which followed the intention-to-treat principle and included all participants who received at least one dose of the study drug and had at least one post-dose measurement of tear production using Schirmer’s test. Safety analyses were conducted on the safety set, defined as all participants who received at least one dose of the study drug and underwent at least one post-dose safety assessment. Missing data in efficacy endpoints for the full analysis set were handled by the last observation carried forward method. For the per-protocol set, analyses of efficacy and safety endpoints were performed using only available data without imputation. Normality of continuous data was assessed using the Shapiro–Wilk test. Continuous variables provided information on the number of observations; mean, standard deviation, median, minimum, and maximum values. Categorical variables are presented with frequency and percentage. We compared each study group to the control groups (SCAI-001 0.01% vs Restasis, SCAI-001 0.02% vs Restasis). Baseline characteristics and safety variables were analyzed using either a two-sample t-test or the Wilcoxon rank-sum test for continuous variables, depending on the normality assumption, and Pearson’s chi-square test or Fisher’s exact test for categorical variables, as appropriate. Within-group comparisons of each post-baseline value versus baseline for the efficacy endpoints were performed using the paired t-test or Wilcoxon’s signed-rank test, depending on the normality assumption. Between-group comparisons for the efficacy endpoints at each time point (weeks 4, 8, and 12) were performed using analysis of covariance (ANCOVA), adjusting for baseline values and clinical trial site. Given the exploratory nature of this Phase 2 study, no adjustment for multiplicity to control the type I error rate was applied for these comparisons. Accordingly, all secondary endpoint findings should be interpreted as exploratory. As a post-hoc sensitivity analysis, change in tear volume was additionally analyzed using a mixed model for repeated measures (MMRM).

## Results

### Patient demography

During the 2-week screening period, 128 patients were assessed, and 116 patients were randomly assigned to three groups. One participant was excluded for not taking medication, leaving 115 participants (40 in the SCAI-001 0.01% group, 37 in the SCAI-001 0.02% group, and 38 in the control group) for the full analysis set. Of total 116 randomized participants, 108 completed the study. Among these, 96 were included in the per-protocol set after excluding participants with predefined protocol deviations or investigational product compliance of < 80% (Fig 1). Reasons for withdrawal, baseline comparisons between completers and non-completers, and a sensitivity analysis using available cases are provided in S2 Appendix. No significant baseline differences were observed between completers and non-completers, and the sensitivity analysis showed no meaningful differences from the primary analysis. Among the 116 participants, 105 were female (90.5%), with no significant differences in sex, age, height, or weight between the study and control groups (Table 1).

**Table 1:** Baseline demographic characteristics.

	SCAI-001 0.01% (N = 40)	SCAI-001 0.02% (N = 38)	Restasis (control) (N = 38)	P-value
**Sex (M:F, n[%])**	2:38 (5:95)	3:35 (7.9:92.1)	6:32 (15.8:84.2)	0.2715
**Age (years)**	45.93 ± 15.10(25–71)	44.79 ± 11.63 (24–67)	42.89 ± 12.38 (23–70)	0.6518
**Age ≥ 50 (years, n[%])**	16 (40)	11 (28.9)	13 (34.1)	0.5899
**Height (cm)**	160.84 ± 4.38 (152.7–173)	160.50 ± 6.95 (147.1–179)	162.94 ± 7.78(149.6–183.5)	0.4493
**Weight (kg)**	56.92 ± 7.73(45.2–78.5)	57.23 ± 8.68 (44.7–80)	60.56 ± 10.75 (47–87.5)	0.2785

**Fig 1:**
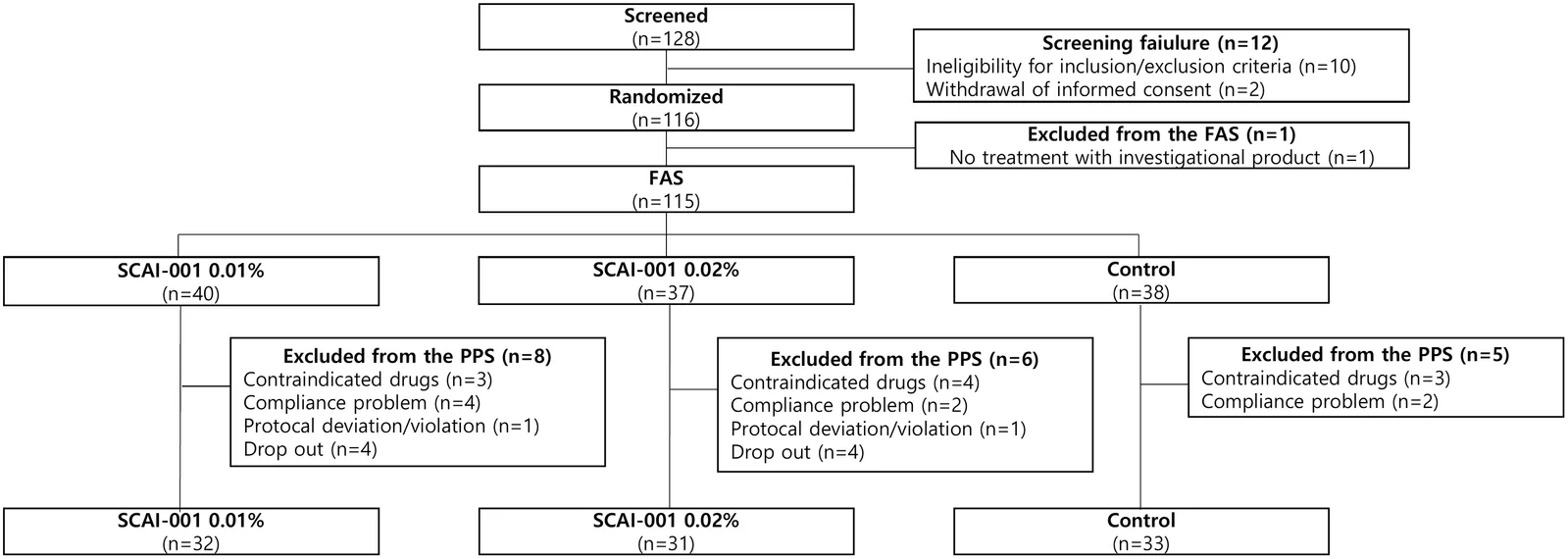
Flow diagram of study. FAS = full analysis set; PPS = per-protocol set. Reasons for exclusion from the PPS are not mutually exclusive, as some participants met more than one exclusion criterion.

### Efficacy outcomes

In both the FAS and PP, the change in tear volume at 12 weeks from baseline was significant in all groups. However, no significant differences were observed between the study and control groups. When analyzing the change in tear volume at 4 and 8 weeks, the SCAI-001 0.01% and SCAI-001 0.02% groups at 4 weeks as well as SCAI-001 0.02% and control groups at 8 weeks showed a significant increase compared with that of the baseline (Fig 2, Table 2). Sensitivity analysis using MMRM yielded results consistent with the ANCOVA findings for tear volume (S3 Appendix).

**Table 2:** Baseline values and change from baseline of tear volume by Schirmer’s test in SCAI-001 0.01%, SCAI-001 0.02%, and Restasis group at 4, 8, and 12 weeks.

	SCAI-001 0.01% (N = 40)	P-value	SCAI-001 0.02% (N = 37)	P-value	Control (Restasis) (N = 38)	P-value
**Schirmer’s test**						
Baseline	5.48 ± 2.43		5.89 ± 2.20		5.13 ± 2.35	
4 weeks	7.68 ± 6.16		8.11 ± 4.72		5.82 ± 3.62	
Change from baseline	2.20 ± 5.93	0.0081*	2.22 ± 3.77	0.0010*	0.68 ± 3.20	0.2997
8 weeks	7.30 ± 6.76		8.35 ± 4.69		7.50 ± 4.39	
Change from baseline	1.83 ± 6.54	0.1572	2.46 ± 4.00	0.0005*	2.37 ± 4.08	0.0002*
12 weeks	8.43 ± 5.86		9.81 ± 7.21		7.26 ± 4.37	
Change from baseline	2.95 ± 5.46	0.0013*	3.92 ± 6.85	0.0008*	2.13 ± 4.52	0.0075*

* Wilcoxon’s signed-rank test; Data are presented as mean ± standard deviation. P-values indicate the within-group comparison of each post-baseline value versus baseline. Baseline values and change from baseline for the secondary efficacy endpoints are presented in S4 Appendix.

**Fig 2:**
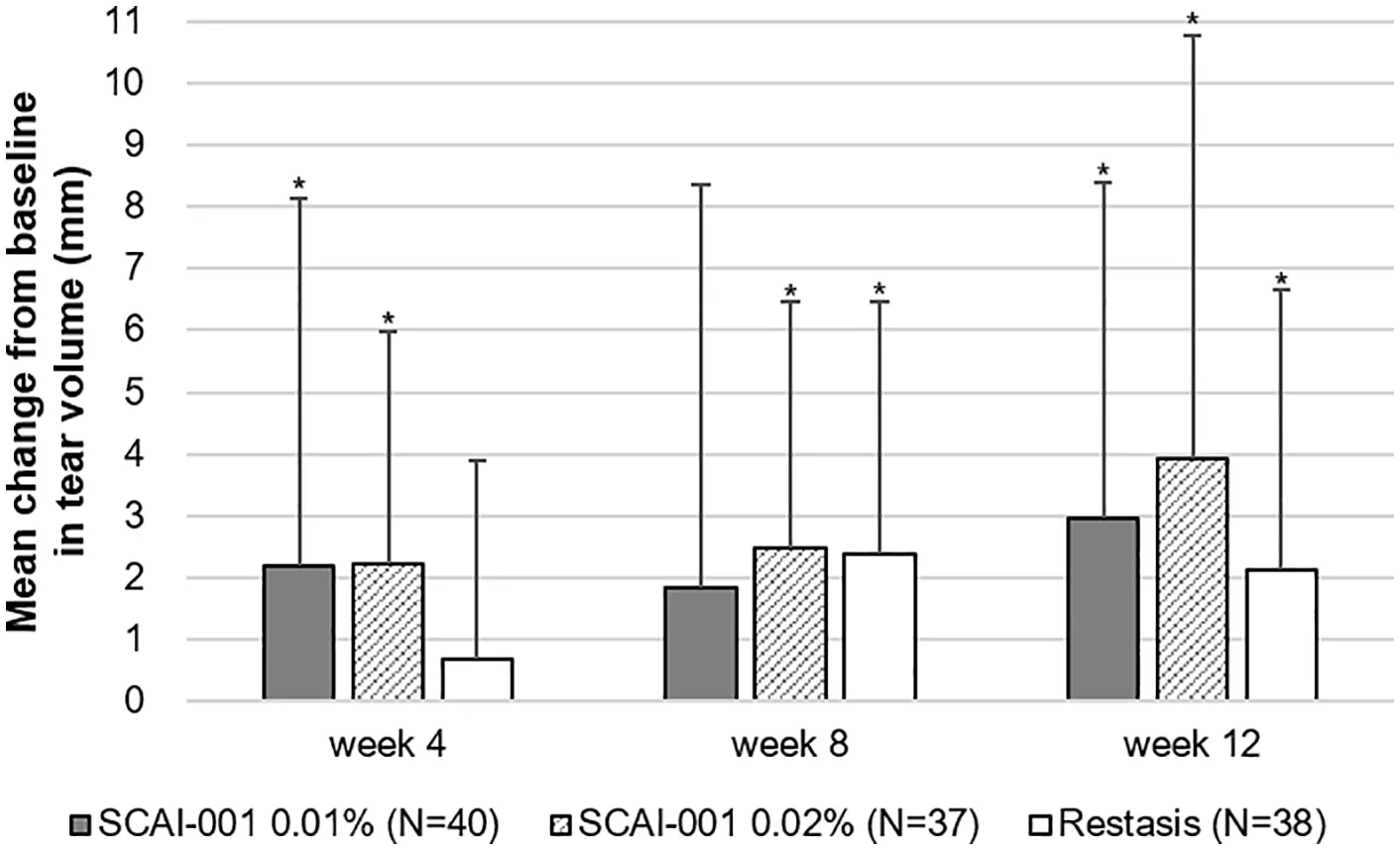
Mean change from baseline in tear volume by Schirmer’s test in SCAI-001 0.01%, SCAI-001 0.02%, and Restasis group at 4, 8, and 12 weeks. * = statistically significant difference from baseline (P < 0.05, Wilcoxon signed-rank test).

Corneal staining showed significant improvements at 4, 8, and 12 weeks compared to that of the baseline in all three groups. However, no significant differences were observed between the study and control groups (Fig 3).

**Fig 3:**
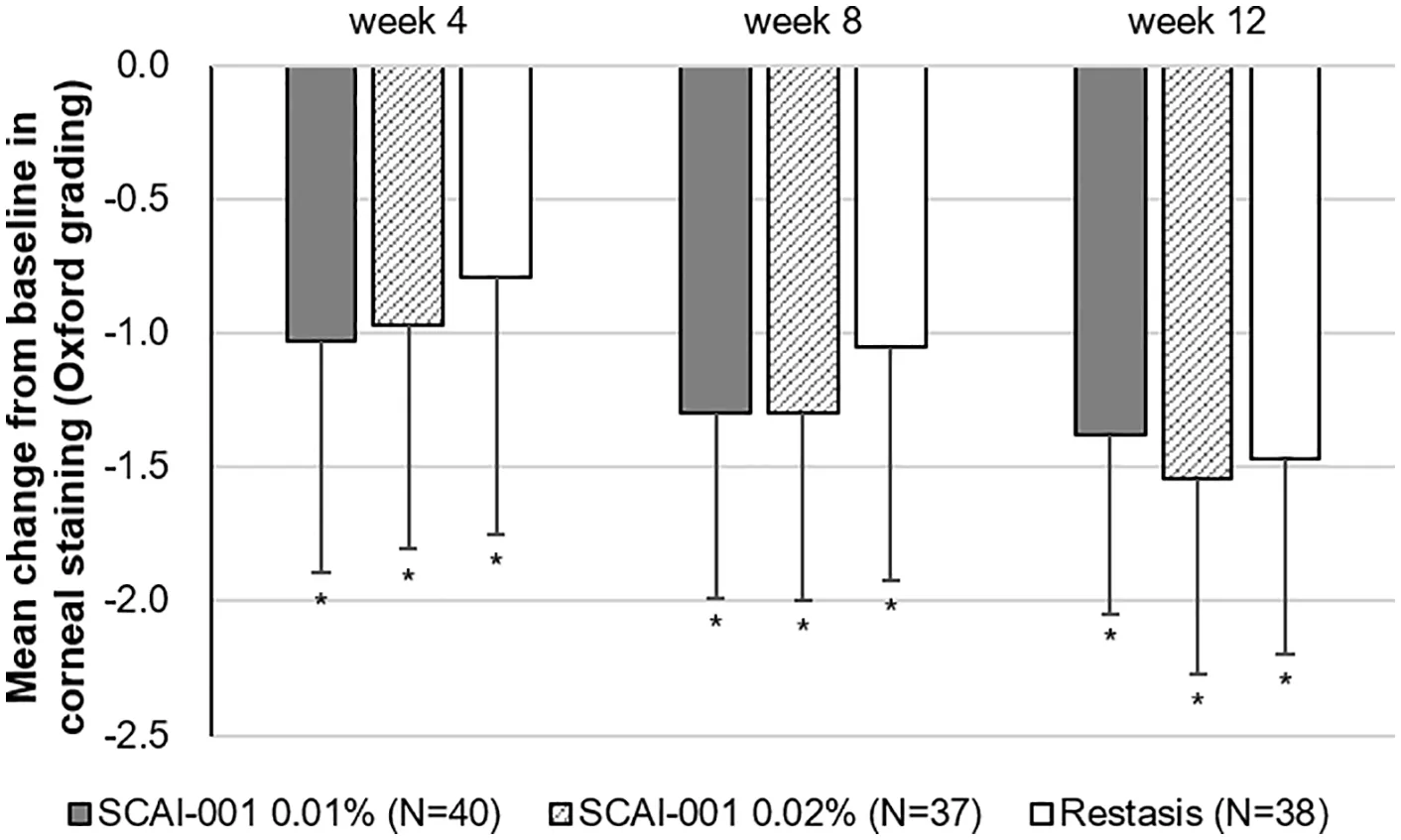
Mean change from baseline in corneal staining by Oxford grading in SCAI-001 0.01%, SCAI-001 0.02%, and Restasis group at 4, 8, and 12 weeks. * = statistically significant difference from baseline (P < 0.05, Wilcoxon signed-rank test).

After 4 weeks of treatment, the conjunctival surface staining score showed significant improvement in both SCAI-001 0.01% and SCAI-001 0.02%, whereas Restasis did not demonstrate such improvement. At 8 and 12 weeks, all three groups showed significant improvements. When comparing the study and control groups, the change in the conjunctival staining score at 4 and 8 weeks in the SCAI-001 0.01% group was statistically significant compared to that of the control group (Fig 4).

**Fig 4:**
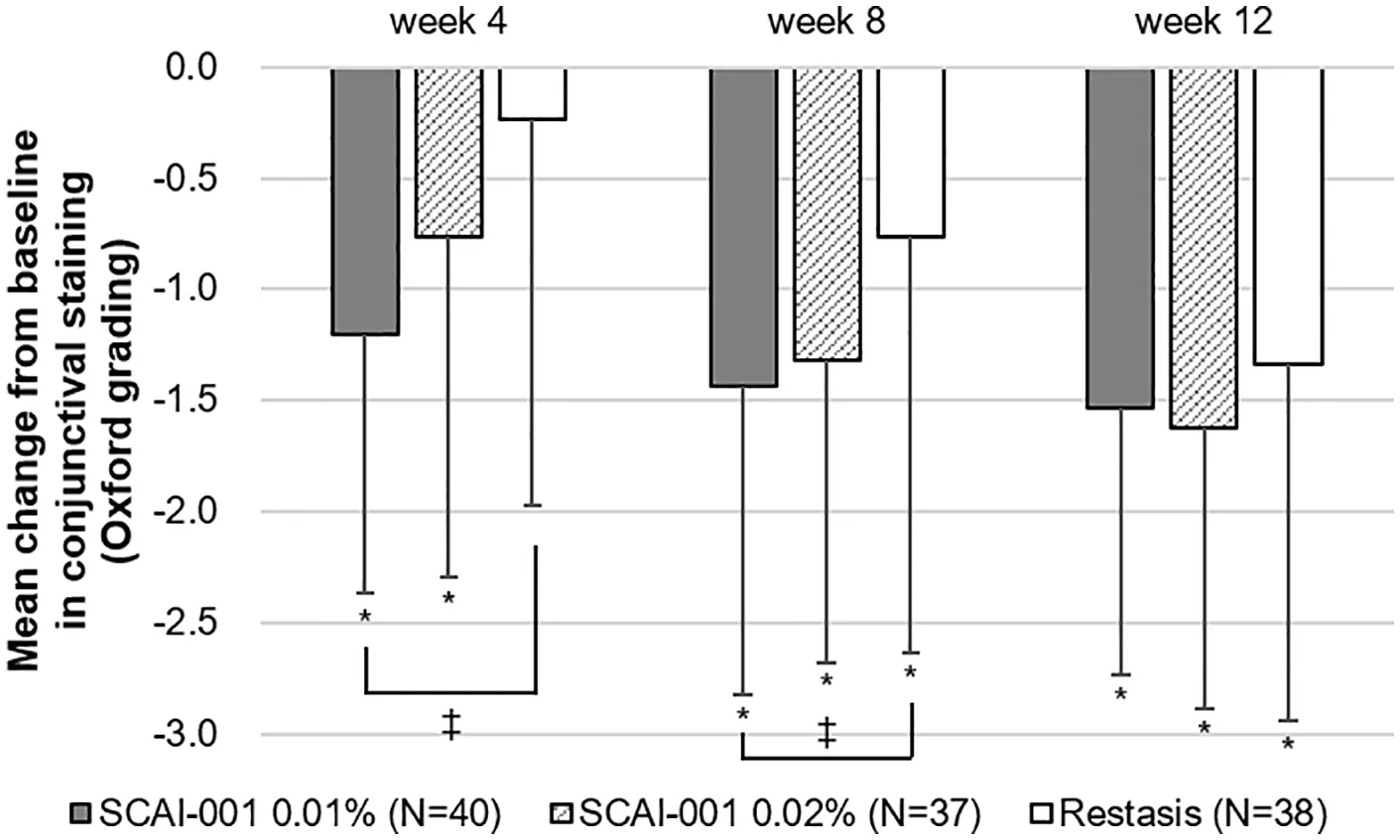
Mean change from baseline in conjunctival staining by Oxford grading in SCAI-001 0.01%, SCAI-001 0.02%, and Restasis group at 4, 8, and 12 weeks. * = statistically significant difference from baseline (P < 0.05, Wilcoxon signed-rank test). ‡ = statistically significant difference between groups (P < 0.05, ANCOVA).

Other secondary efficacy measures, including TBUT, SPEED, and OSDI, showed significant increases at 4, 8, and 12 weeks in all three groups compared to the baseline. However, there was no intergroup difference between the groups in each follow up period (Fig 5, Fig 6, Fig 7).

**Fig 5:**
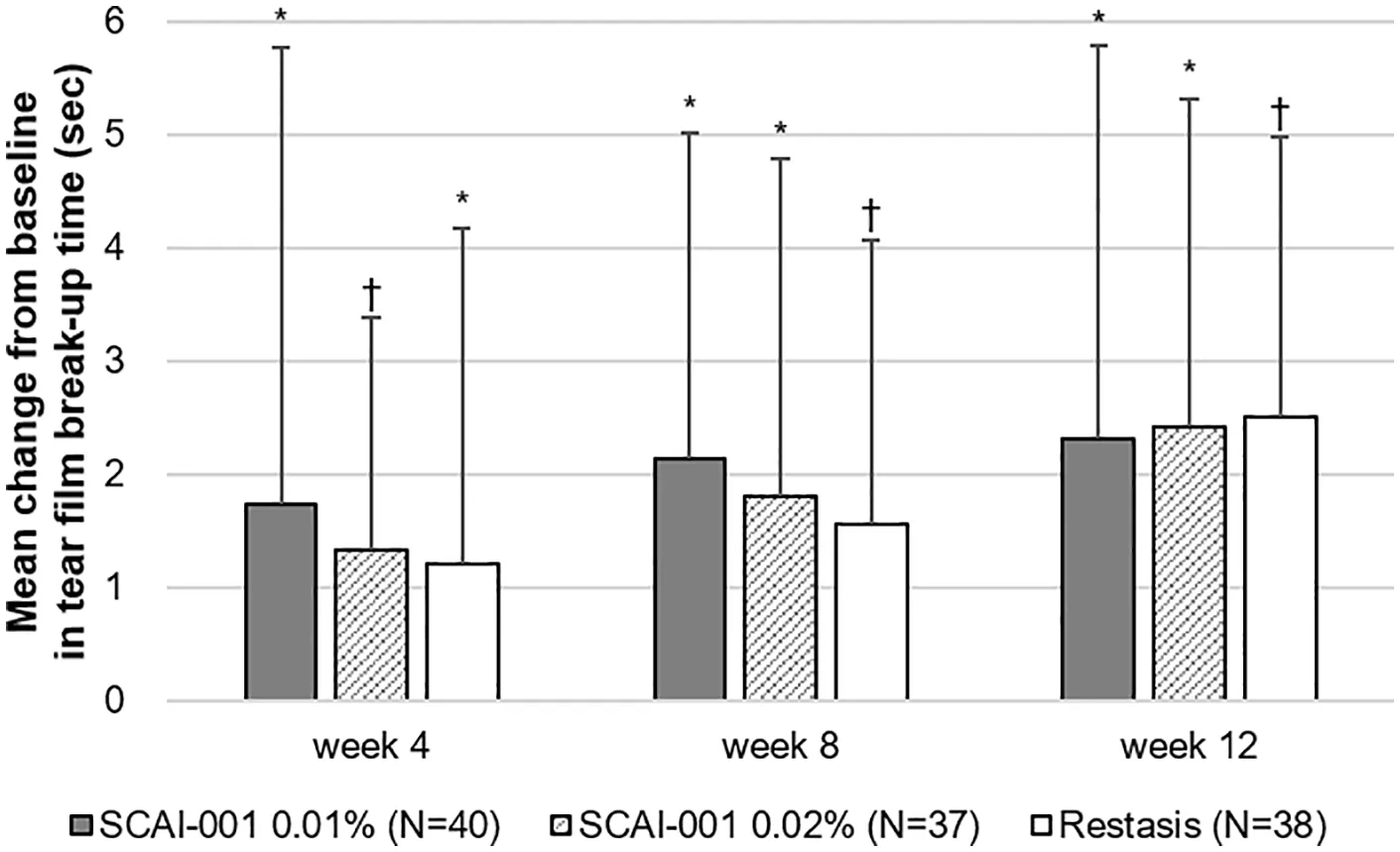
Mean change from baseline in tear film break-up time in SCAI-001 0.01%, SCAI-001 0.02%, and Restasis group at 4, 8, and 12 weeks. * = statistically significant difference from baseline (P < 0.05, Wilcoxon signed-rank test). † = statistically significant difference from baseline (P < 0.05, paired t-test).

**Fig 6:**
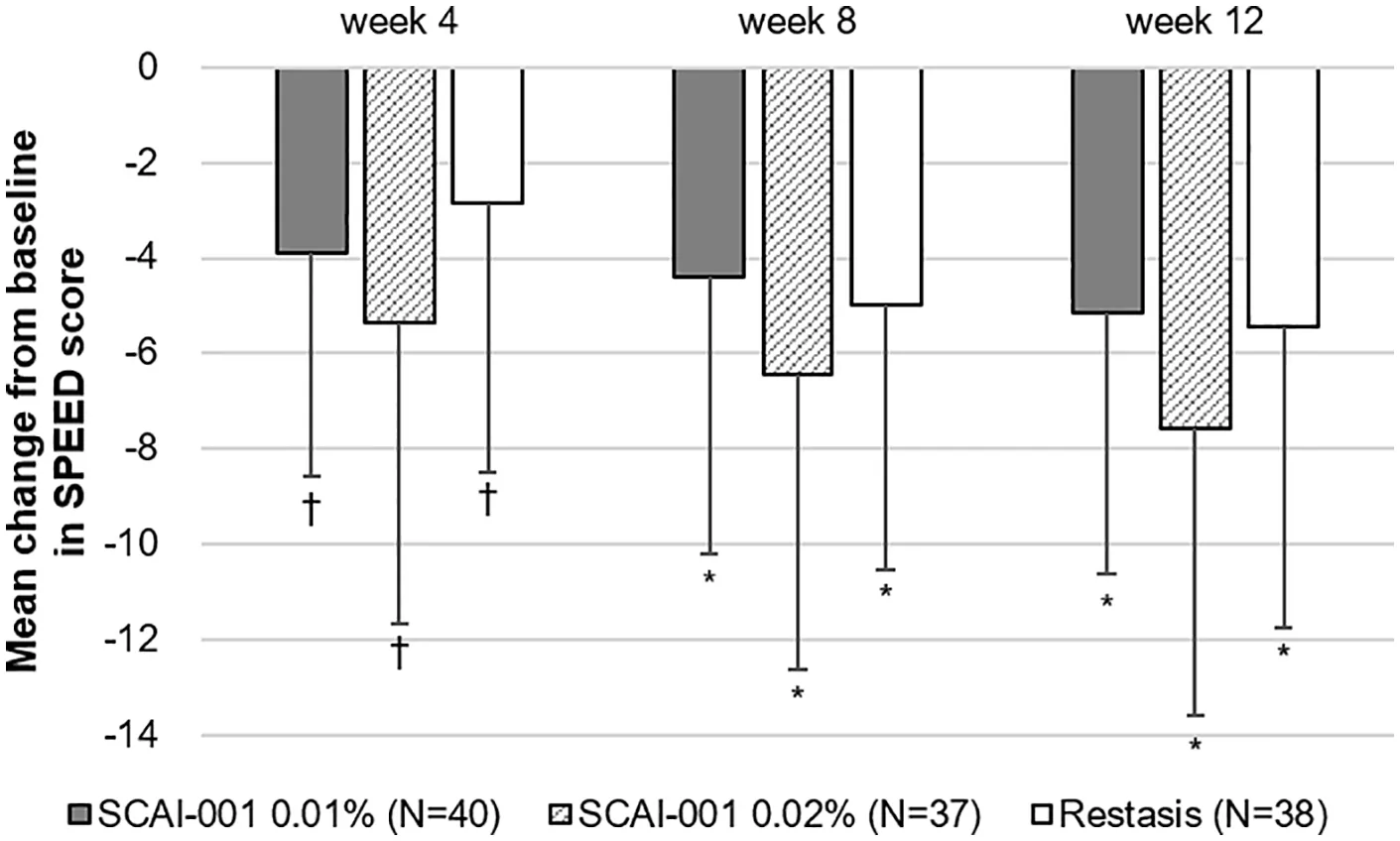
Mean change from baseline in SPEED score in SCAI-001 0.01%, SCAI-001 0.02%, and Restasis group at 4, 8, and 12 weeks. * = statistically significant difference from baseline (P < 0.05, Wilcoxon signed-rank test). † = statistically significant difference from baseline (P < 0.05, paired t-test).

**Fig 7:**
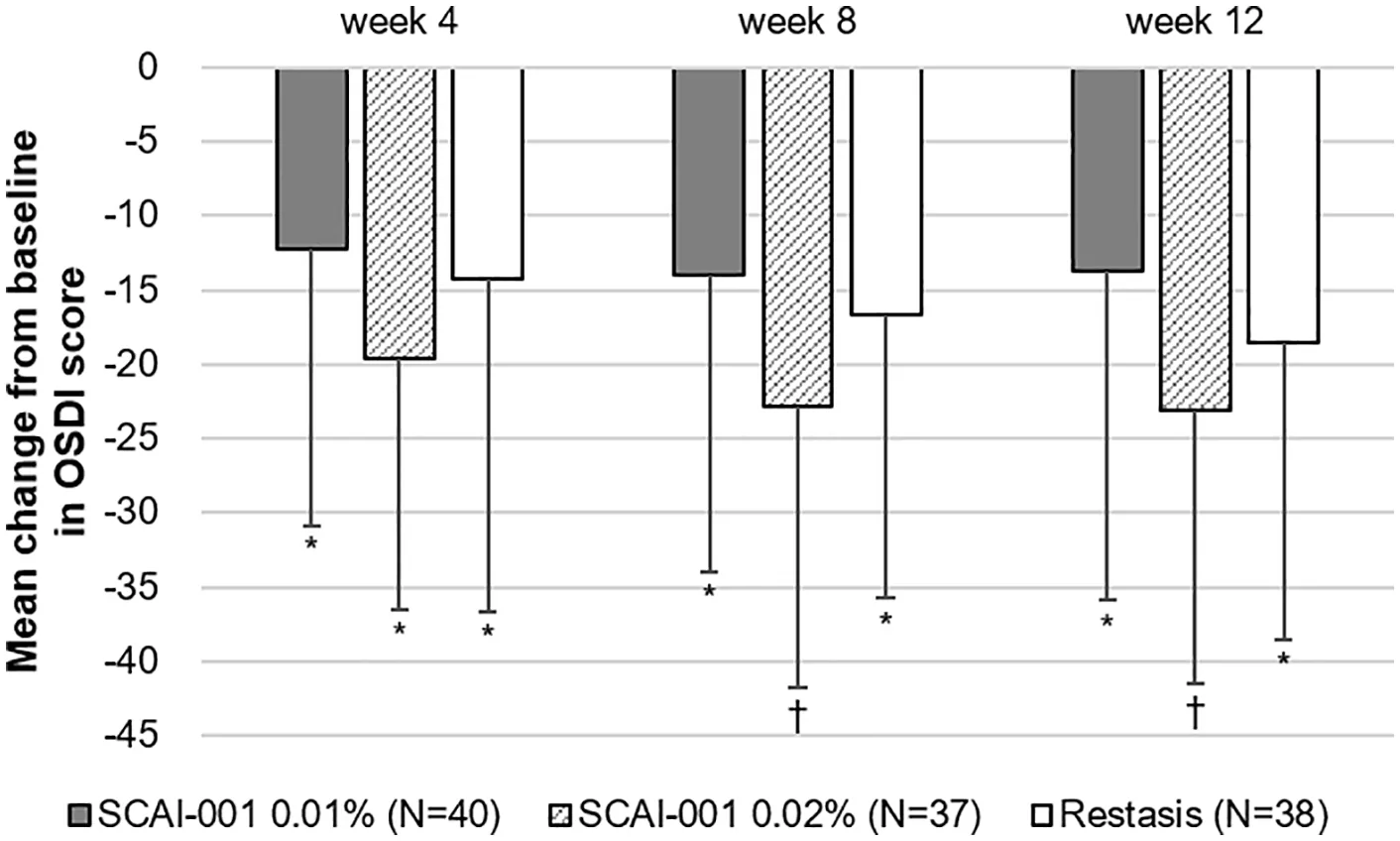
Mean change from baseline in OSDI score in SCAI-001 0.01%, SCAI-001 0.02%, and Restasis group at 4, 8, and 12 weeks. * = statistically significant difference from baseline (P < 0.05, Wilcoxon signed-rank test). † = statistically significant difference from baseline (P < 0.05, paired t-test).

### Safety outcomes

In the ocular tolerability evaluation, no differences were observed in the severity scores of the seven symptoms between the study and control groups (Fig 8). Additionally, no significant between-group differences were observed in BCVA, fundus examination, slit-lamp examination, or IOP at any study visit.

**Fig 8:**
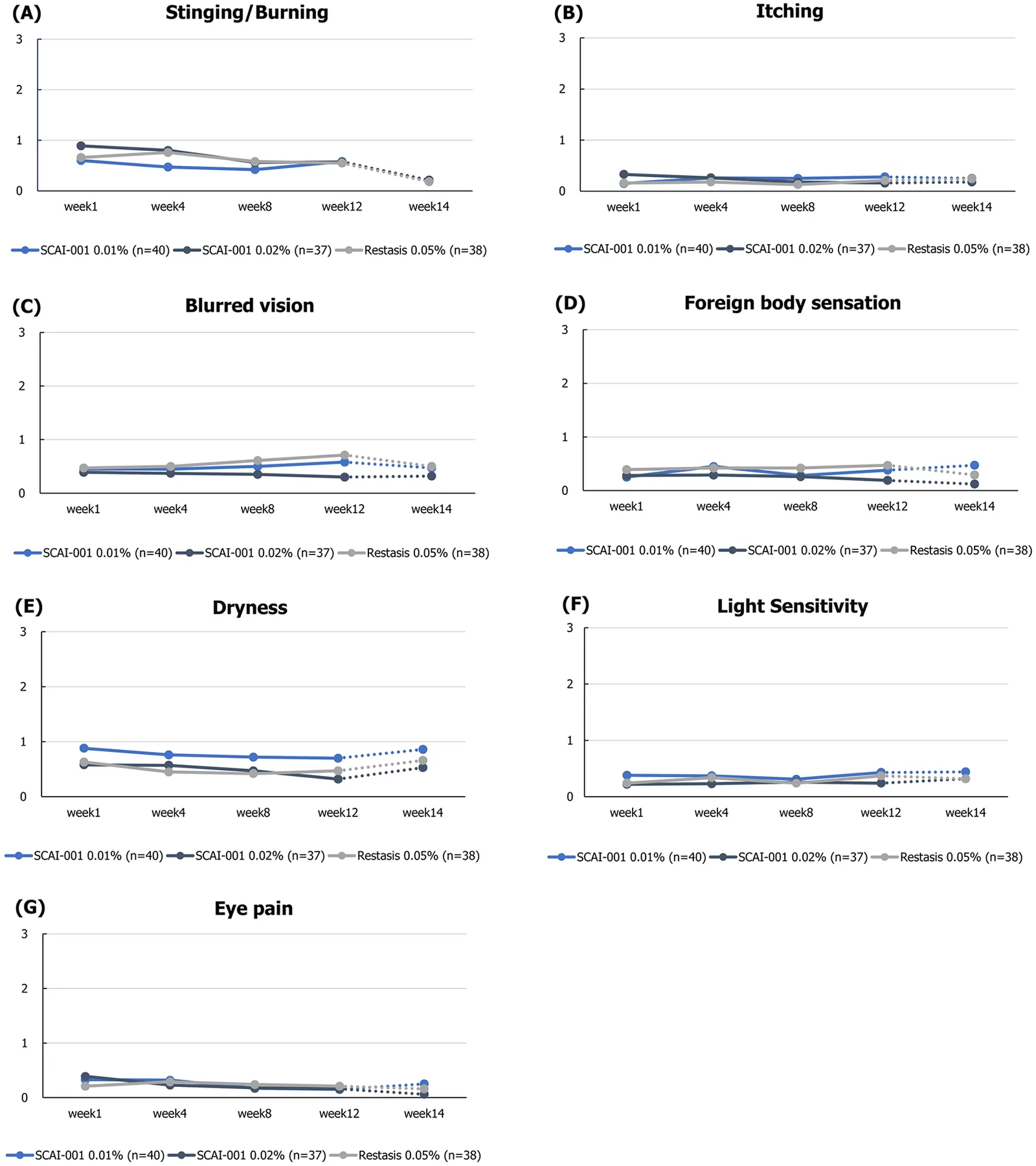
Change in ocular tolerability symptom from baseline with intergroup comparison. No significant difference was observed in the symptom scores at each visit between the SCAI-001 and Restasis groups. (all P > 0.05; Wilcoxon rank-sum test).

## Discussions

This study utilized Molecular Associated Innovative Substance technology to enhance the solubility of cyclosporine using a minimal amount of surfactant. This approach yielded SCAI-001 eye drops with improved solubility and ocular penetration. In the present study, both SCAI-001 concentrations produced significant within-group improvements from baseline in tear volume by Schirmer’s test, corneal and conjunctival staining scores, TBUT, SPEED, and OSDI scores throughout the 12-week treatment period, with an ocular tolerability profile comparable to that of Restasis. Although SCAI-001 did not show statistically significant between-group superiority over Restasis on these measures, these findings suggest that a substantially reduced cyclosporine concentration can still achieve efficacy and safety comparable to the standard 0.05% formulation, enabled by enhanced drug solubility and ocular penetration.

Primary efficacy exhibited significant improvements in all three groups, indicating that, by reducing inflammation with cyclosporine, the integrity of the corneal epithelium increases, leading to enhanced stimulation of sensory nerve endings in the cornea and conjunctiva, ultimately normalizing nerve transmission signals to the lacrimal gland [11,25]. Consequently, both quantitative and qualitative improvements in lacrimal gland-produced tears were observed, suggested by a more favorable Schirmer’s test outcome.

Regarding the secondary efficacies of tear volume changes at 4 and 8 weeks, both the SCAI-001 0.01% and SCAI-001 0.02% groups showed significant increases at 4 weeks, whereas the SCAI-001 0.02% and Restasis groups displayed significant changes at 8 weeks. Compared to Restasis, the increase in tear secretion observed at the initial 4-week period following the use of SCAI eye drops may stems from the formation of more stable nanosized uniform molecules, which are expected to enhance its corneal solubility [9,13,16,26]. In contrast, Restasis may face challenges due to its non-uniform particle size, ranging from 67 to 2,489 nm, leading to potential instability and lower corneal permeability [14,27,28]. However, the comparison between the study and control groups did not yield significant differences in tear volume changes at each time point (Fig 2). Further clinical studies are needed to clarify how particle size uniformity contributes to this exploratory earlier clinical outcome in tear volume.

SCAI-001 0.01%, and SCAI-001 0.02% eye drops exhibited significant changes in dry eye indicators, including corneal staining, conjunctival staining, and TBUT at 4, 8, and 12 weeks. Corneal staining, conjunctival staining, and TBUT depend on mucus deficiency, meibomian gland dysfunction, and corneal epithelium stability [29]. Han et al. reported that the higher the cyclosporine concentration, the greater the effect [12]. However, in this study, even at low concentrations of SCAI-001 (0.01% and 0.02%), improvement in dry eye indicators was observed to be similar to Restasis. In particular, the improvement in the conjunctival staining scores at 4 and 8 weeks in the SCAI-001 0.01% group was significantly greater than that in the Restasis group (Fig 4). This may be partly explained by the lower surfactant content of SCAI-001 relative to Restasis, given that ophthalmic surfactants such as polysorbate 80 have been shown to exert dose-dependent cytotoxic and irritant effects on the conjunctival eye assays [30]. Similarly, CyclASol, a water-free cyclosporine 0.1% formulation that eliminates the need for solubilizing surfactants altogether, demonstrated significantly greater improvement in conjunctival staining compared with Restasis [31].

The overall results are consistent with a growing body of evidence on nanotechnology-based cyclosporine formulations. Rao et al. demonstrated that a micellar nanoparticulate cyclosporine 0.05% emulsion achieved efficacy exceeding conventional Restasis on Schirmer’s test, ocular surface staining, and OSDI scores in a multicenter, active-controlled trial [32]. Similarly, Eom et al. reported that a 0.08% nanoemulsion cyclosporine formulation administered once daily achieved noninferior improvements in OSDI, TBUT, ocular surface staining, and Schirmer’s test compared with conventional 0.05% cyclosporine administered twice daily [33]. A recent meta-analysis of seven randomized trials likewise found that nanoemulsion-based cyclosporine formulations produced significant improvements in TBUT and Schirmer’s test relative to conventional formulations [34]. Taken together with the present results, these data support the premise that nanosizing strategies can preserve therapeutic efficacy while allowing a reduction in concentration or dosing frequency.

In chronic diseases that require continuous use of eye drops over a long period, patient compliance with medication is very important for successful treatment. Non-compliance in patients with DED can lead to blurred vision and social concerns such as reduced work productivity and reduced quality of life [10]. According to various reports, patients using topical anti-inflammatory eye drops such as 0.05% cyclosporine or lifitegrast have been reported to experience discomfort, itching, or burning sensation during instillation, leading to non-compliance or medication discontinuation in 2.5–60% of cases [25,35].

In this study, discomfort was expected to improve with SCAI-001 eye drops, as it reduces the surfactant concentration. However, no significant difference was observed in the intraocular tolerance between the SCAI-001 and Restasis groups (Fig 8). This was a Phase 2 clinical trial, which is primarily designed to generate preliminary evidence on dose-response relationships and efficacy signals to inform subsequent Phase 3 development, rather than to provide confirmatory comparisons of safety and tolerability outcomes. Accordingly, the sample size was determined based on the primary efficacy endpoint (change in Schirmer’s test score at 12 weeks), and the study was not specifically powered to detect between-group differences in ocular tolerability. Therefore, the absence of statistically significant differences in symptom severity scores may not be interpreted as definitive evidence of equivalent irritation profiles. Adequately powered Phase 3 trials with tolerability-specific endpoints will be necessary to confirm whether the reduced excipient content of SCAI-001 confers a meaningful advantage in ocular comfort.

Even though the mechanism whereby irritation occurs when instilling cyclosporine is poorly understood, it is known that the surfactant used to create a mixed form of cyclosporine emulsion stimulates the transient receptor potential vanilloid type 1 (TRPV1), also known as the capsaicin receptor. This stimulation induces ocular surface irritation [24]. According to studies on cyclosporine in young female patients, factors with higher ocular surface staining scores, higher expression levels of matrix metalloproteinase 9 in tears, lower tear secretion, and shorter therapeutic durations of eye drops exhibit poor compliance and complaints of discomfort [10,18]. Chronic inflammation on the ocular surface sensitizes the corneal peripheral sensory nerves inducing neuroplastic changes, making TRPV1 more easily, spontaneously, or exaggerated [36,37]. This suggests that compliance may be reduced in patients with relatively severe inflammation associated with DED. In future studies, it will be necessary to evaluate ocular tolerability and compliance with low-dose cyclosporine based on the degree of dry eye and inflammation.

A limitation of this study is that tear volume by Schirmer’s test, the primary efficacy endpoint, is known to have high measurement variability due to reflex tearing during the test procedure. While this endpoint was chosen based on regulatory precedent and consistency with the reference drug’s pivotal trials, as well as its relevance to the mechanism of action of cyclosporine, future studies may benefit from adopting co-primary sign-and-symptom endpoints, such as corneal staining combined with a validated symptom score, as increasingly recommended by regulatory guidance for dry eye disease trials [38,39].

This Phase 2 study investigated the clinical efficacy and safety of low concentrations of cyclosporine for treating dry eye symptoms. Both the SCAI-001 0.01% and SCAI-001 0.02% formulations showed improvements in objective measures such as tear secretion, corneal and conjunctival surface staining scores, and TBUT over 12 weeks, as well as subjective measures such as SPEED and OSDI, comparable to that of Restasis. They also showed earlier increases in tear volume and better conjunctival improvement compared to Restasis but these exploratory findings were not confirmed by significant between-group differences and warrant further investigation in a larger Phase 3 trial. In conclusion, low-concentration cyclosporine eye drops should be further developed as an effective treatment for DED.

## Supporting information

S1 Appendix Exclusion criteria for the randomized controlled trial. (DOCX)

S2 Appendix Reason for withdrawal by treatment group, comparison of baseline characteristics between study completers and non-completers, and sensitivity analysis of the primary efficacy endpoint using a mixed-effects model for repeated measures. (DOCX)

S3 Appendix Sensitivity analysis of change in tear volume by Schirmer’s test from baseline using a mixed model for repeated measures. (DOCX)

S4 Appendix Baseline values and change from baseline for secondary efficacy endpoints by visit and treatment group. (DOCX)

S1 Checklist CONSORT checklist. (DOCX)

S1 Fig CONSORT flow diagram of study participants. (DOCX)

S1 Protocol Study protocol. (PDF)

S1 Dataset Study dataset. (ZIP)
