# Supplementary material for: Comparative study on the clinical effectiveness of low-concentration cyclosporine eye drops in patients with dry eye disease
Source: PLoS One. 2026 Sep 28;21(9):e0358007. doi: 10.1371/journal.pone.0358007 (PMC13618915; doi:10.1371/journal.pone.0358007)
Supplement: Supplementary file 1 [file pone.0358007.s007.pdf]

Protocol

A Multicenter, Active control, Parallel group, Randomized, Double-blind, Phase II Clinical Trial to Evaluate the Efficacy and Safety of SCAI-001 Eye Drops in Patients with Dry Eye Disease

**Protocol No.**	SCAI-001-201
**Version No. (version date)**	2.3 (20Feb2023)
**Investigational Product**	Test Drug 1: SCAI-001 Eye Drops 0.01%<br>Test Drug 2: SCAI-001 Eye Drops 0.02%<br>Comparator: Restasis® 0.05%
**Target Disease**	Dry eye disease
**Phase of Development**	Phase 2
**Sponsor**	SCAI Therapeutics
**Coordinating Investigator**	Professor Jong Soo Lee, Pusan National University Hospital

[Protocol Agreement]

Title: A Multicenter, Active control, Parallel group, Randomized, Double-blind, Phase II Clinical Trial to Evaluate the Efficacy and Safety of SCAI-001 Eye Drops in Patients with Dry Eye Disease

Protocol Identifier: SCAI-001-201 Version (date): 2.3 (20Feb2023)

I have read and reviewed this clinical trial protocol, and understand and agree that it contains all information required for the conduct of the study. I will make reasonable efforts to ensure that the study is completed as scheduled. I will conduct the study in compliance with the Declaration of Helsinki, ICH-Good Clinical Practice, and all applicable local regulations.

Principal Investigator

Printed Name ______________________________

Signature ______________________________

Date (DD/MMM/YYYY) ______________________________

Sponsor

Printed Name ______________________________

Signature ______________________________

Date (DD/MMM/YYYY) ______________________________

[Title of Contents]

[PROTOCOL AGREEMENT] 2

[TITLE OF CONTENTS] 3

[GLOSSARY OF TERMS AND ABBREVIATIONS] 11

1. TITLE AND PHASE OF STUDY 12
1.1. Title of Study 12
1.2. Phase 12

2. SYNOPSIS 13
2.1. Synopsis 13
2.2. Schedule of Events 22

3. INTRODUCTION 25
3.1. Background and Rationale 25
3.1.1. Disease Information 25
3.1.2. Treatment Methods 25
3.1.3. Rationale for Study Conduct 26
3.2. Nonclinical Study Results 26
3.2.1. Efficacy Study Results 26
3.2.2. Pharmacokinetic Study Results 28
3.3. Previous Clinical Trial Results 29
3.4. Rationale for Dose Determination 29

4. STUDY OBJECTIVES 31

5. STUDY POPULATION 32
5.1. Planned Sample Size 32
5.2. Study Indication 32
5.3. Inclusion Criteria 32
5.3.1. Screening Inclusion Criteria 32
5.3.2. Randomization Inclusion Criteria 32
5.4. Exclusion Criteria 32
5.5. Premature Withdrawal 34
5.5.1. Definition 34
5.5.2. Criteria for Premature Withdrawal 34

5.5.3. Protocol Violation 34
5.5.4. Handling of Premature Withdrawals and Protocol Violations 35

6. STUDY DESIGN 36
6.1. Study Methods 36
6.2. Duration of Study 37
6.3. Randomization 38
6.4. Double Blinding and Unblinding 38

7. CRITERIA FOR END OF STUDY AND PREMATURE WITHDRAWAL 39
7.1. Criteria for End of Study 39
7.2. Study Discontinuation or Premature Termination 39

8. INVESTIGATIONAL PRODUCT INFORMATION AND MANAGEMENT 40
8.1. Active Ingredient, Strength, and Formulation of Investigational Product 40
8.1.1. Test Drug 40
8.1.2. Comparator 40
8.1.3. Rescue Medication 40
8.2. Investigational Product Packaging and Labeling 40
8.3. Route and Method of Administration 41
8.4. Investigational Product Accountability, Retrieval, and Disposal 41

9. ADMINISTRATION PLAN 43
9.1. Administration and Treatment Schedules 43
9.2. Concomitant Medication 43
9.3. Prohibited Drugs and Therapies 43
9.3.1. Prohibited Drugs 43
9.3.2. Prohibited Therapies 43
9.4. Rescue Medication 43
9.5. Treatment Compliance 44

10. STUDY PROCEDURES AND ASSESSMENTS 45
10.1. Assessment Items by Visit 45
10.1.1. Visit 1 (Screening/wash-out period, ~-3 weeks) 45
10.1.2. Visit 2 (Baseline [Randomization], Day 0) 45
10.1.3. Visit 3 (1 week [±3 days]) 46
10.1.4. Visit 4, Visit 5 (Double-blind treatment period, 4 weeks [±4 days], 8 weeks [±4 days]) 46

10.1.5. Visit 6 (EOT, 12 weeks [±4 days]) 47
10.1.6. Visit 7 (14 weeks [±4 days]) 48
10.1.7. Unscheduled Visits 48
10.2. Assessment Methods 48
10.2.1. Written Informed Consent and Screening Number Assigning 48
10.2.2. Demographics 48
10.2.3. Prior/Concomitant Medication and Treatment History 48
10.2.4. Medical and Surgical History 49
10.2.5. Basic Disease Information 49
10.2.6. Body Measurement 49
10.2.7. Vital Signs/Physical Examination 49
10.2.8. Laboratory Tests 49
10.2.9. Pregnancy Test 49
10.2.10. Electrocardiography 50
10.2.11. Assessment of Inclusion and Exclusion Criteria and Randomization 50
10.2.12. Efficacy Evaluation Methods 50
10.2.13. Ocular Safety Evaluation Methods 52
10.2.14. Rescue Medication Dispensing and Retrieving 53
10.2.15. Investigational Product Dispensing and Retrieving 54
10.2.16. Subject Diary Dispensing and Retrieving 54
10.2.17. Assessment of Adverse Events 54
10.2.18. Concomitant Medication and Treatment 54
10.3. Endpoints 54
10.3.1. Primary Efficacy Endpoint 54
10.3.2. Secondary Efficacy Endpoints 54
10.3.3. Safety Endpoints 55
10.4. Definition and Reporting of Adverse Events 55
10.4.1. Definition of Adverse Events 55
10.4.2. Expected Adverse Events with Investigational Product and Precautions for Use 56
10.4.3. Recording of Adverse Events 57
10.4.4. Actions to Be Taken for Adverse Events 58
10.4.5. Reporting Procedures for Adverse Drug Reactions 59
10.5. Pregnancy 60
10.6. Overdose 60

11. DATA ANALYSIS AND STATISTICAL CONSIDERATIONS 61
11.1. Analysis Sets 61
11.1.1. Efficacy Sets 61
11.1.2. Safety Set 61
11.2. Statistical Analysis Methods 61
11.2.1. General Principles for Analysis 61
11.2.2. Handling of Missing Values 61
11.2.3. Covariate Adjustment 61
11.2.4. Interim Analysis 62
11.2.5. Multiple Comparison or Multiplicity 62
11.2.6. Demographics and Other Baseline Characteristics 62
11.2.7. Efficacy Evaluation 62
11.2.8. Safety Evaluation 64
11.3. Rationale for Sample Size Determination 65

12. DATA MANAGEMENT 66
12.1. Data Collection/Access 66
12.2. Data Protection/Retention 66

13. ETHICAL CONSIDERATIONS AND ADMINISTRATIVE PROCEDURES 67
13.1. Applicable Laws and Regulations 67
13.2. Informed Consent 67
13.3. Subject Compensation Rules 67
13.4. Criteria for Post-Study Medical Care and Treatment 67
13.5. Compliance with Ethical Principles and Measures to Protect Subjects 67
13.5.1. Approval and Amendment of Study Plans 68
13.5.2. Protocol Violation 69
13.6. Publication 69
13.7. Confidentiality of Patient Records 69
13.8. Quality Control and Quality Assurance 69
13.8.1. Study Monitoring 69
13.8.2. Audits and Inspections 70

14. INFORMATION ON SPONSOR AND NAMES AND POSITIONS OF PRINCIPAL INVESTIGATORS 71
14.1. Names and Addresses of Institutions 71
14.2. Principal Investigators and Other Study Participants 71

14.3. Sponsor 71
14.4. Contract Research Organization 71

15. OTHERS REQUIRED FOR SAFE AND SCIENTIFIC STUDY CONDUCT 72

16. REFERENCES 73

[Table]

Table 1. Test drug 40
Table 2. Comparator 40
Table 3. Rescue medication 40
Table 4. IP administration method and duration of administration 41
Table 5. Oxford grading system 51
Table 6. Severity and duration of tolerance symptoms 52
Table 7. Criteria for the assessment of ocular hyperemia 52

[Figure]

Figure 1. Study Scheme 36

[List of Attachments]

Attachment 1. List of Institutions
Attachment 2. Subject Information Sheet and Informed Consent Form
Attachment 3. Subject Compensation Rules
Attachment 4. Subject Diary
Attachment 5. OSDI Questionnaire
Attachment 6. SPEED Questionnaire
Attachment 7. Ocular Tolerance Test

[Document History]

Document history including this protocol:

Version	Date	Summary of amendment
1.0	10Feb2022	Initial protocol
1.1	12Jul2022	**1) Title of study**<br>– A change was made to the title of the study to reflect the change of the test drug name.<br>**2) 5.4. Exclusion Criteria**<br>– Complete abstinence was added to acceptable methods of birth control.<br>**3) 6.1. Study Methods**<br>– A change was made to incorporate that, if both eyes have the same test results at baseline, the right eye (OD) will be selected as the study eye.<br>**4) 6.4. Double Blinding and Unblinding, 8.4. Investigational Product Accountability, Retrieval, and Disposal**<br>– Change of IP packaging was reflected.<br>**5) Entire document including 8.1.1. Test Drug**<br>– Changes of the test drug name, manufacturer, appearance, and formulation were reflected.<br>**6) 10.2.11. Efficacy Evaluation Methods**<br>– A change was made to 10.2.11.3. Corneal Staining to reflect the use of staining reagent strips.<br>– A change was made to 10.2.11.6. Schirmer's Test Without Anesthesia to reflect the use of colored strips.<br>**7) 10.2.12. Safety Evaluation Methods**<br>- Symptoms were added to 10.2.12.1. Ocular Tolerance Assessment.<br><br>To improve clarity and consistency, minor changes were made across the protocol.
1.2	03Aug2022	**1) 3.2.1. Nonclinical Efficacy Study Results**<br>– Content was supplemented with specific values.<br>**2) 8.1.1. Test Drug**<br>– Change of the company name of the manufacturer was reflected.
1.3	10Aug2022	**1) 8.1.1. Test Drug**<br>– Change of the company name of the manufacturer was reflected.
2.0	06Oct2022	**1) 5.4. Exclusion Criteria**<br>– Changes or additions were made to drugs administered prior to screening and permitted period, permitted period for history of intraocular surgery, criteria for screening tests, criteria for medical history, acceptable methods of birth control, and duration of birth control.<br>**2) 5.5.2. Criteria for Premature Withdrawal**<br>– Protocol violation was added to criteria for premature withdrawal.<br>**3) 5.5.3. Protocol Violation**<br>– Specific items of protocol violations were added.<br>**4) 6.1. Study Methods**<br>– A visit (Visit 3, 1 week) was established for ophthalmologic examination and safety follow-up after IP treatment.<br>– A visit (Visit 7, 14 weeks) was established for safety follow-up after EOT.<br>**5) 6.2. Duration of Study**<br>– A change was made to the duration of study to reflect the additional safety follow-up visits.<br>**6) 6.3. Randomization**<br>– A change was made to the composition of a screening number.<br>**7) 7.1. Criteria for End of Study, 10.2.17 Assessment of Adverse Events, 10.4. Definition and Reporting of Adverse Events, 10.4.4. Actions to Be Taken for**

		**Adverse Events**<br>– Changes were made to text to reflect the additional safety visits.<br>**8) 9.3.1. Prohibited Drugs**<br>– Inhaled steroids was additionally removed, and a change was made to allow topical steroid ointments for any purpose other than for eyes.<br>**9) 10.1. Assessment Items by Visit**<br>– Text was added to reflect the addition of a visit (Visit 3) for ophthalmologic examination and safety follow-up after the first IP dose.<br>– Text was added to reflect the addition of a safety follow-up visit after EOT.<br>– Additional test items for individual visits were reflected.<br>**10) 10.2.4. Medical and Surgical History**<br>– Text was added to incorporate the collection of information on malignant tumors.<br>**11) 10.2.8. Laboratory Tests**<br>– HbA1c and serum hepatitis tests at screening were added.<br>**12) 10.2.10. Electrocardiography** and **10.2.13.5. Ocular Hyperemia Examination** were added.<br>**13) 10.3.2. Secondary Efficacy Endpoints**<br>– Proportion of subjects with a ≥ 10 mm/5 min increase from baseline in tear production (Schirmer's test) at Week 4, Week 8, and Week 12 was added as a secondary efficacy endpoint.<br>**14) 10.3.3. Safety Endpoints**<br>– Ocular hyperemia examination was added as a safety endpoint.<br>**15) 11.2.7. Efficacy Evaluation**<br>– Changes were made to analysis methods for efficacy endpoints.<br>**16) 11.2.8.4. Ophthalmologic Examination (Intraocular Pressure, Fundus Examination and Slit Lamp Microscopy, Ocular Hyperemia Assessment)**<br>– Changes were made to analysis methods to reflect the additional safety endpoints.<br><br>To improve clarity and consistency, minor changes were made across the protocol.
2.1	05Dec2022	**1) 5.5.4. Handling of Premature Withdrawals and Protocol Violations**<br>– The duration of AE follow-up in subjects who are prematurely withdrawn was changed to up to 2 weeks from the last dose of IP.<br>**2) 7.1. Criteria for End of Study**<br>– A change was made to the criteria for EOS to reflect the decision to perform the safety follow-up visit (Visit7) as an on-site visit.<br>**3) 10.1. Assessment Items by Visit, 10.2. Assessment Methods**<br>– A change was made to mandate a pregnancy test at Visit 2 (baseline) in women of childbearing potential.<br>– A change was made to perform Visit 7 (14 weeks [±4 days]) as an on-site visit for safety evaluation and laboratory tests (if necessary).<br>– Ocular tolerance assessment was added as an assessment item for Visit 7.<br>**4) 10.2.17 Assessment of Adverse Events, 10.4. Definition and Reporting of Adverse Events, 10.4.4. Actions to Be Taken for Adverse Events**<br>– A change was made to the duration of AE collection.<br>**5) 11.2.3. Covariate Adjustment**<br>– 11.2.7. A change was made to ensure consistency with covariate adjustment described in efficacy evaluation.
2.2	09Dec2022	**1) 5.4. Exclusion Criteria**<br>– A change was made to the wordings of acceptable methods of birth control in exclusion criterion 22.
2.3	20Feb2023	**1) 10.3.2. Secondary Efficacy Endpoints, 11.2.3. Covariate Adjustment, 11.2.7.2. Secondary Efficacy Evaluation**<br>- A change was made to one of the secondary endpoints as "proportion of subjects with a ≥ 10 mm/5 min increase <u>or above</u> from baseline in tear production (Schirmer's test) at Week 4, Week 8, and Week 12".

[Glossary of Terms and Abbreviations]

Abbreviation	Full Term
ADR	Adverse Drug Reaction
AE	Adverse Event
ALT	Alanine Aminotransferase
AST	Aspartate Aminotransferase
BUN	Blood Urea Nitrogen
CIOMS	Council for International Organizations of Medical Sciences
CPK	Creatine Phosphokinase
CRO	Contract Research Organization
CSR	Clinical Study Report
DED	Dry Eye Disease
eCRF	Electronic Case Report Form
EOS	End of Study
EOT	End of Treatment
FDA	Food and Drug Administration
GCP	Good Clinical Practice
γ-GTP	Gamma-Glutamyl Transpeptidase
IP	Investigational Product
IPL	Intense Pulsed Light
IRB	Institutional Review Board
IWRS	Interactive Web Response System
LLOQ	Low Limit Of Quantitation
LSM	Least Squares Mean
MedDRA	Medical Dictionary for Regulatory Activities
MGD	Meibomian Gland Dysfunction
MOASIS™	Molecular Associated Innovative Substance
NZW	New Zealand White
OSDI	Ocular Surface Disease Index
PT	Preferred Term
RBC	Red Blood Cell
SAE	Serious Adverse Event
SD	Standard Deviation
SE	Standard Error
SOC	System Organ Class
SOP	Standard Operating Procedure
SPEED	Standard Patient Evaluation of Eye Dryness Questionnaire
TBUT	Tear Film Break-Up Time
TEAE	Treatment Emergent Adverse Event
UAE	Unexpected Adverse Event
UV	Unscheduled Visit
VDT	Visual Display Terminals
WBC	White Blood Cell

1. Title and Phase of Study

1.1. Title of Study

A Multicenter, Active control, Parallel group, Randomized, Double-blind, Phase II Clinical Trial to Evaluate the Efficacy and Safety of SCAI-001 Eye Drops in Patients with Dry Eye Disease

1.2. Phase

Phase 2

2. Synopsis

2.1. Synopsis

<table>
<tr><td>Study Title</td><td>A Multicenter, Active control, Parallel group, Randomized, Double-blind, Phase II Clinical Trial to Evaluate the Efficacy and Safety of SCAI-001 Eye Drops in Patients with Dry Eye Disease</td></tr>
<tr><td>Protocol Identifier</td><td>SCAI-001-201</td></tr>
<tr><td>Sponsor</td><td>SCAI Therapeutics</td></tr>
<tr><td>Institutions and Principal Investigators</td><td>Approximately 11 institutions in South Korea including Pusan National University Hospital
Coordinating investigator: Professor Jong Soo Lee, Pusan National University</td></tr>
<tr><td>Phase and Design of Study</td><td>A multicenter, active control, parallel group, randomized, double-blind, Phase II clinical trial</td></tr>
<tr><td>Duration of Study</td><td>Approximately 17 months from the date of approval by the Ministry of Food and Drug Safety and/or Institutional Review Board
Subject enrollment: Approximately 6 months
Duration of study participation for individual subjects: Approximately 5 months (1 week of screening + 2 weeks of wash-out period + 12 weeks of treatment period + 2 weeks of safety follow-up period)
End of last subject's follow-up ~ preparation of the clinical study report: Approximately 6 months</td></tr>
<tr><td>Objectives</td><td>The objectives of the study are to demonstrate the efficacy and safety of SCAI-001 eye drops (cyclosporine 0.01% or 0.02%) based on the evaluation of efficacy and safety in comparison with Restasis® (cyclosporine 0.05%), the active comparator, and to explore the appropriate doses of SCAI-001 eye drops in patients with dry eye disease (DED).</td></tr>
<tr><td>Study Indication</td><td>DED</td></tr>
<tr><td>Sample Size</td><td>This study will enroll a total of 114 subjects (38 subjects per group) with a 20% drop-out rate taken into consideration.</td></tr>
<tr><td>Investigational Products and Rescue Medication</td><td><u>Test drug and comparator</u>
<table>
<tr><th></th><th>Test drugs 1, 2</th><th>Comparator</th></tr>
<tr><td>Product name (code name):</td><td>Test drug 1: SCAI-001 eye drops 0.01%
Test drug 2: SCAI-001 eye drops 0.02%</td><td>Restasis® 0.05%</td></tr>
<tr><td>Manufacturer:</td><td>DHP Korea Co., Ltd.</td><td>Allergan Korea Ltd.</td></tr>
<tr><td>Active ingredient and strength:</td><td>Cyclosporine 0.1 mg/mL or 0.2 mg/mL</td><td>Cyclosporine 0.5 mg/mL</td></tr>
<tr><td>Appearance and formulation:</td><td>Clear, colorless to slightly white liquid for eye drops in a clear, colorless plastic container</td><td>White, opaque to slightly translucent, homogeneous liquid in a white, translucent container</td></tr>
<tr><td>Shelf-life:</td><td>24 months from the date of manufacture</td><td>24 months from the date of manufacture</td></tr>
<tr><td>Storage conditions:</td><td>Store in an air tight container at ambient temperature (1~30 ℃).</td><td>Store in an air tight container at room temperature (15~25 ℃).</td></tr>
</table>
<u>Rescue medication</u></td></tr>
</table>

	**Rescue medication**<br>Product name (code name): Refresh Plus™<br>Manufacturer: Allergan Korea Ltd.<br>Active ingredient and strength: Carboxymethylcellulose sodium 2 mg/0.4 mL<br>Appearance and formulation: Clear, colorless to pale yellow eye drops in a clear, single-use container<br>Shelf-life: 24 months from the date of manufacture<br>Storage conditions: Store in an air tight container at room temperature (15~25 ℃).
**Dosage, Mode of Administration, and Duration of Administration**	Once subjects who meet the inclusion and exclusion criteria are randomized in a 1:1:1 ratio to the test groups (test group 1 or test group 2) or the comparator group, they will administer investigational product (IP) according to their assigned group for 12 consecutive weeks, twice a day, as follows:<br>• Test group 1: Test drug 1 (SCAI-001 eye drops 0.01%)<br>• Test group 2: Test drug 2 (SCAI-001 eye drops 0.02%)<br>• Comparator group: Comparator (Restasis® 0.05%)<br><br>Classification / Test group 1 / Test group 2 / Comparator group<br>Morning: ○ / ◎ / △<br>Afternoon: ○ / ◎ / △<br><br>**○: Test drug 1(SCAI-001 eye drops 0.01%)**<br>**◎: Test drug 2(SCAI-001 eye drops 0.02%)**<br>**△: Comparator Restasis® 0.05%)**<br><br>**Treatment Methods:**<br>Randomized subjects will administer IP of the respective group to both eyes, twice daily, one drop at a time. Before use, IP is to be mixed by inverting its container. The first IP dose will be administered in the afternoon of the baseline day. Recommended IP dosing time is 9 AM (±1 hour) and 9 PM (±1 hour), twice daily at an interval of 12 hours. IP will be administered to the conjunctival sac by pulling down the lower eye lid with a finger, with eyes facing upwards by tilting head back. To ensure that the administered drug does not run down, subjects will maintain the position for 30 seconds with eyes closed, followed by blinking twice or three times so that the drug can spread evenly.<br>When IP and rescue medication are to be used simultaneously, they should be administered with 15 minutes apart.<br>Treatment history of IP and rescue medication will be recorded in the subject diary.
**Inclusion Criteria**	**Screening inclusion criteria**<br>To be enrolled in this study, subjects must meet all of the following inclusion criteria at the screening visit.<br><br>1. Male and female adult at the age of ≥ 19 years old<br>2. Individual with dry eye symptoms (sandiness/grittiness, dryness, light sensitivity, pain or soreness, blurred vision, etc.)

	3. Individual whose either or both eyes meet all of the following criteria at screening:<br>1) Inflammation in cornea and conjunctiva with corneal staining score - Oxford grading ≥ 2<br>2) Schirmer's test without anesthesia ≤ 10 mm/5 min (If Schirmer's test without anesthesia is 0 mm/5 min, nasal stimulation Schirmer's test in the same eye must be ≥ 3 mm/5 min.)<br>3) Tear film break-up time (TBUT) ≤ 10 seconds<br>4. Individual with best corrected visual acuity ≥ 0.2 in both eyes<br>5. Individual who is able to understand the study and voluntarily provides written informed consent<br><br>**Randomization inclusion criteria**<br>To be enrolled in this study, subjects must meet all of the following inclusion criteria at the baseline visit.<br>1. Individual with dry eye symptoms (sandiness/grittiness, dryness, light sensitivity, pain or soreness, blurred vision, etc.)<br>2. Individual whose eye that has met the following criteria at screening meets all of the following criteria (In the case that both eyes have met all of the criteria at screening, the subject will be eligible if at least either eye meets all of the following criteria at randomization.):<br>1) Inflammation in cornea and conjunctiva with corneal staining score - Oxford grading ≥ 2<br>2) Schirmer's test without anesthesia ≤ 10 mm/5 min (If Schirmer's test without anesthesia is 0 mm/5 min, nasal stimulation Schirmer's test in the same eye must be ≥ 3 mm/5 min.)<br>3) TBUT ≤ 10 seconds<br>3. Individual with best corrected visual acuity ≥ 0.2 in both eyes
**Exclusion Criteria**	Individuals who meet any of the following exclusion criteria will be excluded from study enrollment.<br><br>1. Individual with clinically significant ophthalmologic diseases (corneal surface disease, abnormal corneal sensitivity, abnormal dacryorrhea, etc.) not attributed to DED<br>2. Individual who has a history of treatment with systemic steroids and immunosuppressants (azathioprine, tacrolimus, cyclosporine, mycophenolate mofetil, etc.) within 90 days prior to screening<br>3. Individual who has a history of treatment with cyclosporine eye drops within 6 weeks prior to screening<br>4. Individual who has a history of treatment with any anti-inflammatory drugs for DED[a] other than cyclosporine eye drops within 30 days prior to screening<br>[a] Anti-inflammatory drugs for DED: Steroidal or non-steroidal anti-inflammatory eye drops, autologous serum eye drops, mast cell stabilizers (including eye drops), etc.<br>5. Individual who has a history of treatment with any other drugs that may affect DED [b] within 30 days prior to screening. However, those who have been treated with such drugs for ≥ 30 days prior to screening with the same dosage and administration and will maintain the treatment with the same dosage and administration throughout the study will be allowed to participate in the study.<br>[b] Other drugs that may affect DED: Oral contraceptives, anticholinergics, tricyclic antidepressants, antihistamines, hypnotics, diuretics, antimuscarinics, beta blockers, retinoids, aspirin, or non-steroidal anti-inflammatory drugs (NSAIDs) (For non-steroidal anti-inflammatory eye drops, see exclusion criterion 4.), etc.<br>6. Individual who has worn contact lenses within 72 hours prior to screening or who needs

	to wear them during the study<br>7. Individual who has a history of intraocular surgery within 12 months prior to screening<br>8. Individual who has a history of intraocular procedures, such as punctal plug, lacrimal punctal occlusion, cauterization of the punctum, ophthalmologic intense pulsed light (IPL) laser, etc., within 90 days prior to screening<br>9. Individual who has a history of vision correction surgery, such as LASIK, LASEK, etc., within 12 months prior to screening<br>10. Individual with symptoms of active eye infection such as active allergy, anterior uveitis, Stevens-Johnson syndrome, etc.<br>11. Patient with autoimmune diseases such as Sjogren's syndrome, etc.<br>12. Individual who has a history of corneal transplant or neurotrophic keratitis<br>13. Individual who has a history of hepatitis B or C<br>14. Individual who has a history of malignant tumors within 5 years prior to screening (However, those with adequately treated ['interventional procedure' or 'considered inactive based on the judgment of the investigator'] basal or squamous epithelial cell skin cancer or cervical intraepithelial neoplasia will be allowed to participate in the study.)<br>15. Uncontrolled hypertension (> 160/100 mmHg) at the screening or baseline visit or insulin dependent or uncontrolled diabetes mellitus (HbA1c > 8% based on the screening test) at screening<br>16. Individual with intraocular pressure (IOP) > 21 mmHg in either or both eyes or who is diagnosed with glaucoma<br>17. Individual who is expected to require an intraocular procedure or surgery during the study, based on the judgment of the investigator<br>18. Individual with any of the following laboratory test values:<br>1) Creatinine level ≥ 2 x site-specific upper limit of normal (ULN)<br>2) ALT or AST level 2 x ULN<br>19. Individual with hypersensitivity reactions to any of the components of IP<br>20. Individual with mental disorders<br>21. Pregnant or lactating woman<br>22. Woman who plans to become pregnant or who has a positive pregnancy test result at screening, or woman of childbearing potential or man who does not agree to use at least one of the effective, medically acceptable methods of birth control* from screening to 14 days after the end of IP treatment (During this period, egg and sperm donation will be prohibited for female and male subjects, respectively.)<br>* Medically acceptable methods of birth control: Intrauterine device (loop, Mirena, etc.), double barrier methods in which both man and woman use contraceptives (condom/femidom or diaphragm or cervical cap), non-oral contraceptives (injection, contraceptive ring, patch, etc.), complete abstinence.<br>Oral contraceptives and periodic abstinence (e.g., basal body temperature method, natural cycles method, etc.) alone will not be allowed.<br>However, women who had menopause (no menstruation for ≥ 1 year) prior to study participation or surgically sterilized (vasectomy, bilateral tubal ligation, etc.) women or men will be allowed to participate in the study without consent to birth control.<br>23. Individual who has a history of drug or alcohol abuse<br>24. Individual who participated in another clinical trial within 30 days prior to screening<br>25. Individual who is ineligible for study participation based on the judgment of the investigator
**Methodology**	This is a multicenter, active control, parallel group, randomized, double-blind, Phase II clinical trial to evaluate the efficacy and safety of SCAI-001 eye drops in patients with DED.

	**※ Study scheme**

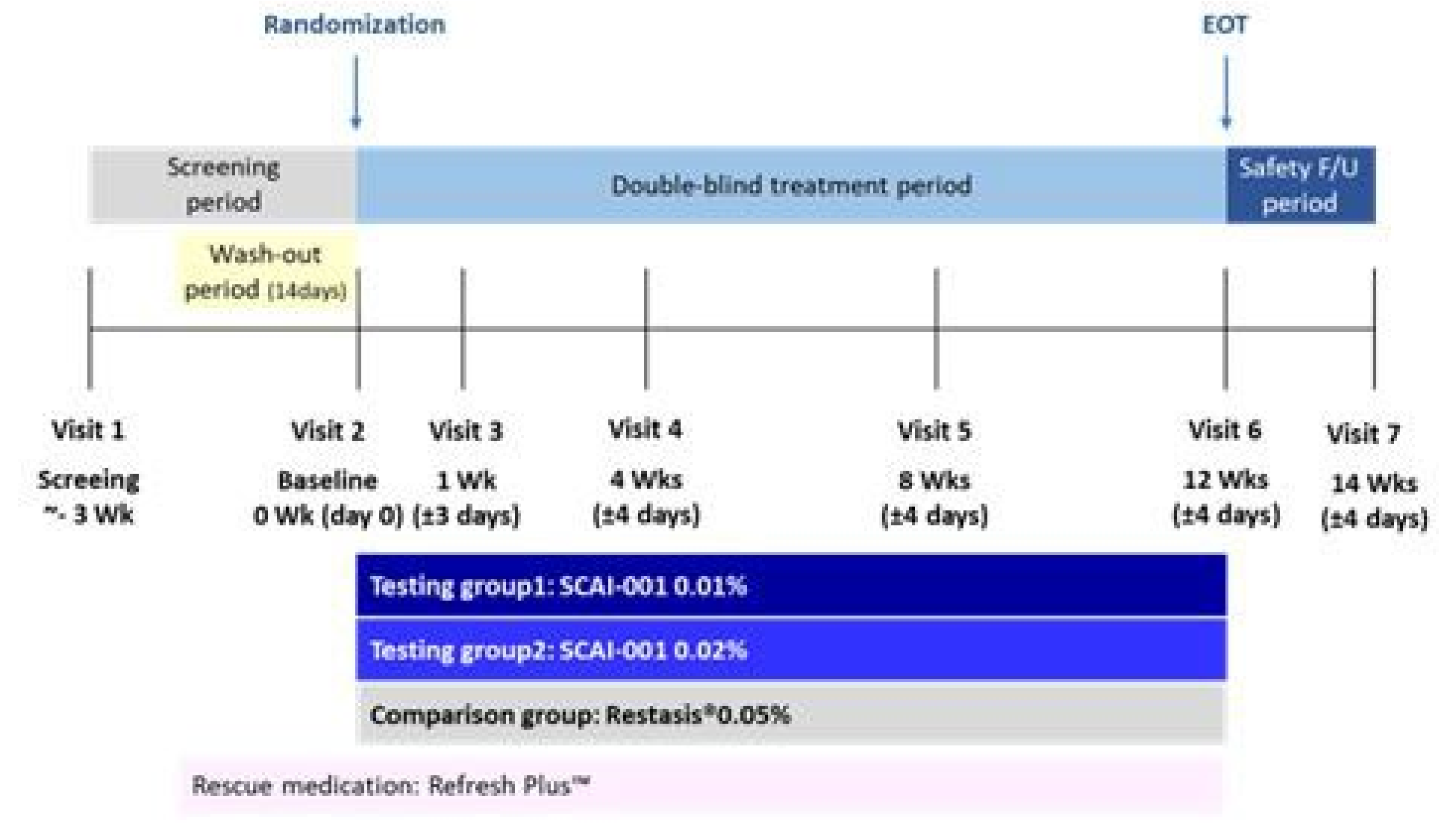

Screening period (Visit 1, ~-1 week) and wash-out period (~-2 weeks)
Subjects who voluntarily sign the informed consent form at the screening visit (Visit 1) will undergo screening tests. After the screening tests, those who are determined eligible based on the inclusion and exclusion criteria will undergo a wash-out period during which DED treatment the subject has been on within 1 week will be discontinued for at least 2 weeks. If there is no ongoing DED treatment at the start of study participation, the wash-out period may be skipped. During the wash-out period, the existing DED treatment will be stopped, and if necessary, rescue medication (Refresh Plus™) provided in the study may be administered. However, rescue medication is not to be administered from the day before the baseline visit (randomization). Rescue medication is to be administered 1~2 drops at a time, no more than 3 times a day, if possible. When rescue medication is used, treatment information (date of treatment, treated eye, etc.) will be recorded in the subject diary.

Double blind treatment period (Visit 2~Visit 6, 0~12 weeks)

1) Baseline (Visit 2, 0 week)

At the baseline visit (Visit 2), subjects will be re-assessed for the inclusion and exclusion criteria, and those who are determined eligible for study participation will be randomized in a 1:1:1 ratio to the test group 1 (SCAI-001 eye drops 0.01%) or the test group 2 (SCAI-001 eye drops 0.02%) or the comparator group (Restasis® 0.05%). Based on the baseline test results, the study eye will be selected. The study eye will be the eye with the worse corneal staining results, i.e., the higher corneal staining score. If both eyes have the same corneal staining score, the study eye will be the eye with the worse Schirmer's test results without anesthesia, i.e., the smaller tear production without anesthesia. If both eyes have the same Schirmer's test results without anesthesia, the right eye (oculus dexter) will be the study eye.
In the morning of the baseline day, IP will not be administered, and the first dose of IP will be administered in the afternoon of the baseline day. On the baseline day, rescue medication may be used after all tests are done on that day.

Subjects who are assigned to the test group 1 or the test group 2 or the comparator group by randomization will administer IP to both eyes, twice daily at an interval of 12 hours, one drop at a time. Recommended IP dosing time is 9 AM (±1 hour) and 9 PM (±1 hour). Treatment information is to be recorded in the subject diary.
While participating in the study, subjects should complete the subject diary dispensed in this

	study. In the subject diary, treatment history of IP and rescue medication is to be recorded. The subject diary will be dispensed to subjects at baseline (Visit 2), Week 4 (Visit 4), and Week 8 (Visit 5) and retrieved at the respective next visit (The diary dispensed at Visit 2 will be retrieved at Visit 4) to check treatment history of IP and rescue medication.<br><br>**<u>2) Visit 3 (1 week)</u>**<br>Following the first IP dose at baseline, subjects will visit the institution at Week 1 (Visit 3) after morning dose of IP (if necessary, rescue medication). At this visit, subjects will be assessed for concomitant medication, vital signs for ophthalmologic examination, physical examination, laboratory tests (if necessary), ocular tolerance, best corrected visual acuity, fundus examination, slit lamp microscopy, ocular hyperemia examination, and IOP and will be checked for adverse events (AEs).<br><br>**<u>3) Visit 4~6 (EOT) (4~12 weeks)</u>**<br>For the evaluation of efficacy and safety, subjects will visit the institution after morning dose of IP (if necessary, rescue medication) at Week 4 (Visit 4), Week 8 (Visit 5), and Week 12 (Visit 6) from the start of IP treatment. Ophthalmologic examination will be performed at least 2 hours after IP dosing (and rescue medication).<br>During the on-site visits, the following activities will be performed as scheduled: Schirmer's test without anesthesia, corneal staining, conjunctival staining, TBUT, Standard Patient Evaluation of Eye Dryness (SPEED) questionnaire, Ocular Surface Disease Index (OSDI), and concomitant medication for efficacy evaluation; vital signs, physical examination, laboratory tests (at Visit 6 only), pregnancy test (at Visit 6 only), ocular tolerance, best corrected visual acuity, fundus examination, slit lamp microscopy, ocular hyperemia examination, IOP, and checking for AEs for safety evaluation; and retrieving unused IP and rescue medication, dispensing IP and rescue medication (at Visits 4 and 5 only), and retrieving the subject diary and dispensing a new diary (at Visits 4 and 5 only).<br><br>All subjects will be treated with IP for 12 weeks. During IP treatment, additional tests may be performed, besides the prescheduled tests, at the discretion of the investigator (e.g., unacceptable level of worsening of symptoms), and IP treatment may be discontinued at the discretion of the investigator.<br>End of treatment (EOT) is defined as a visit (Visit 6) to be performed 12 weeks after baseline (Visit 2). At EOT, subjects should visit the institution after morning dose of IP (if necessary, rescue medication), and ophthalmologic examination will be performed at least 2 hours after that.<br><br>**<u>※ Rescue medication</u>**<br>Whenever necessary, subjects may administer rescue medication (Refresh Plus™) provided in the study. However, it is prohibited to administer rescue medication within 15 minutes before and after IP dosing. Rescue medication is to be administered 1~2 drops at a time, no more than 3 times a day, if possible. When rescue medication is used, treatment information (date of treatment, treated eye, etc.) will be recorded in the subject diary.<br>At baseline (Visit 2), Week 4 (Visit 4), and Week 8 (Visit 5), a sufficient amount of rescue medication that can be used until the next visit (For Visit 2, the amount that can be used until Visit 4) will be provided for subjects, and any unused rescue medication will be retrieved at the subsequent visits (Visits 4, 5, and 6).<br><br>**<u>Safety follow-up period (Visit 7, 14 weeks)</u>**<br>Subjects will visit the institution 2 weeks after EOT for safety follow-ups and undergo safety evaluation including ophthalmologic examination and laboratory tests (if necessary).
**Prohibited Drugs and Therapies**	**<u>Prohibited drugs</u>**<br>1. Any ophthalmic drugs that may affect the results of the study (e.g., steroidal/non-steroidal

	anti-inflammatory eye drops, etc.)<br>2. Cyclosporine eye drops<br>3. Systemic steroids; and local steroids such as ophthalmic, inhaled, or subcutaneously injected steroids (Use of topical steroid ointments for any purpose other than for eyes will be permitted.)<br>4. Immunosuppressants (azathioprine, tacrolimus, cyclosporine, mycophenolate mofetil, etc.)<br>5. Drugs that may affect DED (oral contraceptives, anticholinergics, tricyclic antidepressants, antihistamines, hypnotics, diuretics, antimuscarinics, beta blockers, retinoids, aspirin, NSAIDs, etc.). However, drugs that have been administered for ≥ 30 days prior to screening without change of dosage and administration will be permitted, provided that they will be administered throughout the study with the same dosage and administration.<br>6. Health supplements (vitamin A, lutein, omega-3, snake oil, etc.) that may affect DED<br><br>**Prohibited therapies**<br>1. Intraocular procedure and surgery during the study<br>2. Use of contact lenses during the study<br>3. Use of punctual plugs<br>4. Use of other IP or investigational device
**Efficacy Endpoints**	**1. Primary efficacy endpoint**<br>Change from baseline in tear production (Schirmer's test) at Week 12<br><br>**2. Secondary efficacy endpoints**<br>1) Change from baseline in tear production (Schirmer's test) at Week 4 and Week 8<br>2) Proportion of subjects with a ≥ 10 mm/5 min increase or above from baseline in tear production (Schirmer's test) at Week 4, Week 8, and Week 12<br>3) Change from baseline in corneal staining score – Oxford grading at Week 4, Week 8, and Week 12<br>4) Change from baseline in conjunctival staining score – Oxford grading at Week 4, Week 8, and Week 12<br>5) Change from baseline in TBUT at Week 4, Week 8, and Week 12<br>6) Change from baseline in DED questionnaire score (SPEED) at Week 4, Week 8, and Week 12<br>7) Change from baseline in OSDI at Week 4, Week 8, and Week 12<br>8) Total number of administered doses of rescue medication during the study
**Safety Endpoints**	1) Treatment emergent AEs (TEAEs): Including adverse drug reactions (ADRs), serious AEs (SAEs), serious ADRs (SADRs), unexpected AEs (UAEs), and unexpected ADRs (UADRs)<br>2) Ocular tolerance test<br>3) Best corrected visual acuity<br>4) Ophthalmologic examination (IOP, fundus examination, slit lamp microscopy, and ocular hyperemia examination)<br>5) Vital signs<br>6) Laboratory tests
**Statistical Methods**	**1. Primary efficacy evaluation**<br>For change from baseline in tear production (Schirmer's test) at Week 12, descriptive statistics (number of subjects, mean, standard deviation, median, minimum, and maximum) will be presented by treatment group. To test the difference between each test group and the comparator group (test group 1 vs comparator group, test group 2 vs comparator group), analysis of

covariance (ANCOVA) will be performed with adjustment for baseline and institution. ANCOVA results will be summarized with least squares mean (LSM) and standard error (SE) by treatment group, and LSM difference between the treatment groups and its 95% confidence interval (CI) with two-sided *p*-value.

2. Secondary efficacy evaluation

1) Change from baseline in tear production (Schirmer's test) at Week 4 and Week 8

For change from baseline in tear production (Schirmer's test) at Week 4 and Week 8, descriptive statistics (number of subjects, mean, standard deviation, median, minimum, and maximum) will be presented by treatment group. To test the difference between each test group and the comparator group (test group 1 vs comparator group, test group 2 vs comparator group), ANCOVA will be performed with adjustment for baseline and institution. ANCOVA results will be summarized with LSM and SE by treatment group, and LSM difference between the treatment groups and its 95% CI with two-sided *p*-value.

2) Proportion of subjects with a ≥ 10 mm/5 min increase or above from baseline in tear production (Schirmer's test) at Week 4, Week 8, and Week 12

Proportion of subjects with a ≥ 10 mm/5 min increase or above from baseline in tear production (Schirmer's test) at Week 4, Week 8, and Week 12 and its exact 95% CI will be presented. To test the difference between each test group and the comparator group (test group 1 vs comparator group, test group 2 vs comparator group), Cochran-Mantel-Haenszel test will be performed with institution as a covariate.

3) Change from baseline in corneal staining score – Oxford grading at Week 4, Week 8, and Week 12

4) Change from baseline in conjunctival staining score – Oxford grading at Week 4, Week 8, and Week 12

5) Change from baseline in TBUT at Week 4, Week 8, and Week 12

6) Change from baseline in DED questionnaire score (SPEED) at Week 4, Week 8, and Week 12

7) Change from baseline in OSDI at Week 4, Week 8, and Week 12

For endpoints 3) to 7), descriptive statistics (number of subjects, mean, standard deviation, median, minimum, and maximum) will be presented by treatment group. To test the difference between each test group and the comparator group (test group 1 vs comparator group, test group 2 vs comparator group), ANCOVA will be performed with adjustment for baseline and institution. ANCOVA results will be summarized with LSM and SE by treatment group, and LSM difference between the treatment groups and its 95% CI with two-sided *p*-value.

8) Total number of administered doses of rescue medication during the study

For total number of administered doses of rescue medication during the study, descriptive statistics (number of subjects, mean, standard deviation, median, minimum, and maximum) will be presented by treatment group. To test the difference between each test group and the comparator group (test group 1 vs comparator group, test group 2 vs comparator group), ANCOVA will be performed with adjustment for institution. ANCOVA results will be summarized with LSM and SE by treatment group, and LSM difference between the treatment groups and its 95% CI with two-sided *p*-value.

	**3. Safety evaluation**<br>AEs will be analyzed based on TEAEs. For AEs, ADRs, SAEs, and SADRs, number of subjects, incidence and its 95% CI will be presented by treatment group. To test the difference between each test group and the comparator group (test group 1 vs comparator group, test group 2 vs comparator group) in the incidence of AEs, Pearson's chi-square test or Fisher's exact test will be performed. AEs, ADRs, SAEs, and SADRs will be coded by System Organ Class (SOC) and Preferred Term (PT) using Medical Dictionary for Regulatory Activities (MedDRA); For coded AEs, number of subjects, incidence, and number of events will be presented by treatment group. In addition, status of AEs by severity and by relationship to IP will also be presented.<br><br>For other safety endpoints (ocular tolerance test, best corrected visual acuity, ophthalmologic examination [IOP, fundus examination and slit lamp microscopy, ocular hyperemia assessment], vital signs, and laboratory tests), intra-group and inter-group comparisons will be performed. For continuous variables, descriptive statistics (number of subjects, mean, standard deviation, median, minimum, and maximum) will be presented by treatment group for test values at each visit and for change from baseline. To test the intra-group difference before and after IP treatment, paired *t*-test or Wilcoxon's signed-rank test will be performed. To test the difference in test values at each visit or change from baseline between each test group and the comparator group (test group 1 vs comparator group, test group 2 vs comparator group), two sample *t*-test or Wilcoxon's rank sum test will be performed depending on satisfaction of normality assumption. For categorical variables, change from baseline at Week 12 will be presented as a shift table by treatment group, and intra-group comparison will be performed using McNemar's test or Bowker's test.

2.2. Schedule of Events

	Screening/wash-out period (14days)[1]	Baseline (Randomization)	Double-blind treatment period			EOT[21]	Safety follow up[22]	-
Visit Number	Visit 1	Visit 2	Visit 3	Visit 4	Visit 5	Visit 6	Visit 7	UV[23]
Week / Procedure	~-3 week (~-21 Days)	0 week (Day 0)	1 week (±3 days)	4 weeks (±4 days)	8 weeks (±4 days)	12 weeks (±4 days)	14 weeks (±4 days)	-
Written informed consent[2]	○							
Screening number assigning	○							
Demographics[3]	○							
Prior/concomitant medication and treatment history[4]	○	○	○	○	○	○	○	○
Medical/surgical history[5]	○							
Basic disease information[6]	○							
Body measurement[7]	○							
Vital signs/physical examination [8]	○	○	○	○	○	○	○	(○)
Laboratory tests [9]	○		(○)			○	(○)	(○)
Pregnancy test[10]	○	○				○		(○)
ECG[11]	○							
SPEED questionnaire		○		○	○	○		(○)
OSDI		○		○	○	○		(○)
Ocular tolerance			○	○	○	○	○	○
Best corrected visual acuity	○	○	○	○	○	○	○	(○)
Fundus examination and slit lamp microscopy	○	○	○	○	○	○	○	(○)
Ocular hyperemia examination	○	○	○	○	○	○	○	(○)
TBUT[12]	○	○		○	○	○		(○)
Corneal staining[13]	○	○		○	○	○		(○)
Conjunctival staining[14]		○		○	○	○		(○)
Schirmer's test without anesthesia	○	○		○	○	○		(○)
IOP[15]	○	○	○	○	○	○	○	(○)
Inclusion and exclusion criteria checking	○	○						
Study eye selection[16]		○						
Randomization number assigning		○						
Rescue medication dispensing[17]	○	○		○	○			

	Screening/wash-out period (14days)1)	Baseline (Randomization)	Double-blind treatment period			EOT21)	Safety follow up22)	-
**Visit Number**	**Visit 1**	**Visit 2**	**Visit 3**	**Visit 4**	**Visit 5**	**Visit 6**	**Visit 7**	**UV23)**
**Procedure** / **Week**	~-3 week (~-21 Days)	0 week (Day 0)	1 week (±3 days)	4 weeks (±4 days)	8 weeks (±4 days)	12 weeks (±4 days)	14 weeks (±4 days)	-
Unused rescue medication retrieving17)		○		○	○	○		
IP dispensing18)		○		○	○			
Unused IP retrieving18)				○	○	○		
Subject diary dispensing19)	○	○		○	○			
Subject diary retrieving19)		○		○	○	○		
AEs checking20)		○	○	○	○	○	○	○

EOT: end of treatment, UV: unscheduled Visit

Items marked with (○) may be performed during the visit if necessary at the discretion of the investigator.

1) Screening/wash-out period (14 days) — The screening visit (Visit 1) tests will be performed within 3 weeks prior to IP treatment and within 1 week prior to the wash-out period.
Subjects who are eligible for study participation at the screening visit will undergo at least 2 weeks of a wash-out period prior to the baseline visit (Visit 2).
If a wash-out period is not required, the screening visit and the baseline visit may be performed simultaneously.
2) Written informed consent — Written informed consent must be obtained prior to initiation of any of the study procedures on the screening day.
3) Demographics — Demographic information (initials, age, month and year of birth, and childbearing potential) will be collected.
4) Prior/concomitant medication and treatment history — Prior medication/treatment history within 30 days prior to the screening visit will be collected. History of medication and treatment to treat DED or to alleviate its symptoms, including artificial eye drops, will be collected without a time limit.
5) Medical/surgical history — Information on past medical and surgical history and concurrent diseases within 30 days prior to the screening visit will be collected. However, as for malignant tumors, information within 5 years prior to the screening visit will be collected. The target disease (DED) of the study will not be collected as a concurrent disease. For each medical history (past medical history, surgical history, and concomitant disease), information on progress at the time of screening will be collected. All ophthalmologic medical and surgical history will be collected without a time limit.
6) Basic disease information — Date of diagnosis of DED (duration of disease), status and duration of contact lenses use, DED related symptoms (sandiness/grittiness, dryness, light sensitivity, pain or soreness, blurred vision, etc.), etc.
7) Body measurement — Height and weight will be collected.
8) Vital signs/physical examination —
   (1) Vital signs: Body temperature, blood pressure (diastolic/systolic), and pulse rate
   (2) Physical examination: Appearance, skin, head/neck, heart, abdomen, urogenital system, limbs, musculoskeletal system, nervous system, lymph nodes, and others will be assessed by visual inspection, palpation, and interview.
9) Laboratory tests — Laboratory tests will be performed for the following parameters. Results obtained within 4 weeks prior to screening may be used, if available.
   - Hematology: WBC, RBC, Hemoglobin, Hematocrit, Platelet count, WBC diff count.
   - Blood biochemistry: Na, K, Ca, Cl, CPK, BUN, Creatinine, Uric acid, Total bilirubin, Albumin, Total protein, ALT, AST, γ-GTP, Alkaline phosphatase, Glucose, HbA1c (HbA1c will be tested at screening only.)
   - Serum hepatitis B and C: hepatitis B virus antigen (HBsAg), hepatitis C virus antibody (HCV Ab) (to be tested at screening only.)
   - Urinalysis: Protein, Glucose, Blood, Ketones
10) Pregnancy test — Urine hCG pregnancy test will be performed only in women of childbearing potential (If urine test is not available, this can be replaced with blood test. Menopause is defined as no menstruation for ≥ 1 year.).
11) ECG — 12-lead ECG will be performed. Results obtained within 4 weeks prior to screening may be used, if

	available.
12) TBUT	Time to appearance of the first dry spot will be measured.
13) Corneal staining	Corneal fluorescein staining score will be assessed by Oxford grading.
14) Conjunctival staining	Lissamine green conjunctival staining score will be assessed by Oxford grading.
15) IOP	In both eyes, IOP will consecutively be measured twice per eye using a noncontact tonometer.
16) Study eye selection	The study eye will be the eye with the worse corneal staining results, i.e., the higher corneal staining score. If both eyes have the same corneal staining score, the study eye will be the eye with the worse Schirmer's test results without anesthesia, i.e., the smaller tear production without anesthesia. If both eyes have the same Schirmer's test results without anesthesia, the right eye (OD) will be the study eye.
17) Rescue medication dispensing/retrieving	For the washout period and at baseline, Week 4, and Week 8, artificial eye drops (Refresh Plus™) will be dispensed for use as rescue medication, and any unused rescue medication will be retrieved at the next visit (Rescue medication dispensed at baseline will be retrieved at Week 4).
18) IP dispensing/retrieving	During the treatment period, IP will be prescribed for twice daily administration; One IP is to be used per time, one drop at a time for both eyes (2 IPs are to be used per day). At baseline, Week 4, and Week 8, IP will be prescribed, and any unused IP will be retrieved at the respective next visit (IP dispensed at baseline will be retrieved at Week 4).
19) Subject diary dispensing/retrieving	While participating in the study, subjects should complete the subject diary dispensed in this study. In the subject diary, treatment history of IP and rescue medication is to be recorded. The subject diary will be dispensed to subjects at baseline, Week 4, and Week 8 and retrieved at the respective next visit (The diary dispensed at baseline will be retrieved at Week 4) to check treatment history of IP and rescue medication.
20) AEs	All AEs occurring from after informed consent to study participation to the safety follow-up visit (Visit 7) will be collected.
21) EOT	EOT is defined as a visit (Visit 6) to be performed 12 weeks after baseline (Visit 2). At EOT, subjects should visit the institution after morning dose of IP (if necessary, rescue medication), and ophthalmologic examination will be performed at least 2 hours after that.
22) Safety follow up	Subjects will visit the institution 2 weeks after EOT for safety follow-ups and undergo ophthalmologic examination and laboratory tests (if necessary).<br>If possible, any unresolved AEs at the safety follow-up visit (Visit 7) will be followed up until resolution by telephone or on-site visit.
23) UV	During unscheduled visits, tests and assessments may be performed if necessary at the discretion of the investigator.

3. Introduction

3.1. Background and Rationale

3.1.1. Disease Information

Dry eye disease (DED) is a multifactorial disease of the ocular surface with a loss of homeostasis of the tear film and ocular symptoms, in which tear film instability and hyperosmolarity, ocular surface inflammation and damage, and neurosensory abnormalities play etiological roles.[1, 2]. Results from a global epidemiological study showed that the prevalence of DED had a wide range of 5 to 50%.[11] In the United States (US), approximately 6.8% of adult population (approximately 1,640 adults) were diagnosed with DED.[3]

In South Korea, the number of patients diagnosed with DED was up by approximately 470,000 patients (26.7%) in 5 years from 1,750,000 patients in 2009 to 2,220,000 patients in 2013[1], and to 2,540,000 patients in 2019[4], according to Health Insurance Review & Assessment Service. Mean annual increase rate was 6.1%.[5] This is approximately 9 folds the mean annual increase rate of patients with digestive system disorders (0.7% in 5 years from 2011 to 2015), a disease accounting for the largest percentage of 78.1% of all patients diagnosed in South Korea.[5]

As a multifactorial disease, DED may induce eye discomfort and visual impairment due to tear film hyperosmolarity, ocular surface inflammation, and tear film instability[6, 7, 8] and may be accompanied by subacute ocular surface inflammation.[9] Depending on the severity of the disease, patients with DED may experience not only eye irritation and discomfort but also vision abnormalities. Severe DED is characterized by clinical signs including serious corneal involvement along with chronic severe and/or completely disabling symptoms.[10]

By etiology, DED is generally classified into aqueous deficient DED and evaporative DED.[6, 7, 9, 11] According to the International Dry Eye Workshop (DEWS) II classification, there is also a combined form of aqueous deficient and evaporative DED, with evaporative DED playing a predominant part.[2] Aqueous deficient DED is further classified into Sjogren's syndrome DED, an autoimmune disease involving lacrimal glands and salivary glands; and non-Sjogren's syndrome DED that rules out the systemic autoimmune disease. Non-Sjogren's syndrome DED is most commonly caused by aging, and other causes of non-Sjogren's syndrome DED include primary or secondary lacrimal gland deficiency, lacrimal duct occlusion, and decreased reflex lacrimation etc. Evaporative DED occurs as a result of excessive loss of water on the ocular surface due to intrinsic or extrinsic factors despite normal lacrimal gland secretion. The most common cause of evaporative DED is meibomian gland dysfunction, which causes not only meibomian gland inflammation, dystrophy, and occlusion but also lipid layer disorder or decrease, resulting in tear instability.[12] In addition, watching digital screens or performing tasks requiring concentration for a long time may generally reduce eye blinking, which in turn, leads to water evaporation from the ocular surface, resulting in DED. As described earlier, DED is a multifactorial disease involving various risk factors, and use of visual display terminals (VDT) including televisions, computers, and mobile phone screens, is closely associated with DED.[13, 14] A recent increase in the continuous and prolonged use of VDT for professional, educational, and recreational purposes[15] coupled with increased home-based working or teleworking and contact-free on-line educations as well as decreased outdoor activities due to the Coronavirus Disease-19 (COVID-19) pandemic since 2019 are thought to have contributed to increased exposure to VDT. This is supported by a significant increase in VDT use hours and a significant increase in eye strain and dry eye symptoms upon switching to home-based working or teleworking since the COVID-19 pandemic, suggesting the association with VDT use hours.[16]

Treatment of DED based on these two etiologies (aqueous deficient, evaporative) has focused on maintaining a certain level of tear either by supplementing water to the eyes or by temporarily or permanently blocking lacrimal passages.[11] However, it is assumed that the pathogenic mechanism is a stress put on the ocular surface, i.e., environmental factors, infection, intrinsic stress, and antigens, etc., and a vicious circle of the ocular surface damage caused by the earlier mentioned factors leading to inflammation does actually occur.[9] In addition, previous studies defined DED as one of the inflammatory diseases.[11] Indeed, ocular surface inflammation has recently been recognized as a major inducer of DED and therefore has become a treatment target.[17] For this reason, anti-inflammatory drugs are used for the treatment of DED.[11]

3.1.2. Treatment Methods

Mild DED without severe symptoms is usually treated with eye drops such as artificial eye drops. However, DED with more serious and prolonged symptoms are treated with drug therapy besides artificial eye drops, and if necessary, procedures are attempted. Some examples of treating DED by controlling an inducer of the disease are as follows. If a drug

that is used for health issues induces DED, therapy can be switched to another drug that does not induce DED, or a plastic surgery may improve DED if the patient has a structural problem in the eyelids.
Besides treatment of underlying disease or removal of external harmful factors, treatment of DED may vary depending on the severity of the disease as it aims to improve signs and symptoms of the disease. DED Patients with mild to moderate symptoms can generally be treated with long term use of lubricants/artificial eye drops.[18] Drugs used for the treatment of DED are largely divided into drugs to reduce eyelid inflammation, eye drops to control cornea inflammation, eye inserts that work like artificial tears, tear-stimulating drugs, and eye drops made from patient's own blood. Procedures such as lacrimal punctal occlusion can also be performed.
The DEWS guidelines recommend that patients with moderate to severe DED should start treatment with topical anti-inflammatory drugs such as corticosteroids and cyclosporine.[19] However, prolonged use of corticosteroid eye drops is not recommended due to increased risk of side effects such as ocular hypertension, ocular infection, and cataract.[8]
Cyclosporine is the active ingredient of Restasis® 0.05%, a drug product that serves as the comparator in this study. It is a lipophilic cyclic polypeptide that has long been used as a systemic immunosuppressant to prevent graft rejection following organ/tissue transplantation and for the treatment of various immune diseases.[20] Cyclosporine is also characterized by its anti-inflammatory effects; It is known to prevent cell-mediated reactions, inhibit the production and/or release of proinflammatory cytokines including interleukin 2(IL-2), and activate the release of anti-inflammatory cytokines. With this mechanism of action, cyclosporine is known to be also effective when it is applied to the eyes as eye drops; The compound is found to effectively restore the damaged function of lacrimal glands by inhibiting the production of inflammatory cytokines and by inhibiting T-cell function. Based on these findings, Restasis®, a cyclosporine ophthalmic emulsion, was approved by the U.S. Food and Drug Administration (FDA) in 1983 and subsequently in South Korea in 2005 to increase tear production in patients with suppressed tear production and has since been widely used to date for the treatment of DED.[11, 21]

3.1.3. Rationale for Study Conduct

Cyclosporine is poorly soluble in water due to strong intramolecular forces, making it relatively difficult to interact with water molecules.[11] To improve the poor solubility of cyclosporine, SCAI Therapeutics developed SCAI-001 using Molecular Associated Innovative Substance (MOASIS™), thereby increasing solubility only with a small amount of surfactant. With this improvement, SCAI-001's cyclosporine has greater solubility and permeability. This study aims to assess the difference in the effects on DED symptoms between SCAI-001's cyclosporine with a smaller strength of cyclosporine (0.01% and 0.02%) than conventional drugs and Restasis® 0.05%, a conventional cyclosporine product.

3.2. Nonclinical Study Results

3.2.1. Efficacy Study Results

The efficacy of SCAI-001 in DED was assessed in 3 efficacy studies. The results are summarized below.

<u>Efficacy assessment for SCAI-001 in dry eye mouse models (Study no. 2019-T2B-020)</u>
A study was performed to assess the preventive and therapeutic effect of SCAI-001(0.05%), a candidate compound for the treatment of dry eyes, after 10 days of drying stress given to dry eye mouse models. The dry eye mouse models were established by subcutaneously injecting scopolamine hydrobromide into the thigh for 10 days, 4 times a day. The test groups consisted of SCAI-001 as test article and Restasis® 0.05%, Cyporine N, T-sporin, Eyeporin, and Ikervis as positive controls; With the exception of Ikervis (0.1%), all study materials contained cyclosporine 0.05%. The study materials were administered to both eyes for 10 days, twice daily, 5 $\mu\ell$ at a time. For clinical analysis, tear production, ocular surface flexuosity, and corneal fluorescein staining were performed on Days 3, 5, 7, and 10 of treatment. For histological analysis, mice were sacrificed to extract eyes and lacrimal glands on Day 10 of treatment in all treatment groups. The extracted eyes were examined for corneal epithelial cell detachment, conjunctival goblet cell distributions, and conjunctival mucin distributions, and the lacrimal glands were examined for the expression of inflammatory factors (TNF-α, ICAM-1, VCAM-1, MMP-2, MMP-9) by immunohistochemical staining.
Results from the measurement of tear production showed that tear production which was decreased by drying stress increased 2.8 folds with SCAI-001, which was comparable to Restasis® 0.05%. Results from the analysis of ocular surface

flexuosity showed that ocular surface flexuosity which was increased by drying stress improved by 32.6% with SCAI-001, which was a statistically significant decrease in comparison with all positive controls. Results from corneal fluorescein staining indicated that corneal fluorescein staining score which was increased by drying stress decreased by 37.9% with SCAI-001, which was a significantly greater decrease in comparison with all positive controls (Restasis® 0.05%, Cyporine N, T-sporin, Eyeporin, Ikervis). Corneal epithelial cell detachment which was increased by drying stress was suppressed by 81.3% with SCAI-001. Conjunctival goblet cell and mucin distributions which were decreased by drying stress significantly recovered with all positive controls, which, however, did not recover in the SCAI-001 group. Results from the analysis of expression of inflammatory factors closely associated with dry eyes indicated that the expression of all inflammatory factors which was increased by drying stress decreased to a similar level across the groups (test group and positive control groups).

SCAI-001 0.05% is considered to be an appropriate anti-inflammatory treatment for dry eyes based on the improvement in ocular surface flexuosity and corneal fluorescein staining score and the inhibitory effect on corneal epithelial cell detachment.

Efficacy assessment for SCAI-001 by particle size in dry eye mouse models (Study no. 2020-T2B-010)

A study was performed to assess the efficacy of SCAI-001(0.05%), a candidate compound for the treatment of dry eyes, as A(0.001%), B(0.015%), and C(0.05%) according to concentration, after 10 days of drying stress given to dry eye mouse models. The dry eye mouse models were established by subcutaneously injecting scopolamine hydrobromide into the thigh 4 times a day. Vehicle as a negative control, SCAI-001 A(0.001%), B(0.015%), and C(0.05%) as test articles, and Restasis® 0.05% as a positive control were administered to both eyes for 10 days, twice daily, 5㎕ at a time. After 10 days of drying stress, tear production, ocular surface flexuosity, and corneal fluorescein staining were performed on Days 3, 5, 7, and 10 of treatment for clinical analysis. For histological analysis, mice were sacrificed to extract eyes and lacrimal glands on Day 10 of treatment in all treatment groups. The extracted eyes were examined for corneal epithelial cell detachment, conjunctival goblet cell distributions, and conjunctival mucin distributions, and the lacrimal glands were examined for the expression of inflammatory factors (TNF-α, ICAM-1, VCAM-1, MMP-2, MMP-9) by immunohistochemical staining.

Across all test items, the efficacy of SCAI-001 A(0.001%), B(0.015%), and C(0.05%) was similar or superior to that of Restasis® 0.05%. In the tear production test, tear production significantly increased 1.9, 6.3, 4.6, 5.0, and 4.0 folds in the vehicle, SCAI-001 A(0.001%), B(0.015%), C(0.05%), and Restasis® 0.05% groups, respectively; SCAI-001 C(0.05%) showed the greatest effect while the results of A(0.001%) and B(0.015%) were similar to that of Restasis® 0.05%. In the ocular surface flexuosity and corneal fluorescein staining tests, Restasis® 0.05% indicated no improvement while SCAI-001 A(0.001%), B(0.015%), and C(0.05%) significantly improved in comparison with vehicle; The effect of SCAI-001 B(0.015%) and C(0.05%) was greater than that of SCAI-001 A(0.001%). Based on histological findings, SCAI-001 A(0.001%) and B(0.015%) were superior to SCAI-001 C(0.05%) in terms of corneal epithelial cell detachment and conjunctival goblet cell and mucin distributions.

SCAI-001 A(0.001%), B(0.015%), and C(0.05%) were similar or superior to Restasis® 0.05% based on anti-inflammatory effects as evidenced by increased tear production, improvement in ocular surface damage, and recovery of conjunctival goblet cell and mucin distributions. Of the three candidate compounds, SCAI-001 B(0.015%) is considered to be the most appropriate treatment for dry eyes.

Efficacy assessment for SCAI-001 by formulation in dry eye mouse models (Study no. 2021-T2B-002)

A study was performed to assess the efficacy of SCAI-001 B-05(0.005%), SCAI-001 B-10(0.01%), and SCAI-001 B-20(0.02%) in comparison with Restasis® 0.05% in DED animal models induced by giving drying stress for 10 days.

The dry eye animal models were established by subcutaneously injecting scopolamine hydrobromide (0.5 mg/0.2 ml) into the thigh 4 times a day; Drying stress was maintained for 10 days with ≤ 40% humidity feeding environment. Vehicle as a negative control, SCAI-001 B-05(0.005%), B-10(0.01%), and B-20(0.02%) as test articles, and Restasis® 0.05% as a positive control were administered to both eyes for 10 days, twice daily, 5㎕ at a time. After 10 days of drying stress, tear production, ocular surface flexuosity, and corneal fluorescein staining were performed for clinical analysis on Days 3, 5, 7, and 10 of treatment. For histological analysis, mice were sacrificed to extract eyes and lacrimal glands on Day 10 of treatment in all treatment groups. The extracted eyes were examined for corneal epithelial cell detachment, conjunctival goblet cell and conjunctival mucin distributions, and the lacrimal glands were examined for the expression of inflammatory

factors (TNF-α, ICAM-1, VCAM-1, MMP-2, MMP-9) by immunohistochemical staining.
Results demonstrated the efficacy of SCAI-001 B-05, B-10, and B-20 by SCAI-001 concentration in dry eye mouse models. In the tear production test, tear production statistically significantly increased 3.7, 2.8, 4.9, 4.3, and 3.7 folds in the vehicle, SCAI-001 B-05, B-10, B-20, and Restasis® 0.05% groups, respectively, on Day 10 of treatment. In all SCAI-001 groups, tear production was similar to that in the Restasis® 0.05% group, and in particular, the SCAI-001 B-10 and B-20 groups indicated a statistically significant increase in tear production in comparison with the vehicle group. SCAI-001 B-05, B-10, and B-20 indicated improvement in ocular surface flexuosity score by 27.3%(B-05), 49.1%(B-10), and 75.4%(B-20), respectively, and improvement in corneal fluorescein staining score by 41.3%(B-05), 49.5%(B-10), and 63.6%(B-20), respectively, in comparison with vehicle. All of these three candidate compounds had statistically significant improvement in comparison with vehicle and Restasis® 0.05%, with SCAI-001 B-20(0.02%) showing the greatest decreasing effects. Histological findings indicated that the vehicle, SCAI-001 B-05, B-10, and B-20 groups suppressed corneal epithelial cell detachment by 10%, 15%, 30%, and 45%, respectively, in comparison with the drying stress group; There was no significant difference between the SCAI-001 candidate compounds. While the Restasis® 0.05% group did not suppress corneal epithelial cell detachment, the SCAI-001 B-20(0.02%) group showed a statistically significant difference in comparison with the Restasis® 0.05% group. In terms of conjunctival goblet cell and mucin distributions, SCAI-001 B-05 and B-10 were as highly effective as Restasis® 0.05%. Across all concentrations of B-05, B-10, and B-20, SCAI-001 had anti-inflammatory effects on lacrimal glands, which was comparable to that of Restasis® 0.05%.
Of the three concentrations with comparable anti-inflammatory effects, SCAI-001 B-20 is thought to be appropriate for the treatment of dry eyes for greater tear production and improvement in ocular surface damage while SCAI-001 B-05 and SCAI-001 B-10 are thought to be appropriate concentrations for the treatment of dry eyes for recovery of conjunctival goblet cell and mucin distributions.

3.2.2. Pharmacokinetic Study Results

A summary of the pharmacokinetics (PK) of cyclosporine, the active ingredient of SCAI-001, is provided below.

A PK study of SCAI-001 eye drops after administration to the eyes (study no. BBPP21058_A001)
The PK distributions of cyclosporine in the eyes and in blood were analyzed after single or repeat doses of SCAI-001 eye drops 0.01% and 0.02% as test articles and Restasis® 0.05% as a positive control in New Zealand white (NZW) rabbit models. In the single-dose study, rabbits were administered a single dose of SCAI-001 and Restasis® 0.05%. In the repeat-dose study, rabbits were administered SCAI-001 and Restasis® 0.05% for 7 days, twice daily, at an interval of 12 hours. The study materials were administered to both eyes, 50 ㎕ at a time. Cyclosporine concentrations were analyzed in blood and 9 ocular tissue samples (cornea, conjunctiva, aqueous humor, crystalline lens, iris, vitreous humor, sclera, choroid, and retina) collected at each time point.
For cyclosporine analysis, whole blood, tear, and ocular tissue samples were collected from 2 rabbits per sampling time point. Sampling time points were 0.5, 1, 3, 6, 12, 24, 48, and 72 hours post-dose in the single-dose study; and 1 hour before the first dose of Day 7 and 1, 6, 24, and 72 hours after the second dose of Day 7 in the repeat-dose study.
Results are as follows. In the single-dose study, both SCAI-001 and Restasis® 0.05% had the highest exposure in cornea and conjunctiva based on AUC_{last}. Upon dose normalization based on the strength of cyclosporine in the eye drops, the exposure in cornea and conjunctiva (based on AUC_{last}) was 5.47 and 5.21 folds, respectively, with SCAI-001 0.01% and 3.98 and 3.44 folds, respectively, with SCAI-001 0.02% versus Restasis® 0.05%.
In the 7-day repeat-dose study, both SCAI-001 and Restasis® 0.05% had the highest exposure in cornea based on AUC_{last}. Upon dose normalization based on the strength of cyclosporine in the eye drops, the exposure in cornea and conjunctiva (based on AUC_{last}) was 9.13 and 4.06 folds, respectively, with SCAI-001 0.01% and 5.00 and 2.69 folds, respectively, with SCAI-001 0.02% versus Restasis® 0.05%.
After SCAI-001 dosing, the exposure of cyclosporine in cornea and conjunctiva accounted for 96.52~98.05% in the single-dose study and 94.73~96.01% in the repeat-dose study of the total amount of cyclosporine detected in all tissues.
In the whole blood samples, C_{max} was reached in 1 hour after dosing in all study groups, and the exposure in the majority of samples was below the low limit of quantitation (LLOQ) (<0.34 ng/mL).

PK of Cequa®

Cequa® 0.09% is a nanomicelle formulation of cyclosporine, the active ingredient of SCAI-001. A summary of the PK of Cequa® 0.09% based on the FDA Pharmacological review (Sun Pharma) is provided below.

Ocular tissue distributions were compared after single or 7-day repeat doses of Cequa® 0.09%(OTX101) and Restasis® 0.05% in female NZW rabbits. In the single- and repeat-dose studies, one drop (35㎕/eye) of the study material was administered to each eye of the rabbits. While only OTX101 0.05% was compared with Restasis® 0.05% in the single-dose study, OTX101 0.01%, 0.05%, and 0.1% were administered to NZW rabbits for 7 days, 4 times a day (every 2 hours), in the repeat-dose study. Given that the approved clinical dose of Restasis® 0.05% is 1 drop per eye, twice daily, the single and repeat doses in this PK study are equivalent to 1/2 and 2 folds, respectively, of the clinical dose.

Animals were observed for body weight, mortalities, and clinical symptoms. For cyclosporine analysis, whole blood, tear, and ocular tissue samples were collected from 2 rabbits per time point. Sampling time points were 0.25, 0.5, 1, 2, 4, 8, 12, 24, 28, and 72 hours post-dose in the single-dose study; and on Day 4 (before the first dose of the day) and Day 7 (before the first dose, 2 hours after the third dose [before the fourth dose], and 0.25, 0.5, 1, 2, 4, 8, and 18 hours after the fourth dose of the day) in the repeat-dose study.
After single or repeat doses, there were no mortalities, clinical symptoms, or weight change. With the exception of upper eye lid, tear, and vitreous humor, overall ocular exposure of cyclosporine (based on AUC_{last}) after a single dose of OTX101 0.05% was 1.2~1.6 folds greater than that after Restasis® 0.05% dosing. All OTX101 concentrations in vitreous humor samples were below the LLOQ. The exposure was in the decreasing order of cornea > upper eye lid > tear > third eyelid > superior bulbar conjunctiva> sclera > iris-ciliary body > choroid/retina and lacrimal gland > aqueous humor > crystalline lens >whole blood> vitreous body; The order was similar to that after Restasis® 0.05% dosing.
After repeat doses of OTX101 0.01%, 0.05%, and 0.1%, cyclosporine exposure (based on AUC_{last}) increased in a dose dependent manner in ocular tissues and whole blood. With the exception of lacrimal gland, upper eye lid, and tear, cyclosporine concentrations in ocular tissues and whole blood after OTX101 dosing were higher than those observed at the same doses of Restasis® 0.05%. Across the ocular tissues, the exposure ($AUC_{(0-T)}$) after repeat doses of OTX101 0.05% increased by 1.40 (lacrimal gland) to 11.68 folds (crystalline lens) between Day 1 and Day 7, suggesting cumulation upon repeat doses; A generally similar tendency was observed with Restasis® 0.05%. Results from a comparison with the same cyclosporine concentrations and dosage (0.05%, 4 times/day) in a 7-day comparative PK study in NZW rabbits indicated that cyclosporine levels in the anterior ocular tissues (e.g., vitreous humor, crystalline lens, iris-ciliary body) were 3~5 folds greater than those with Restasis® 0.05%.

3.3. Previous Clinical Trial Results

To date, the investigational product (IP) has not been studied in humans. This is the first-in-human trial.

3.4. Rationale for Dose Determination

The efficacy of SCAI-001 B-05(0.005%), SCAI-001 B-10(0.01%), and SCAI-001 B-20(0.02%) was evaluated in comparison with Restasis® 0.05% in DED animal models developed by applying drying stress to ≥ 12 week old mice (NOD.B10-$H2^b$ mouse).
Results demonstrated the efficacy of SCAI-001 according to concentrations in the dry eye mouse models. On Day 10 after removal of drying stress, tear production statistically significantly increased by 4.9 and 4.3 folds with SCAI-001 B-10 and B-20, respectively, which were greater than a 3.7-fold increase with Restasis® 0.05%. Ocular surface flexuosity and corneal fluorescein staining score indicated dose dependent decreases with SCAI-001 B-05, B-10, and B-20, all of which were statistically significant decreases compared with the Restasis® 0.05% group. To sum up, SCAI-001 B-05, B-10, and B-20 showed favorable efficacy profiles and equivalent or greater therapeutic effects than Restasis® based on the results associated with clinical endpoints. Histological findings indicated that the greatest inhibitory effect on corneal epithelial cell detachment was observed with SCAI-001 B-20; The inhibitory effect was statistically significant in comparison with Restasis®. In terms of conjunctival goblet cell and mucin distributions, SCAI-001 B-05 and B-10 were as effective as

Restasis®. In terms of anti-inflammatory effects in lacrimal glands, all SCAI-001 concentrations of B-05, B-10, and B-20 were as effective as Restasis®.

Based on these results, it was determined that SCAI-001 B-10(0.01%) and B-20(0.02%) were appropriate for the treatment of DED for greater tear production and improvement in ocular surface damage while SCAI-001 B-05(0.005%) and SCAI-001 B-10(0.01%) were appropriate for the treatment of DED for recovery of conjunctival goblet cell and mucin distributions.

Results from the analysis of PK distribution of cyclosporine in the eyes and blood after single or repeat doses of SACI-001 0.01% and 0.02% versus the comparator, Restasis® 0.05%, in NZW rabbit models indicated that, after *in vivo* administration into the eyes, SCAI-001 0.01% was distributed at higher concentrations than Restasis® 0.05% in cornea, conjunctiva, iris, and choroid, and SCAI-001 0.02% was distributed at higher concentrations than Restasis® 0.05% in all tissues. In whole blood samples, C_{max} was reached in 1 hour after dosing in all study groups, and the exposure in the majority of samples was below the LLOQ.

These results demonstrate cyclosporine exposure in cornea and conjunctiva with both SCAI-001 eye drops 0.01% and 0.02%, despite the low doses compared with Restasis® 0.05%, suggesting that, despite at lower doses than Restasis® 0.05%, SCAI-001 is delivered to the ocular tissues more efficiently than Restasis® 0.05%. SCAI-001 exposure in cornea and conjunctiva accounted for 96.52~98.05% in the single-dose study and 94.73~96.01% in the repeat-dose study of the total amount of cyclosporine detected in all tissues. These findings suggest that the exposure in non-target tissues is smaller than that in cornea and conjunctiva, the target tissues of the drug. In addition, the amount of drug that is out of the target region, i.e., ocular tissues, and enters bloodstream is minimal - ≤ 0.02% of the administered single or repeat doses – suggesting little impact by off-target systemic exposure.

Based on these results, the present study is designed to evaluate the therapeutic effect of SCAI-001 eye drops 0.01% and 0.02% in comparison with Restasis® 0.05% in patients with DED.

4. Study Objectives

The objectives of the study are to demonstrate the efficacy and safety of SCAI-001 eye drops (cyclosporine 0.01% or 0.02%) based on the evaluation of efficacy and safety in comparison with Restasis® (cyclosporine 0.05%), the active comparator, and to explore the appropriate doses of SCAI-001 eye drops in patients with DED.

5. Study Population

5.1. Planned Sample Size

This study will enroll a total of 114 subjects (38 subjects per group) with a 20% drop-out rate taken into consideration.

5.2. Study Indication

DED

5.3. Inclusion Criteria

5.3.1. Screening Inclusion Criteria

To be enrolled in this study, subjects must meet all of the following inclusion criteria at the screening visit.

1. Male and female adult at the age of ≥ 19 years old
2. Individual with dry eye symptoms (sandiness/grittiness, dryness, light sensitivity, pain or soreness, blurred vision, etc.)
3. Individual whose either or both eyes meet all of the following criteria at screening:
   1) Inflammation in cornea and conjunctiva with corneal staining score - Oxford grading ≥ 2
   2) Schirmer's test without anesthesia ≤ 10 mm/5 min (If Schirmer's test without anesthesia is 0 mm/5 min, nasal stimulation Schirmer's test in the same eye must be ≥ 3 mm/5 min.)
   3) Tear film break-up time (TBUT) ≤ 10 seconds
4. Individual with best corrected visual acuity ≥ 0.2 in both eyes
5. Individual who is able to understand the study and voluntarily provides written informed consent

5.3.2. Randomization Inclusion Criteria

To be enrolled in this study, subjects must meet all of the following inclusion criteria at the baseline visit.

1. Individual with dry eye symptoms (sandiness/grittiness, dryness, light sensitivity, pain or soreness, blurred vision, etc.)
2. Individual whose eye that has met the following criteria at screening meets all of the following criteria (In the case that both eyes have met all of the criteria at screening, the subject will be eligible if at least either eye meets all of the following criteria at randomization.):
   1) Inflammation in cornea and conjunctiva with corneal staining score - Oxford grading ≥ 2
   2) Schirmer's test without anesthesia ≤ 10 mm/5 min (If Schirmer's test without anesthesia is 0 mm/5 min, nasal stimulation Schirmer's test in the same eye must be ≥ 3 mm/5 min.)
   3) TBUT ≤ 10 seconds
3. Individual with best corrected visual acuity ≥ 0.2 in both eyes

5.4. Exclusion Criteria

Individuals who meet any of the following exclusion criteria will be excluded from study enrollment.

1. Individual with clinically significant ophthalmologic diseases (corneal surface disease, abnormal corneal sensitivity,

abnormal dacryorrhea, etc.) not attributed to DED

2. Individual who has a history of treatment with systemic steroids and immunosuppressants (azathioprine, tacrolimus, cyclosporine, mycophenolate mofetil, etc.) within 90 days prior to screening
3. Individual who has a history of treatment with cyclosporine eye drops within 6 weeks prior to screening
4. Individual who has a history of treatment with any anti-inflammatory drugs for DED[a)] other than cyclosporine eye drops within 30 days prior to screening
   [a)] Anti-inflammatory drugs for DED: Steroidal or non-steroidal anti-inflammatory eye drops, autologous serum eye drops, mast cell stabilizers (including eye drops), etc.
5. Individual who has a history of treatment with any other drugs that may affect DED [b)] within 30 days prior to screening. However, those who have been treated with such drugs for ≥ 30 days prior to screening with the same dosage and administration and will maintain the treatment with the same dosage and administration throughout the study will be allowed to participate in the study.
   [b)] Other drugs that may affect DED: Oral contraceptives, anticholinergics, tricyclic antidepressants, antihistamines, hypnotics, diuretics, antimuscarinics, beta blockers, retinoids, aspirin, or non-steroidal anti-inflammatory drugs (NSAIDs) (For non-steroidal anti-inflammatory eye drops, see exclusion criterion 4.), etc.
6. Individual who has worn contact lenses within 72 hours prior to screening or who needs to wear them during the study
7. Individual who has a history of intraocular surgery within 12 months prior to screening
8. Individual who has a history of intraocular procedures, such as punctal plug, lacrimal punctal occlusion, cauterization of the punctum, ophthalmologic intense pulsed light (IPL) laser, etc., within 90 days prior to screening
9. Individual who has a history of vision correction surgery, such as LASIK, LASEK, etc., within 12 months prior to screening
10. Individual with symptoms of active eye infection such as active allergy, anterior uveitis, Stevens-Johnson syndrome, etc.
11. Patient with autoimmune diseases such as Sjogren's syndrome, etc.
12. Individual who has a history of corneal transplant or neurotrophic keratitis
13. Individual who has a history of hepatitis B or C
14. Individual who has a history of malignant tumors within 5 years prior to screening (However, those with adequately treated ['interventional procedure' or 'considered inactive based on the judgment of the investigator'] basal or squamous epithelial cell skin cancer or cervical intraepithelial neoplasia will be allowed to participate in the study.)
15. Uncontrolled hypertension (> 160/100 mmHg) at the screening or baseline visit or insulin dependent or uncontrolled diabetes mellitus (HbA1c > 8% based on the screening test) at screening
16. Individual with intraocular pressure (IOP) > 21 mmHg in either or both eyes or who is diagnosed with glaucoma
17. Individual who is expected to require an intraocular procedure or surgery during the study, based on the judgment of the investigator
18. Individual with any of the following laboratory test values:
    1) Creatinine level ≥ 2 x site-specific upper limit of normal (ULN)
    2) ALT or AST level 2 x site-specific ULN
19. Individual with hypersensitivity reactions to any of the components of IP
20. Individual with mental disorders
21. Pregnant or lactating woman
22. Woman who plans to become pregnant or who has a positive pregnancy test result at screening, or woman of childbearing potential or man who does not agree to use at least one of the effective, medically acceptable methods of birth control* from screening to 14 days after the end of IP treatment (During this period, egg and sperm donation will be prohibited for female and male subjects, respectively.)
    * Medically acceptable methods of birth control: Intrauterine device (loop, Mirena, etc.), double barrier methods in which both man and woman use contraceptives (condom/femidom or diaphragm or cervical cap), non-oral contraceptives (injection, contraceptive ring, patch, etc.), complete abstinence.
    Oral contraceptives and periodic abstinence (e.g., basal body temperature method, natural cycles method, etc.) alone will not be allowed.

However, women who had menopause (no menstruation for ≥ 1 year) prior to study participation or surgically sterilized (vasectomy, bilateral tubal ligation, etc.) women or men will be allowed to participate in the study without consent to birth control.

23. Individual who has a history of drug or alcohol abuse
24. Individual who participated in another clinical trial within 30 days prior to screening
25. Individual who is ineligible for study participation based on the judgment of the investigator

5.5. Premature Withdrawal

5.5.1. Definition

Subjects who are assigned a randomization number but cannot participate for the entire study period for any reason will be classified as 'withdrawals'. If a subject wants to stop the study or withdraws his or her informed consent, or if the investigator determines that a subject should be withdrawn, the subject can be withdrawn at any time. If the study is terminated or the subject is withdrawn, study results obtained up to that point may be reviewed for evaluable items at the time of final assessment. For all subjects who participate in the study, status of study completion will be documented; If visits or follow-ups are discontinued, reasons will be documented.

5.5.2. Criteria for Premature Withdrawal

1) Informed consent is not obtained prior to study procedures
2) Violation of the inclusion/exclusion criteria is identified during the study
3) Subject or subject's legally acceptable representative withdraws informed consent to study participation
4) Blind is broken during the study
5) Subject experiences acute reactions (allergy, hypersensitivity reactions, etc.) to IP
6) Subject becomes pregnant during the study
7) Continued study participation is considered inappropriate due to serious adverse events (SAEs) or serious adverse drug reactions (SADRs) which occurred during the study
8) Lost to follow-up before the end of treatment (EOT) visit
9) Protocol violation which makes continued study participation inappropriate
10) Subject's study participation is considered inappropriate for other reasons based on the judgment of the investigator

If a subject stops participating in the study due to safety reasons such as adverse events (AEs) or worsening of health, the principal investigator (PI) or the sub-investigator will handle the case as appropriate, document in the subject's case report form (CRF) date of onset of symptoms, clinical symptoms, test findings, treatment, and course, and follow up the subject until recovery of the symptoms, if possible.

To prevent any AEs from being uncollected, subjects who refuse to visit should directly be contacted using all methods available such as telephone, letter (or email), or direct visit, and these attempts will be documented.
For subjects who are lost to follow-up, attempts will be made to contact them as much as possible in order to minimize loss to follow-up, and these attempts will be documented.

5.5.3. Protocol Violation

If a subject is found to have violated the protocol during the study, the investigator should inform SCAI Therapeutics of the violation as promptly as possible, and the sponsor will determine the seriousness of the case. In addition, the subject's continued study participation should be determined by the sponsor and the investigator. If a subject is withdrawn from the study due to a protocol violation, this should be documented in the CRF. Major protocol violations include, but are not limited to:

1) Violation of the inclusion and exclusion criteria that may affect the evaluation of efficacy and safety
2) Treatment with concomitant drugs or therapies during the study period
3) Informed consent not obtained prior to study procedures
4) Blind is broken without complying with protocol-defined procedures
5) Criteria for premature withdrawal are met, but participation is not stopped
6) IP is administered at a dose that is inappropriate for the evaluation of efficacy and safety
7) Other protocol violations that are considered major violations

5.5.4. Handling of Premature Withdrawals and Protocol Violations

Any of these situations should be documented in the subject's CRF and source documents. For those who are withdrawn, reasons for withdrawals will be documented in the study completion page of the CRF. Subjects who are withdrawn may be included in the analysis for test results obtained previously.

Subjects who are withdrawn should visit the institution as soon as possible after the withdrawal is determined and should undergo procedures for the EOT visit (Visit 6). If the subject has unresolved AEs at this point, safety follow-up will be performed until 2 weeks from the last IP dose or until resolution, if possible.

If a subject is withdrawn due to an AE, the subject will be followed up, if possible, until the AE recovers to the state prior to IP dosing or to baseline value, or until the AE is considered normalized by the PI.

Subjects who stop participating in the study or who are withdrawn from the study after randomization will not be replaced, regardless of the reason.

6. Study Design

6.1. Study Methods

This is a multicenter, active control, parallel group, randomized, double-blind, Phase II clinical trial to evaluate the efficacy and safety of SCAI-001 eye drops in patients with DED.

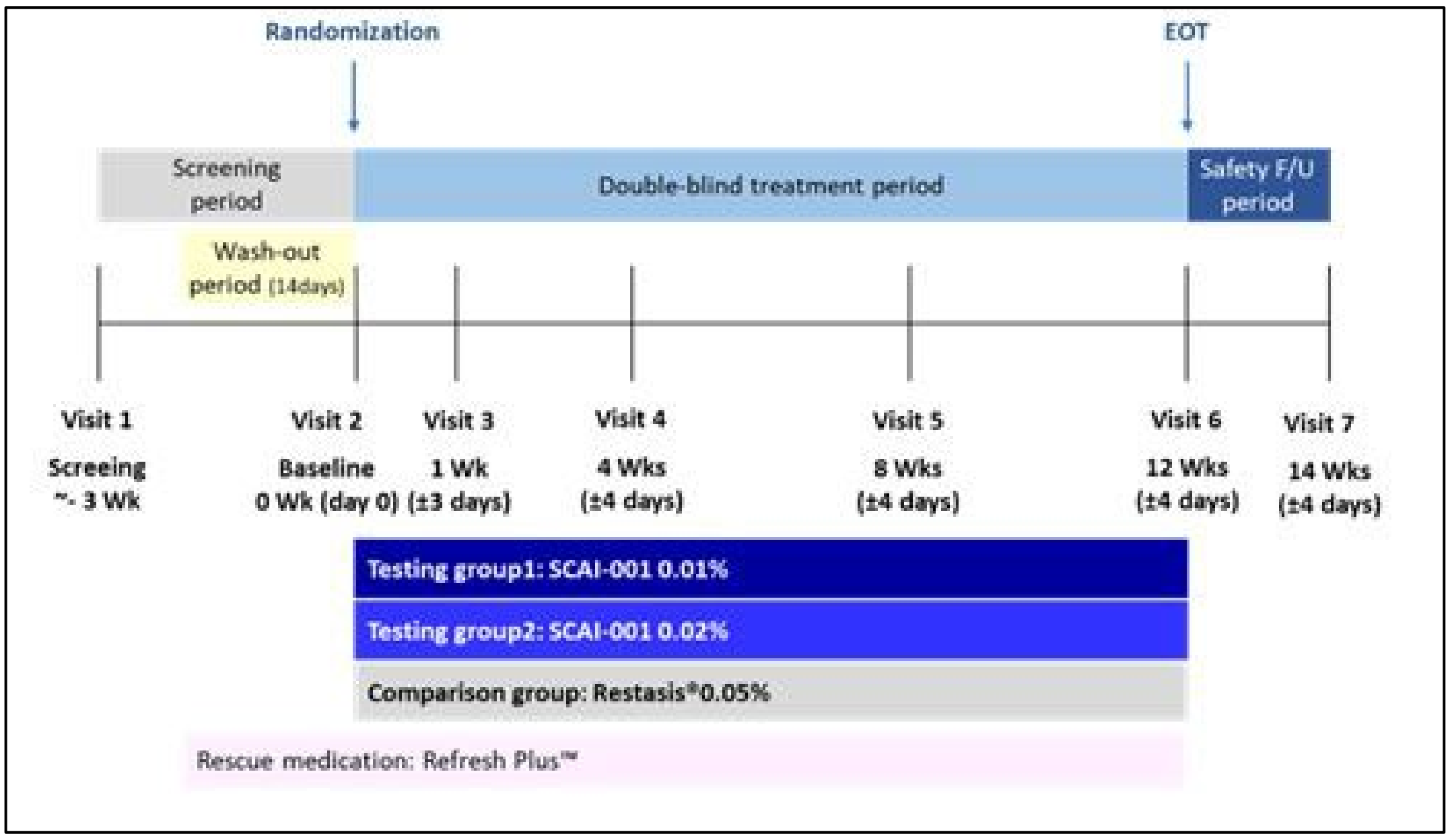

Figure 1. Study Scheme

Screening period (Visit 1, ~-1 week) and wash-out period (~-2 weeks)

Subjects who voluntarily sign the informed consent form (ICF) at the screening visit (Visit 1) will undergo screening tests. After the screening tests, those who are determined eligible based on the inclusion and exclusion criteria will undergo a wash-out period during which DED treatment the subject has been on within 1 week will be discontinued for at least 2 weeks. If there is no ongoing DED treatment at the start of study participation, the wash-out period may be skipped. During the wash-out period, the existing DED treatment will be stopped, and if necessary, rescue medication (Refresh Plus™) provided in the study may be administered. However, rescue medication is not to be administered from the day before the baseline visit (randomization). Rescue medication is to be administered 1~2 drops at a time, no more than 3 times a day, if possible. When rescue medication is used, treatment information (date of treatment, treated eye, etc.) will be recorded in the subject diary.

Double blind treatment period (Visit 2~Visit 6, 0~12 weeks)

1) Baseline (Visit 2, 0 week)

At the baseline visit (Visit 2), subjects will be re-assessed for the inclusion and exclusion criteria, and those who are determined eligible for study participation will be randomized in a 1:1:1 ratio to the test group 1 (SCAI-001 eye drops 0.01%) or the test group 2 (SCAI-001 eye drops 0.02%) or the comparator group (Restasis® 0.05%). Based on the baseline test results, the study eye will be selected. The study eye will be the eye with the worse corneal staining results, i.e., the higher corneal staining score. If both eyes have the same corneal staining score, the study eye will be the eye with the worse Schirmer's test results without anesthesia, i.e., the smaller tear production without anesthesia. If both eyes have the same Schirmer's test results without anesthesia, the right eye (oculus dexter [OD]) will be the study eye.
In the morning of the baseline day, IP will not be administered, and the first dose of IP will be administered in the afternoon of the baseline day. On the baseline day, rescue medication may be used after all tests are done on that day.

Subjects who are assigned to the test group 1 or the test group 2 or the comparator group by randomization will administer IP to both eyes, twice daily at an interval of 12 hours, one drop at a time. Recommended IP dosing time is 9 AM (±1 hour) and 9 PM (±1 hour). Treatment information is to be recorded in the subject diary.

While participating in the study, subjects should complete the subject diary dispensed in this study. In the subject diary, treatment history of IP and rescue medication is to be recorded. The subject diary will be dispensed to subjects at baseline (Visit 2), Week 4 (Visit 4), and Week 8 (Visit 5) and retrieved at the respective next visit (The diary dispensed at Visit 2 will be retrieved at Visit 4) to check treatment history of IP and rescue medication.

2) Visit 3 (1 week)

Following the first IP dose at baseline, subjects will visit the institution at Week 1 (Visit 3) after morning dose of IP (if necessary, rescue medication). At this visit, subjects will be assessed for concomitant medication, vital signs for ophthalmologic examination, physical examination, laboratory tests (if necessary), ocular tolerance, best corrected visual acuity, fundus examination, slit lamp microscopy, ocular hyperemia examination, and IOP and will be checked for AEs.

3) Visit 4~6 (EOT), (4~12 weeks)

For the evaluation of efficacy and safety, subjects will visit the institution after morning dose of IP (if necessary, rescue medication) at Week 4 (Visit 4), Week 8 (Visit 5), and Week 12 (Visit 6) from the start of IP treatment. Ophthalmologic examination will be performed at least 2 hours after IP dosing (and rescue medication).
During the on-site visits, the following activities will be performed as scheduled: Schirmer's test without anesthesia, corneal staining, conjunctival staining, TBUT, Standard Patient Evaluation of Eye Dryness (SPEED) questionnaire, Ocular Surface Disease Index (OSDI), and concomitant medication for efficacy evaluation; vital signs, physical examination, laboratory tests (at Visit 6 only), pregnancy test (at Visit 6 only), ocular tolerance, best corrected visual acuity, fundus examination, slit lamp microscopy, ocular hyperemia examination, IOP, and checking for AEs for safety evaluation; and retrieving unused IP and rescue medication, dispensing IP and rescue medication (at Visits 4 and 5 only), and retrieving the subject diary and dispensing a new diary (at Visits 4 and 5 only).

All subjects will be treated with IP for 12 weeks. During IP treatment, additional tests may be performed, besides the prescheduled tests, at the discretion of the investigator (e.g., unacceptable level of worsening of symptoms), and IP treatment may be discontinued at the discretion of the investigator.
EOT is defined as a visit (Visit 6) to be performed 12 weeks after baseline (Visit 2). At EOT, subjects should visit the institution after morning dose of IP (if necessary, rescue medication), and ophthalmologic examination will be performed at least 2 hours after that.

※ Rescue medication

Whenever necessary, subjects may administer rescue medication (Refresh Plus™) provided in the study. However, it is prohibited to administer rescue medication within 15 minutes before and after IP dosing. Rescue medication is to be administered 1~2 drops at a time, no more than 3 times a day, if possible. When rescue medication is used, treatment information (date of treatment, treated eye, etc.) will be recorded in the subject diary.
At baseline (Visit 2), Week 4 (Visit 4), and Week 8 (Visit 5), a sufficient amount of rescue medication that can be used until the next visit (For Visit 2, the amount that can be used until Visit 4) will be provided for subjects, and any unused drugs will be retrieved at the subsequent visits (Visits 4, 5, and 6).

Safety follow-up period (Visit 7, 14 weeks)

Subjects will visit the institution 2 weeks after EOT for safety follow-ups and undergo safety evaluation including ophthalmologic examination and laboratory tests (if necessary).

6.2. Duration of Study

Approximately 17 months from the date of approval by the Ministry of Food and Drug Safety (MFDS) and/or Institutional Review Board (IRB)

- Subject enrollment: Approximately 6 months
- Duration of study participation for individual subjects: Approximately 5 months (1 week of screening + 2 weeks of wash-out period + 12 weeks of treatment period + 2 weeks of safety follow-up period)
- End of last subject's follow-up ~ preparation of the clinical study report (CSR): Approximately 6 months

The planned duration of study may be extended or shortened depending on subject enrollment rate, change of procedures after the end of study (EOS), etc.

6.3. Randomization

An independent statistician who is not directly involved in the study will generate a randomization table by block randomization method using a statistics software (SAS®, Version 9.4 or above, SAS Institute, Cary, NC, USA).
Centralized randomization will be performed via interactive web response system (IWRS). The investigator will assign screening numbers in the order of written informed consent and randomization numbers in the order of the determination of enrollment for finally eligible subjects based on the inclusion and exclusion criteria. Subjects will be randomized in a 1:1:1 ratio to the test group 1 (SCAI-001 eye drops 0.01%) or the test group 2 (SCAI-001 eye drops 0.02%) or the comparator group (Restasis® 0.05%). Screening and randomization numbers will be assigned according to the following rules.

For screening, a screening number will be assigned in the format of **SXXNNN**.

Character	Description
S	A letter representing screening
XX<br>NNN	Institution number<br>Serial number<br>(e.g., 001, 002, 003…)

When a subject who is eligible based on the inclusion and exclusion criteria is enrolled in the study, a randomization number in the format of **RNNN** will be assigned in a sequential manner.

Character	Description
R	A letter representing randomization
NNN	Serial number<br>(e.g., 001, 002, 003…)

6.4. Double Blinding and Unblinding

This is a double-blind study. To maintain double blind during the study, a bundle of 10 IPs will be packaged in an aluminum pouch, and then 7 aluminum pouches will be packaged in a box for shipping to the institution and supplying to subjects to ensure that the test drugs (SCAI-001 eye drops 0.01%, SCAI-001 eye drops 0.02%) and the comparator (Restasis® 0.05%) cannot be identified by appearance at the institution. For details, see 8.2 in this protocol.
If an emergency situation occurs to a subject, such as SAEs, the investigator and the sponsor should break the blind only when that is imperative for the right and safety of the subject. If unblinding is necessary, the investigator must inform the IRB and the sponsor of details of justification for unblinding.
If blind is not maintained for the investigator, Contract Research Organization (CRO) personnel, or a subject, the investigator will withdraw the subject. However, if unblinding is unlikely to affect the safety of the subject, or if there is an ethical reason, the subject may continue participating in the study at the discretion of the investigator in consultation with the sponsor.
Upon EOS and data locking, analysis sets will be finalized at the blind meeting, and afterwards, randomization codes will be opened for statistical analysis.

7. Criteria for End of Study and Premature Withdrawal

7.1. Criteria for End of Study

A subject is considered to have completed the study when he or she has completed the EOT visit (Visit 6) procedures at Week 12 from the start of IP treatment.

The safety follow-up visit (Visit 7, Week 14) is defined as the EOS for that subject. If a subject has unresolved AEs at Visit 7 and is to be followed up for AEs after Visit 7, that subsequent visit will be the EOS.

The EOS of the last enrolled subject will be defined as the EOS for the entire study.

7.2. Study Discontinuation or Premature Termination

The sponsor may prematurely discontinue the entire study or the study at a specific institution. Reasons for study discontinuation by the sponsor are as follows:

1) Failed enrollment of the planned number of subjects for the entire study or at a specific institution despite the passage of a reasonable period of time mutually agreed upon between the investigator and the sponsor.
2) New risk-benefit information that may significantly affect the continued study conduct (a specific situation occurs as a result of safety data review, etc.)
3) Violation of Good Clinical Practice (GCP), the protocol, or the contract by the institution or the investigator, the extent of which causes problems in the continued study conduct
4) Other administrative reasons that may significantly affect the continued study conduct

If the sponsor decides to discontinue or terminate the study, the sponsor will promptly notify in writing the purpose and the reasons to the IRB and the PI. If there is a site-specific regulation for notification of study discontinuation or termination, the notification will be provided accordingly.

When the PI receives a notification from the sponsor regarding study discontinuation or termination, the PI will promptly notify the purpose to the subject and ensure appropriate data and post-study processes.

If the study is discontinued, the PI is to transfer to the sponsor all data collected up to the point of discontinuation and notify in writing to the IRB.

8. Investigational Product Information and Management

8.1. Active Ingredient, Strength, and Formulation of Investigational Product

8.1.1. Test Drug

Table 1. Test drug

**Product name (code name)**	Test drug 1: SCAI-001 eye drops 0.01%<br>Test drug 2: SCAI-001 eye drops 0.02%
**Manufacturer**	DHP Korea Co., Ltd.
**Active ingredient and strength**	Cyclosporine 0.1 mg/mL or 0.2 mg/mL
**Appearance and formulation**	Clear, colorless to slightly white liquid for eye drops in a clear, colorless plastic container
**Shelf-life**	24 months from the date of manufacture
**Storage conditions**	Store in an air tight container at ambient temperature (1~30 ℃).

8.1.2. Comparator

Table 2. Comparator

**Product name (code name)**	Restasis® 0.05%
**Manufacturer**	Allergan Korea Ltd.
**Active ingredient and strength**	Cyclosporine 0.5 mg/mL
**Appearance and formulation**	White, opaque to slightly translucent, homogeneous liquid in a white, translucent container
**Shelf-life**	24 months from the date of manufacture
**Storage conditions**	Store in an air tight container at room temperature (15~25 ℃).

8.1.3. Rescue Medication

Table 3. Rescue medication

**Product name (code name)**	Refresh Plus™
**Manufacturer**	Allergan Korea Ltd.
**Active ingredient and strength**	Carboxymethylcellulose sodium 2 mg/0.4 mL
**Appearance and formulation**	Clear, colorless to pale yellow eye drops in a clear, single-use container
**Shelf-life**	24 months from the date of manufacture
**Storage conditions**	Store in an air tight container at room temperature (15~25 ℃).

8.2. Investigational Product Packaging and Labeling

In this study, IP (test drugs and comparator) will be labeled as follows, according to Regulations on Manufacturing and Quality Control of Medicinal Products (MFDS Notification) [Attachment 11] Investigational Product Manufacturing, 7.7 Labeling. IP will be then packaged and supplied to the pharmacist at the institution.

1) Statement that IP may be used only for the purpose of clinical trials (e.g., "for clinical trials")
2) IP name or identifier
3) Lot No. or Code No. to identify content and package
4) Name, address, and telephone number of the sponsor (Investigational New Drug holder)
5) Expiry date (shelf-life)
6) Storage conditions
7) Statement of "keep out of reach of children."
8) Reference code (Protocol No.) to identify the study
9) IP No.
10) Administration method

If the primary packaging container is too small to contain all of the above items in the label, only the minimum information (IP/study drug name or identifier/Lot No. or Code No./sponsor's name/reference code) will be included in the primary packaging label, according to the same regulation.

8.3. Route and Method of Administration

Once subjects who meet the inclusion and exclusion criteria are randomized in a 1:1:1 ratio to the test groups (test group 1 or test group 2) or the comparator group, they will administer IP according to their assigned group for 12 consecutive weeks, twice a day, as follows:

- Test group 1: Test drug 1 (SCAI-001 eye drops 0.01%)
- Test group 2: Test drug 2 (SCAI-001 eye drops 0.02%)
- Comparator group: Comparator (Restasis® 0.05%)

Table 4. IP administration method and duration of administration

Classification	Test group 1	Test group 2	Comparator group
Morning	○	◎	△
Afternoon	○	◎	△

○: Test drug 1(SCAI-001 eye drops 0.01%)

◎: Test drug 2(SCAI-001 eye drops 0.02%)

△: Comparator (Restasis® 0.05%)

Randomized subjects will administer IP of the respective group to both eyes, twice daily, one drop at a time. Before use, IP is to be mixed by inverting its container. The first IP dose will be administered in the afternoon of the baseline day. Recommended IP dosing time is 9 AM (±1 hour) and 9 PM (±1 hour), twice daily at an interval of 12 hours. IP will be administered to the conjunctival sac by pulling down the lower eye lid with a finger, with eyes facing upwards by tilting head back. To ensure that the administered drug does not run down, subjects will maintain the position for 30 seconds with eyes closed, followed by blinking twice or three times so that the drug can spread evenly.
When IP and rescue medication are to be used simultaneously, they should be administered with 15 minutes apart.
Treatment history of IP and rescue medication will be recorded in the subject diary.

8.4. Investigational Product Accountability, Retrieval, and Disposal

The IP provider (hereinafter the sponsor) will directly supply IP, etc. to the pharmacist in consultation with the PI. A sufficient amount of IP should be provided with the time window of the scheduled visit taken into consideration. The sponsor will include in the IP supply a receipt in which the quantity and lot number of IP, etc. are specified, and the pharmacist will receive and retain the receipt. IP should be marked with "for clinical trials".

The pharmacist will confirm by signing in writing the receipt of IP from the IP provider and the quantity of IP received, and will store and manage IP, etc. to ensure that it is not used for any purpose other than the clinical trial.
Upon the receipt of IP, the pharmacist will confirm that blind can be maintained by the appearance of IP. To maintain double blind, a bundle of 10 IPs will be packaged in an aluminum pouch, and then 7 aluminum pouches will be packaged in a box for shipping to the institution to ensure that the test drugs and the comparator cannot be identified by appearance. IP will be dispensed to subjects as packaged in a box.
IP should be dispensed via IWRS according to prescriptions signed by the physician who is participating in the study. The sub-investigator or the pharmacist should explain to subjects how and when to administer IP.
The pharmacist should record patient assignment number (or code number), date of prescription, and quantity dispensed, etc. in the drug accountability log, keep track of IP use, and retain the lists of IP receipt, dispensing, and retrieving records. To verify all IP use, the clinical research associate (CRA) will check on a regular basis the quantity of IP managed by the pharmacist.
The sub-investigator or the pharmacist will instruct subjects to return any unused IP to the institution at the next visit; Subjects are to return unused IP to an unblinded sub-investigator or pharmacist. In each institution, a person responsible for retrieving unused IP will be designated in advance, and IP will be retrieved only by this person so that all others involved remain blinded. Any loss of IP at the institution should be documented by the sub-investigator or the pharmacist. Upon study discontinuation or EOS, the sponsor will retrieve and dispose of any unused IP. In consultation with the PI, the unblinded sub-investigator or pharmacist will return unused IP to the sponsor and retain the receipt.
In any case, the investigator will ensure that IP and related materials are not provided to other investigators and are not used for any purpose other than those specified in the protocol.

9. Administration Plan

9.1. Administration and Treatment Schedules

According to randomization, SCAI-001 eye drops 0.01%, SCAI-001 eye drops 0.02%, or Restasis® 0.05% will be administered for 12 weeks in the test group 1, the test group 2, and the comparator group, respectively. IP will be administered to the eyes every day in the morning and in the evening, 12 hours apart.
For details, see 8.3 Route and Method of Administration.

9.2. Concomitant Medication

During the study, concomitant medication that is considered to have no impact on efficacy and safety evaluations and the interpretation of results and any drugs and therapies not listed in 9.3 Prohibited Drugs and Therapies will be permitted for use at the discretion of the investigator. For concomitant medication used during the study, details of the drug, i.e., brand name, administered dose, duration of administration, reason for administration, route of administration, unit of dose, etc. will be recorded in electronic CRF (eCRF).

9.3. Prohibited Drugs and Therapies

During the study, the following drugs and therapies that are considered to affect efficacy and safety evaluations and the interpretation of results will be prohibited for use.

9.3.1. Prohibited Drugs

1. Any ophthalmic drugs that may affect the results of the study (e.g., steroidal/non-steroidal anti-inflammatory eye drops, etc.)
2. Cyclosporine eye drops
3. Systemic steroids; and local steroids such as ophthalmic, inhaled, or subcutaneously injected steroids (Use of topical steroid ointments for any purpose other than for eyes will be permitted.)
4. Immunosuppressants (azathioprine, tacrolimus, cyclosporine, mycophenolate mofetil, etc.)
5. Drugs that may affect DED (oral contraceptives, anticholinergics, tricyclic antidepressants, antihistamines, hypnotics, diuretics, antimuscarinics, beta blockers, retinoids, aspirin, NSAIDs, etc.). However, drugs that have been administered for ≥ 30 days prior to screening without change of dosage and administration will be permitted, provided that they will be administered throughout the study with the same dosage and administration.
6. Health supplements (vitamin A, lutein, omega-3, snake oil, etc.) that may affect DED

9.3.2. Prohibited Therapies

1. Intraocular procedure and surgery during the study
2. Use of contact lenses during the study
3. Use of punctual plugs
4. Use of other IP or investigational device

9.4. Rescue Medication

Rescue medication will be used during the washout period when DED treatment that has been used before the start of IP treatment will be stopped. After IP dosing, rescue medication may be used as necessary to alleviate the symptoms of DED. For supportive therapy, this study will provide Refresh Plus™ as rescue medication (rescue medication).
For rescue medication to be used in this study, see 8.1.3 Rescue Medication.

9.5. Treatment Compliance

When IP is prescribed, a sufficient amount of IP will be provided for administration until before the next visit, and subjects will be instructed to return any unused drugs at the next visit. During the study, IP treatment in each eye will be recorded in the subject diary, and at each visit, actually administered IP doses will be counted using the subject diary dispensed at the previous visit. Treatment compliance will be calculated based on the number of actually administered IP doses. Treatment compliance during the treatment period will be calculated as follows:

$$\text{Compliance (\%)} = \frac{\text{Number of IP doses actually administered during the period}}{\text{Number of IP doses planned to be administered}} \times 100$$

10. Study Procedures and Assessments

10.1. Assessment Items by Visit

10.1.1. Visit 1 (Screening/wash-out period, ~-3 weeks)

Patients who are potentially eligible based on the judgment of the investigator will be asked to participate in the study. The investigator or a designated sub-investigator will sufficiently explain to subjects or their guardians (legally acceptable representatives) details of the study procedures based on the subject information sheet and provide them the subject information sheet in order to allow all subjects and their guardians to have sufficient time to read and discuss the subject information sheet and make a decision. For patients who consent to study participation, the subjects or their legally acceptable representatives will voluntarily sign the written ICF prior to any of the study related procedures and will be provided a copy of the signed ICF.

Among those who consent to study participation and are eligible based on the inclusion and exclusion criteria, subjects who have ongoing DED treatment will undergo a wash-out period during which the DED treatment will be discontinued. If there is no ongoing DED treatment at the start of study participation, the wash-out period may be skipped. Therefore, this period of approximately 3 weeks will consist of approximately 1 week of the screening period and at least 2 weeks of the wash-out period.

[Observations and clinical test items]

1) Written informed consent	
2) Screening number assigning	
3) Demographics	
4) Prior medication and treatment	
5) Medical/surgical history	
6) Basic disease information	
7) Body measurement	
8) Vital signs/physical examination	
9) Laboratory tests	Results obtained within 4 weeks prior to screening may be used, if available.
10) Pregnancy test	
11) ECG	Results obtained within 4 weeks prior to screening may be used, if available..
12) Best corrected visual acuity	
13) Fundus examination and slit lamp microscopy	
14) Ocular hyperemia	
15) TBUT	
16) Corneal staining	
17) Schirmer's test without anesthesia	
18) IOP	
19) Inclusion and exclusion criteria checking	
20) Rescue medication dispensing	Rescue medication will be dispensed for use during the wash-out period.
21) Subject diary dispensing	Treatment information for rescue medication during the wash-out period will be recorded.
22) Appointing the date of next visit	

10.1.2. Visit 2 (Baseline [Randomization], Day 0)

The subject's eligibility for study participation will be finally determined, and IP will be prescribed according to randomization. Visit 2(Baseline) should be performed within 3 weeks after Visit 1(Screening). For those who do not need a wash-out period at the screening visit, this visit may be performed simultaneously with the screening visit.

Rescue medication that has been used during the wash-out period is not to be administered from the day before the baseline visit, and IP will be used starting from the afternoon of the baseline day.

[Observations and clinical test items]

1) Prior/concomitant medication and treatment history	
2) Vital signs/physical examination	
3) Pregnancy test	
4) SPEED questionnaire	
5) OSDI	
6) Best corrected visual acuity	
7) Fundus examination and slit lamp microscopy	
8) Ocular hyperemia	
9) TBUT	
10) Corneal staining	
11) Conjunctival staining	
12) Schirmer's test without anesthesia	
13) IOP	
14) Inclusion and exclusion criteria checking	
15) Study eye selection	
16) Randomization number assigning	
17) Unused rescue medication retrieving	Any unused rescue medication left over after the washout period will be retrieved.
18) Rescue medication dispensing	Rescue medication will be dispensed for use until the next visit (Visit 3).
19) IP dispensing	
20) Subject diary retrieving	The subject diary with recordings during the wash-out period will be retrieved.
21) Subject diary dispensing	The subject diary will be dispensed for recording until the next visit (Visit 3).
22) AEs checking	AEs collected at baseline are defined as non-treatment emergent AEs (non-TEAEs).
23) Appointing the date of next visit	

10.1.3. Visit 3 (1 week [±3 days])

This visit will be performed 1 week after the start of IP treatment for ophthalmologic examination and safety follow-ups. On the visit day, subjects will visit the institution after morning dose of IP (if necessary, rescue medication).

[Observations and clinical test items]

1) Concomitant medication and treatment	
2) Vital signs/physical examination	
3) Laboratory tests	To be performed as necessary.
4) Ocular tolerance	
5) Best corrected visual acuity	
6) Fundus examination and slit lamp microscopy	
7) Ocular hyperemia	
8) IOP	
9) AEs checking	
10) Appointing the date of next visit	

10.1.4. Visit 4, Visit 5 (Double-blind treatment period, 4 weeks [±4 days], 8 weeks [±4 days])

This visit is for efficacy and safety evaluations after the start of IP treatment. On the visit day, subjects should visit the institution after morning dose of IP (if necessary, rescue medication), and ophthalmologic examination will be performed at least 2 hours after IP dosing (and rescue medication).

[Observations and clinical test items]

1) Concomitant medication and treatment	
2) Vital signs/physical examination	
3) SPEED questionnaire	
4) OSDI	
5) Ocular tolerance	
6) Best corrected visual acuity	
7) Fundus examination and slit lamp microscopy	
8) Ocular hyperemia	
9) TBUT	
10) Corneal staining	
11) Conjunctival staining	
12) Schirmer's test without anesthesia	
13) IOP	
14) Unused rescue medication retrieving	Any unused rescue medication after dispensing at Visit 2 and Visit 4 will be retrieved at Visit 4 and Visit 5, respectively.
15) Rescue medication dispensing	
16) Unused IP retrieving	Any unused IP after dispensing at Visit 2 and Visit 4 will be retrieved at Visit 4 and Visit 5, respectively.
17) IP dispensing	
18) Subject diary retrieving	The subject diary dispensed at Visit 2 and Visit 4 will be retrieved at Visit 4 and Visit 5, respectively.
19) Subject diary dispensing	
20) AEs checking	
21) Appointing the date of next visit	

10.1.5. Visit 6 (EOT, 12 weeks [±4 days])

This is the EOT visit for efficacy and safety evaluations.
At EOT, subjects should visit the institution after morning dose of IP (if necessary, rescue medication), and ophthalmologic examination will be performed at least 2 hours after IP dosing (and rescue medication).

[Observations and clinical test items]

1) Concomitant medication and treatment	
2) Vital signs/physical examination	
3) Laboratory tests	
4) Pregnancy test	
5) SPEED questionnaire	
6) OSDI	
7) Ocular tolerance	
8) Best corrected visual acuity	
9) Fundus examination and slit lamp microscopy	
10) Ocular hyperemia	
11) TBUT	
12) Corneal staining	
13) Conjunctival staining	
14) Schirmer's test without anesthesia	
15) IOP	
16) Unused rescue medication	Any unused rescue medication after dispensing at Visit 5 will be retrieved at

retrieving	Visit 6.
17) Unused IP retrieving	Any unused IP after dispensing at Visit 5 will be retrieved at Visit 6.
18) Subject diary retrieving	The subject diary dispensed at Visit 5 will be retrieved at Visit 6.
19) AEs	
20) Appointing the date of next visit	

10.1.6. Visit 7 (14 weeks [±4 days])

This visit will be performed 2 weeks after EOT for safety follow-up. During this on-site visit, subjects will be evaluated for safety by the investigator or his or her designee who will perform laboratory tests and collect applicable information including status of AEs and date of resolution, etc.

[Observations and clinical test items]

1) Concomitant medication and treatment	
2) Vital signs/physical examination	
3) Laboratory tests	To be performed as necessary.
4) Ocular tolerance	
5) Best corrected visual acuity	
6) Fundus examination and slit lamp microscopy	
7) Ocular hyperemia	
8) IOP	
9) AEs	

10.1.7. Unscheduled Visits

Unscheduled visits may be performed at any time upon the subject's request or whenever necessary at the discretion of the investigator. If a subject had a visit on an unscheduled day, vital signs, physical examination, laboratory tests, pregnancy test, ophthalmologic examination, AEs, and concomitant medication and treatment checking may be performed as necessary.

10.2. Assessment Methods

10.2.1. Written Informed Consent and Screening Number Assigning

During a subject's first visit to the institution for study participation, the investigator will explain the study to the subject, answer his or her questions, and ask him or her to participate in the study. If the subject agrees to participate in the study, he or she will be assigned a screening number according to '6.3 Randomization'.

10.2.2. Demographics

Demographic information including initials, age, month and year of birth, and childbearing potential will be collected.

10.2.3. Prior/Concomitant Medication and Treatment History

1. Prior medication and treatment history: Information within 30 days prior to the screening visit or the baseline visit will be collected. History of medication and treatment to treat DED or to alleviate its symptoms, including artificial eye drops, will be collected without a time limit.
2. Concomitant medication and treatment history: At each visit after IP dosing during Visit 2, the investigator will check for concomitant medication. If a subject had a concomitant medication (including treatment for other diseases or AEs), detailed information on that drug (brand name [name of active ingredient], purpose of administration, administered

dose, start date, end date, etc.) will be recorded in the CRF.

10.2.4. Medical and Surgical History

Information on past medical and surgical history and concurrent diseases within 30 days prior to the screening visit will be collected. However, as for malignant tumors, information within 5 years prior to the screening visit will be collected. The target disease (DED) of the study will not be collected as a concurrent disease.
For each medical history (past medical history, surgical history, and concomitant disease), information on progress at the time of screening will be collected. Ophthalmologic medical and surgical history will be collected without a time limit.

10.2.5. Basic Disease Information

Date of diagnosis of DED (duration of disease), status and duration of contact lenses use, and DED related symptoms (sandiness/grittiness, dryness, light sensitivity, pain or soreness, blurred vision, etc.), etc. will be collected.

10.2.6. Body Measurement

Height and weight will be measured at Visit 1.

10.2.7. Vital Signs/Physical Examination

1. Vital signs: At each visit, body temperature, blood pressure (diastolic/systolic), and pulse rate will be measured for vital signs. Subjects are to be tested after at least 5 minutes of rest.
2. Physical examination: At each visit, ‘appearance’, ‘skin’, ‘head/neck’, ‘heart, ‘abdomen’, ‘urogenital system’, ‘limbs’, ‘musculoskeletal system’, ‘nervous system’, ‘lymph nodes’, and ‘others’ will be assessed by visual inspection, palpation, percussion, and interview.

Clinically significant abnormalities identified at Visit 1 will be collected as medical history. After Visit 1, clinically significant abnormalities compared with the last visit will be collected as AEs.

10.2.8. Laboratory Tests

At Visit 1, Visit 3 (if necessary), Visit 6, and Visit 7 (if necessary), the investigator will perform laboratory tests for the following parameters. For Visit 1, results obtained within 4 weeks prior to screening may be used, if available.

- Hematology: WBC, RBC, Hemoglobin, Hematocrit, Platelet count, WBC diff count.
- Blood biochemistry: Na, K, Ca, Cl, CPK, BUN, Creatinine, Uric acid, Total bilirubin, Albumin, Total protein, ALT, AST, γ-GTP, Alkaline phosphatase, Glucose, HbA1c (HbA1c will be tested at screening only.)
- Serum hepatitis B and C: hepatitis B virus antigen (HBsAg), hepatitis C virus antibody (HCV Ab) (to be tested at screening only.)
- Urinalysis: Protein, Glucose, Blood, Ketones

Clinically significant abnormalities identified at Visit 1 will be collected as medical history. After Visit 1, clinically significant abnormalities compared with the last visit will be collected as AEs.

10.2.9. Pregnancy Test

To be performed at Visit 1, Visit 2, and Visit 6. Pregnancy will be tested by the investigator using urine hCG pregnancy test only in women of childbearing potential prior to menopause. Menopause is defined as no menstruation for $\geq$ 1 year.

10.2.10. Electrocardiography

At Visit 1, 12-lead electrocardiography (ECG) will be performed. For Visit 1, results obtained within 4 weeks prior to screening may be used, if available.

10.2.11. Assessment of Inclusion and Exclusion Criteria and Randomization

Based on observations and tests done at Visit 1 and Visit 2, the subject's eligibility for study participation will be assessed according to the inclusion and exclusion criteria. Only those who are finally confirmed to be eligible at Visit 2 will be randomized according to '6.3 Randomization'.

10.2.12. Efficacy Evaluation Methods

10.2.12.1. SPEED Questionnaire Assessment

SPEED is a questionnaire developed to assess the frequency and severity of dry eye symptoms felt by patients.
It consists of 3 items: time of symptoms (at this time, within past 72 hours, within past 3 months), frequency, and severity. For each of the frequency and severity items, the same 4 symptoms (dryness, grittiness or scratchiness; soreness or irritation; burning or watering; eye fatigue) will be provided.
Frequency will be assessed for each symptom as 'never (0)' 'sometimes (1)', 'often (2)', and 'constant (3)'. Severity will be assessed for each symptom as 'no problems (0)' 'tolerable - not perfect, but not uncomfortable (1)', 'uncomfortable - irritating, but does not interfere with my day (2)', 'bothersome - irritating and interferes with my day (3)', and 'intolerable - unable to perform my daily tasks (4)'. Total score will range between 0 and 28. The time of symptoms item will not be included in the score.[5]

10.2.12.2. Ocular Surface Disease Index

OSDI, a tool to assess ocular surface disease, consists of ocular symptoms (5 questions), vision related function (4 questions), and environmental factors that may cause the symptoms (3 questions). Each of these 12 questions will be assessed on a 5-point scale: 0 if the patient has the symptom none of the time, 1 for some of the time, 2 for moderate symptoms (half of the time), 3 for most of the time, and 4 for all of the time.
The OSDI score will be calculated on a scale of 0 to 100 as follows, with higher scores representing severer symptoms.

$$\text{OSDI score} = \frac{\text{Sum of the scores of all items answered} \times 100}{\text{Total number of questions answered} \times 4}$$

The OSDI score will be graded into normal (0~12), mild (13~22), moderate (23~32), and severe (33) and assessed as change in grade and score.

10.2.12.3. Tear Film Break-up Time

After fluorescein staining under a slit lamp with cobalt blue illumination, the patient will be asked to blink eyes. TBUT will be measured as time in seconds from the last blink to the appearance of a black spot or streak or fluorescein defect in the stained tear film. Measurements will be taken 3 consecutive times on the same eye, and results will be recorded in the unit of 1/100 seconds.

- **<u>Fluorescein staining</u>**
  Fluorescein staining will be performed for TBUT and corneal staining. Fluorescein staining for TBUT and corneal staining will not be performed on both eyes simultaneously; The right eye will be stained and tested, and subsequently, the left eye will be stained and tested.
  One drop of preservative-free saline will be placed on the fluorescein staining reagent strip with dye. Then, the strip will be touched on the lower conjunctival sac. The patient will be asked to blink 3-4 times to distribute the

dye in the tear film.

10.2.12.4. Corneal Staining

After fluorescein staining under a slit lamp with cobalt blue illumination using a yellow filter, the extent of corneal staining will be assessed on a 6-point scale according to Oxford grading system[22]. For assessment criteria, see Table 5. Oxford grading system.

Table 5. Oxford grading system

Panel	Staining pattern	Grade	Criteria
A	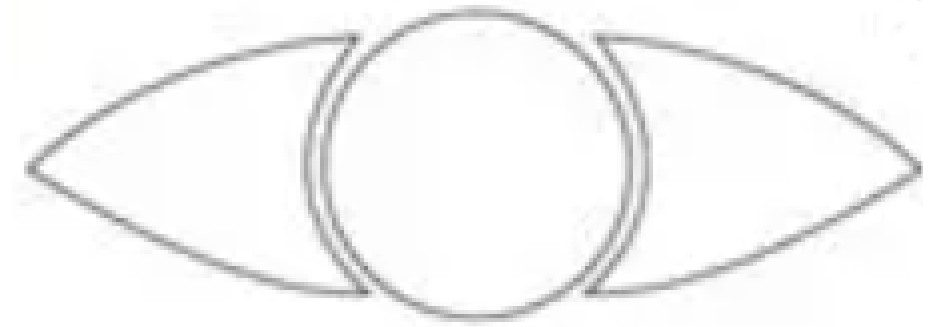	0	Equal to or less than panel A
B	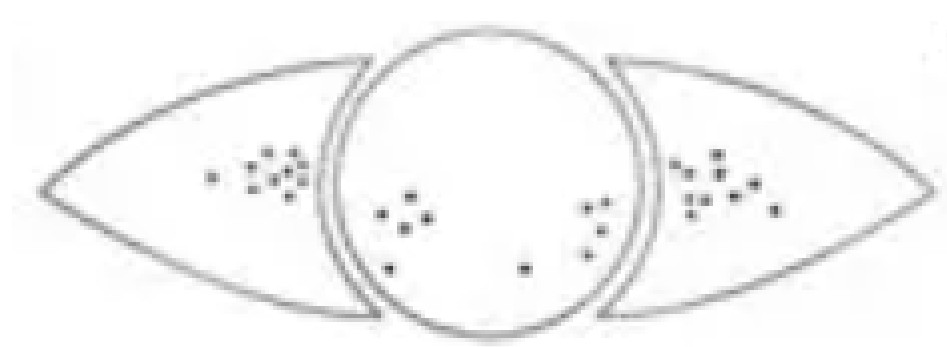	I	Equal to or less than panel B, greater than A
C	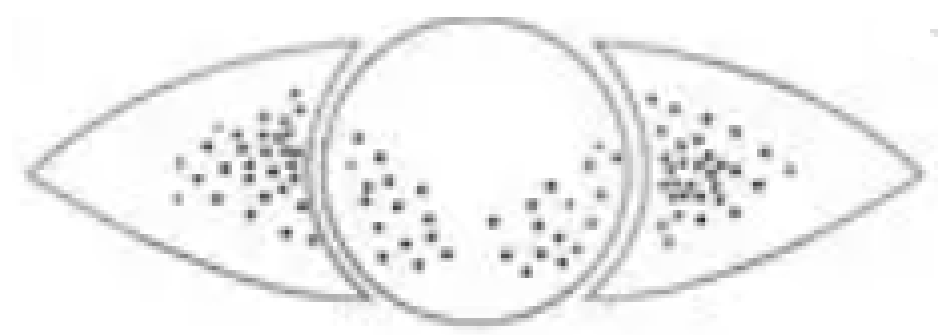	II	Equal to or less than panel C, greater than B
D	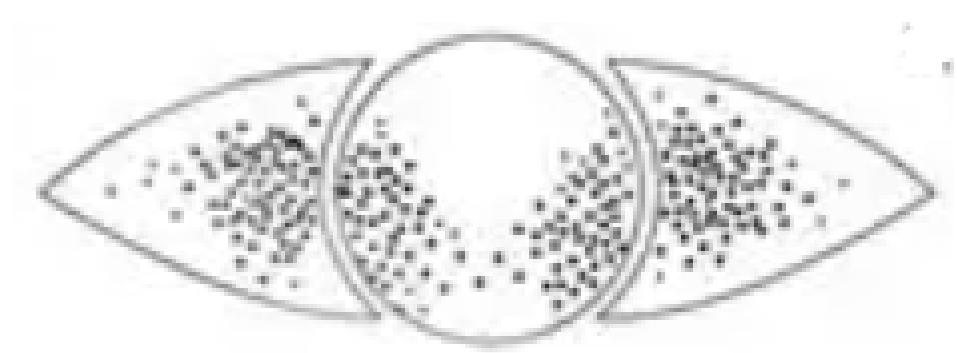	III	Equal to or less than panel D, greater than C
E	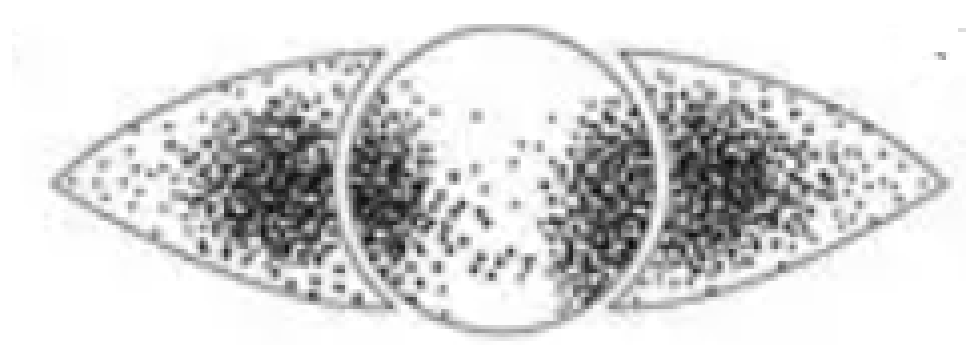	IV	Equal to or less than panel E, greater than D
>E		V	Greater than panel E

10.2.12.5. Conjunctival Staining

After lissamine green staining, the extent of conjunctival staining will be assessed by slit lamp microscopy on a 6-point scale according to Oxford grading system. Conjunctiva will be divided into 2 regions (nasal, temporal conjunctiva) for assessment. For assessment criteria, see Table 5. Oxford grading system.

- **<u>Lissamine green staining</u>**
  One drop of preservative-free saline will be placed on the lissamine green staining reagent strip with dye. Then, the strip will be touched on the lower conjunctival sac. The patient will be asked to blink 3-4 times to distribute the dye in the tear film.

10.2.12.6. Schirmer's Test without Anesthesia

Schirmer's test without anesthesia is a test of tear secretion function and assesses tear secretion function as a sum of basal tear secretion and reflex tear secretion. Without topical ocular anesthesia, a Schirmer strip will be touched for 5 minutes

on the bulbar conjunctiva at the point of 1/3 of the outer side of the lower eyelid, and the area wet by tear will be measured in millimeters.
The strip will be inserted into the right eye, and then into the left eye. After 5 minutes with the eyes closed, the strip will be removed in the order of insertion, first from the right eye, and then from the left eye. If the wet margin is diagonal, the length to the middle point will be measured.

10.2.13. Ocular Safety Evaluation Methods

10.2.13.1. Ocular Tolerance Assessment

Subjects will be assessed for ocular AEs after treatment with the test drug or the comparator. Subjects will be assessed for 7 symptoms: stinging/burning, itching, blurred vision, sandiness/grittiness, dryness, light sensitivity, and pain or soreness. Severity will be assessed as 4 grades, and duration of symptoms will be assessed as 2 stages.
At Visit 7, only the severity of the 7 tolerance symptoms will be assessed, and the duration of symptoms will not be assessed.

Table 6. Severity and duration of tolerance symptoms

Grade	Score	Severity
None	0	No symptoms.
Mild	1	Symptoms do not interfere with daily activities.
Moderate	2	Symptoms somewhat interfere with daily activities.
Severe	3	Symptoms severely interfere with daily activities.

Stage	Duration
Immediate	Disappears within 5 minutes after treatment
Persistent	Persistent for ≥ 5 minutes after treatment

10.2.13.2. Best Corrected Visual Acuity

Best corrected visual acuity should be tested using Snellen acuity charts. If Snellen acuity charts are not used, the measurement can be used by converting to the value of Snellen acuity charts.

10.2.13.3. Fundus Examination

Retina, optic disk, macula, and choroid are located in the fundus, and this area will be observed by camera. Abnormal regions inside the eye can be examined via the pupil.
If the pupils are small, dilating drops may be administered to the eyes for testing.
Clinically significant abnormalities identified at Visit 1 will be collected as medical history. After Visit 1, clinically significant abnormalities compared with the last visit will be collected as AEs.

10.2.13.4. Slit Lamp Microscopy

Slit lamp microscopy is a test to examine disease inside the eyes without ocular staining. It is performed by shining a thin, long, and bright light into the eyes.
Clinically significant abnormalities identified at Visit 1 will be collected as medical history. After Visit 1, clinically significant abnormalities compared with the last visit will be collected as AEs.

10.2.13.5. Ocular Hyperemia Examination

The extent of ocular hyperemia will be assessed as 0 (normal) to 4 (severe) according to Table 7. Criteria for the assessment of ocular hyperemia.

Table 7. Criteria for the assessment of ocular hyperemia

Grade	Extent of ocular hyperemia	Description
0(normal)	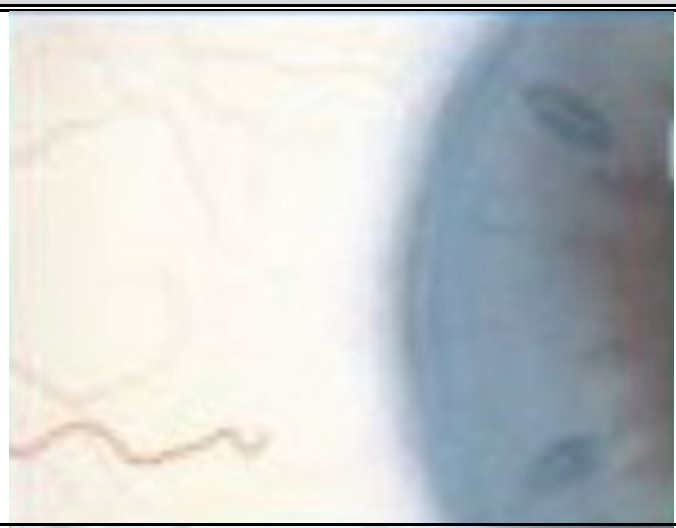	White ocular conjunctiva<br>Involvement of a major conjunctival blood vessel<br>No corneal involvement
1(trace)	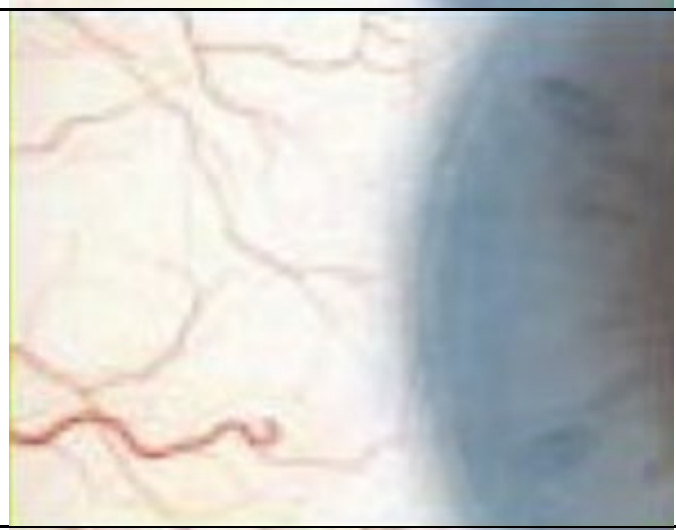	Minor change in conjunctival hyperemia (redness)<br>Major conjunctival vasodilation and hyperemia
2(mild)	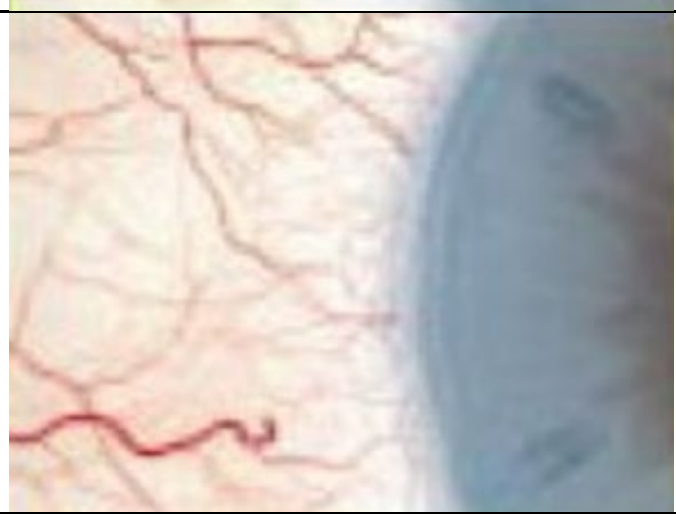	Mild change in conjunctival hyperemia (redness) and corneal limbus hyperemia (redness)
3(moderate)	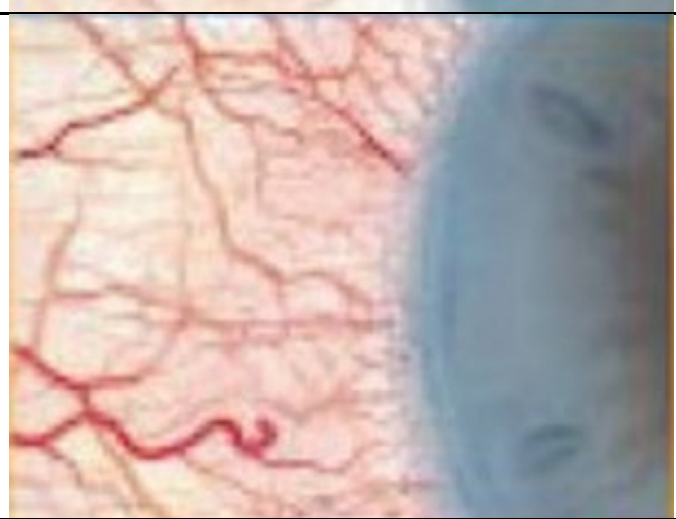	Moderate change in conjunctival hyperemia (redness) and corneal limbus hyperemia (redness)
4(severe)	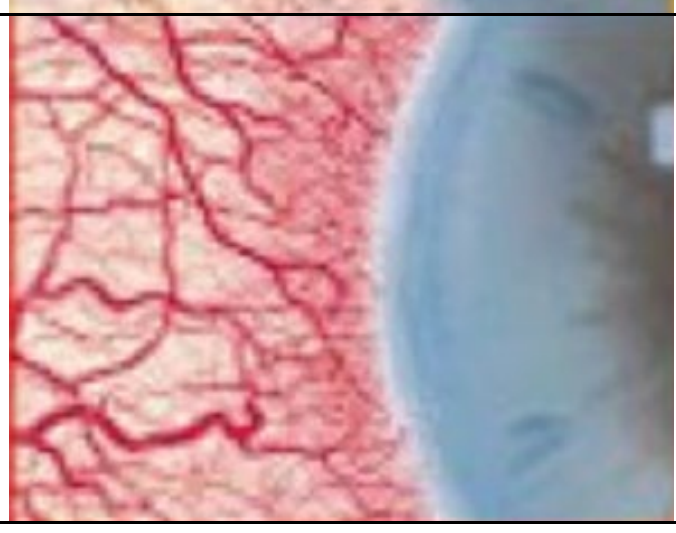	Severe change in conjunctival hyperemia (redness) and corneal limbus hyperemia (redness)

Reference: Nathan Efron, BScOptom, et al. "Contact Lens Complications, 3rd Edition" (2012).

10.2.13.6. Intraocular Pressure

The pressure inside the eyes will be measured using a noncontact tonometer. In both eyes, IOP will consecutively be measured twice per eye, and results will be recorded. If re-measuring is necessary based on the judgment of the investigator due to difference in IOP between the two eyes, IOP will additionally be measured twice, and the mean of the results will be collected as IOP.

10.2.14. Rescue Medication Dispensing and Retrieving

At Visits 1, 2, 4, and 5, a sufficient amount of rescue medication that can be used until the next visit (For Visit 2, the amount that can be used until Visit 4) will be dispensed, and any unused rescue medication after treatment will be retrieved at the subsequent visits (Visits 4, 5, and 6).

10.2.15. Investigational Product Dispensing and Retrieving

At Visits 2, 4, and 5, a sufficient amount of IP that can be used until the next visit (For Visit 2, the amount that can be used until Visit 4) will be dispensed, and any unused IP after treatment will be retrieved at the subsequent visits (Visits 4, 5, and 6).

During the treatment period, IP will be prescribed for twice daily administration; One IP is to be used per time, one drop at a time for both eyes (2 IPs are to be used per day). At baseline, Week 4, and Week 8, IP will be prescribed for 35 days of treatment i.e., 28 days +4 days (plus 3 spare days).

10.2.16. Subject Diary Dispensing and Retrieving

To record treatment information (date of treatment, treated eye, etc.) for IP and rescue medication, all subjects should complete the subject diary during the study period including the wash-out period. At Visits 1, 2, 4, and 5, the subject diary will be dispensed for recording until the next visit (at Visit 2, for recording until Visit 4). At Visits 2, 4, 5, and 6, the subject diary dispensed at the previous visit will be retrieved.
The subject diary dispensed at Visit 1 will be used for recording treatment information (date of treatment, treated eye, etc.) for rescue medication during the wash-out period and will be retrieved at Visit 2.

10.2.17. Assessment of Adverse Events

All AEs occurring from after informed consent to the safety follow-up visit (Visit 7) will be identified and recorded. At each visit, new AEs since the last visit will be identified and recorded, and status of resolution (outcome) will be checked for AEs reported up to the previous visit. If possible, any unresolved AEs at the safety follow-up visit (Visit 7) will be followed up until resolution. To follow up AEs, the investigator or his or her designee will collect relevant information including status and date of resolution of AEs via a telephone contact (telephone visit) or an on-site visit.

10.2.18. Concomitant Medication and Treatment

At each visit after IP dosing during Visit 2, the investigator will check for concomitant medication. If a subject had a concomitant medication (including treatment for other diseases or AEs), detailed information on that drug (brand name [name of active ingredient], purpose of administration, administered dose, start date, end date, etc.) will be recorded in the CRF.

10.3. Endpoints

10.3.1. Primary Efficacy Endpoint

Change from baseline in tear production (Schirmer's test) at Week 12

10.3.2. Secondary Efficacy Endpoints

1. Change from baseline in tear production (Schirmer's test) at Week 4 and Week 8
2. Proportion of subjects with a $\geq$ 10 mm/5 min increase or above from baseline in tear production (Schirmer's test) at Week 4, Week 8, and Week 12
3. Change from baseline in corneal staining score – Oxford grading at Week 4, Week 8, and Week 12
4. Change from baseline in conjunctival staining score – Oxford grading at Week 4, Week 8, and Week 12
5. Change from baseline in TBUT at Week 4, Week 8, and Week 12
6. Change from baseline in DED questionnaire score (SPEED) at Week 4, Week 8, and Week 12
7. Change from baseline in OSDI at Week 4, Week 8, and Week 12
8. Total number of administered doses of rescue medication during the study

10.3.3. Safety Endpoints

1. Treatment emergent AEs (TEAEs): Including adverse drug reactions (ADRs), SAEs, SADRs, unexpected AEs (UAEs), and unexpected ADRs (UADRs)
2. Ocular tolerance test
3. Best corrected visual acuity
4. Ophthalmologic examination (IOP, fundus examination, slit lamp microscopy, and ocular hyperemia examination)
5. Vital signs
6. Laboratory tests

10.4. Definition and Reporting of Adverse Events

The investigator is responsible for reporting all AEs observed or reported during the study, regardless of their relationship to IP or their clinical significance. Subjects will be instructed to contact the PI or the sub-investigator at any time when they experience any symptoms. All AEs occurring during the study after informed consent will be reported in detail in the CRF. If an AE occurs, the PI or the sub-investigator will start appropriate treatment based on his or her medical judgment. If possible, any unresolved AEs at the safety follow-up visit (Visit 7) will be followed up until resolution. To follow up AEs, the investigator or his or her designee will collect relevant information including status and date of resolution of AEs via a telephone contact (telephone visit) or an on-site visit.

10.4.1. Definition of Adverse Events

1) An **AE** is any unfavorable and unintended sign (including laboratory test abnormalities, etc.), symptom, or disease occurring to a subject, which does not necessarily have to have a causal relationship to IP.

2) A **TEAE** is any new AE occurring to a subject treated with IP. This includes not only AEs occurring after IP dosing but also worsening of the existing disease in manifestations or in severity compared with baseline.

3) An **ADR** is any unfavorable and unintended response occurring at any dose of IP whose relationship to IP cannot be ruled out.

4) An **SAE** is any AE or ADR that:
(1) Results in death or is life-threatening;
(2) Requires inpatient hospitalization or prolongation of existing hospitalization;
* The followings do not constitute hospitalization:
  - Emergency room visit or hospital stay for < 24 hours
  - Hospitalization for elective or planned surgery
  - Hospitalization for the purpose of nursing and aesthetics
  - Ambulatory surgery (outpatient, same-day surgery)
  - Hospitalization that is not associated with occurrence of new AEs or worsening of the existing conditions (Hospitalization at rehabilitation, nursing home, nursing hospitals or for respite care since before IP treatment; and hospitalization for retesting for laboratory test abnormalities, etc.)

(3) Results in persistent or significant disability/incapacity;
(4) Is a congenital anomaly/birth defect;
(5) Other important medical events not listed in 1) to 4), such as development of drug dependence or abuse or blood dyscrasias; or
(6) Others specified in the protocol

5) An **UAE** is an event that differs from an ADR in profile or in the extent of harm in light of available drug information

such as Investigator's Brochure (IB) or package insert, etc.

10.4.2. Expected Adverse Events with Investigational Product and Precautions for Use

To date, the test drug, SCAI-001 eye drops, has not been studied in humans. Therefore, the safety data of the test drug confirmed in clinical trials does not exist. Nevertheless, given that SCAI-001 eye drops have the same active ingredient (cyclosporine) and formulation (eye drops) as the comparator, Restasis® 0.05%, and the strengths of the active ingredient of SCAI-001 eye drops are smaller than that of Restasis® 0.05%, expected AEs with SCAI-001 eye drops and precautions for use are anticipated to be similar to or more favorable than those of Restasis® 0.05%.
AEs with Restasis® 0.05% and precautions for use that are specified in the label of South Korea are as follows.
.

<AEs>

1) Most common AEs: Burning sensation in the eyes (17%)
2) AEs reported in 1~5% of patients: Conjunctival hyperemia, discharge, epiphora, eye pain, sandiness/grittiness, itchy sensation, stinging pain, visual disturbance (occasionally, blurred vision)
3) AEs reported in post-marketing surveillance with unknown severity and frequency include eye swelling, hypersensitivity reactions (rarely, severe angioedema, face swelling, tongue swelling, pharyngeal edema, and dyspnea), ocular surface damage (due to vial tip touching the eye), burning sensation, itching, urticaria, etc.
4) Post-marketing surveillance in South Korea

In a post-marketing surveillance study which was conducted in South Korea for 6 years in 895 patients with ocular inflammation associated with keratoconjunctivitis sicca, the incidence of AEs regardless of causal relationship was 7.87%(70/895 patients, 93 events).

- ADRs of which causal relationship to this drug cannot be ruled out: 6.48%(58/895 patients, 76 events); burning sensation at the application site, 26 events; lack of efficacy, 11 events; xerophthalmia and eye pain, each 7 events; conjunctival hyperemia, 6 events; abnormal sensation in the eyes, 5 events; eye strain and eye irritation, each 3 events; application site itching, 2 events; and corneal erosion, sticky eyes, epiphora, and eye edema, each 1 event.
- No SADRs were reported.
- UADRs: 2.68%(24/895 patients, 27 events); lack of efficacy, 11 events; xerophthalmia, 7 events; eye strain, 3 events; eye irritation, 3 events; corneal erosion, 1 event; sticky eyes, 1 event; and eye edema, 1 event.

<This drug is contraindicated for use in the following patients.>

1) Patient with active eye infection
2) Patient suspected to have hypersensitivity reactions to any of the components of this drug

<General precautions>

1) This drug is for use in the eyes only.
2) To avoid eye injuries, caution should be exercised not to have the container touch the eyes.
3) When this drug is applied to the eyes, temporary blurred vision may occur. Do not drive or operate dangerous machines until vision becomes clear.

<Precautions for application>

1) Once it is opened, use the drug immediately only once, and discard the remaining solution and the container immediately.
2) Make sure that the tip of the container is not contaminated and does not touch the eyes or the external surface.
3) This drug should not be administered to the eyes wearing contact lenses. If you are wearing contact lenses, remove the contact lenses before administering this drug, and you may wear your contact lenses back 15 minutes after administration.

< Precautions for storage>

1) Keep out of reach of children.
2) Discard this drug if it is expired.

10.4.3. Recording of Adverse Events

1) Profile of Adverse Events

It is preferred to describe a comprehensive diagnosis or syndrome rather than individual symptoms/signs. The investigator should report AEs using standard medical terms. Information in CRF should be consistent with that in source documents.

2) Onset date (start date) and resolution date

Onset date: The start date of an AE, or the date of the laboratory test in which a clinical laboratory test abnormality is reported as an AE.

Resolution date: The date an AE is resolved; If an AE involves multiple signs/symptoms, the duration of the AE will be determined by the longest lasting sign/symptom.

3) Assessment of severity of AEs

The investigator will classify the intensity (severity) of AEs based on the following criteria:

① Mild: Dose not require treatment. Dose not greatly interfere with normal activities (function).

② Moderate: Significantly interferes with normal activities (function). Treatment may be required. Recovers upon treatment.

③ Severe: Results in incapacity. Unable to conduct work or daily activities.

4) Assessment of causal relationship of AEs

The investigator will assess causal relationship between AEs and IP based on the following criteria. AEs that are assessed as unlikely can be considered to be ‘not related’ (non-ADRs); AEs that are assessed as other classifications will be considered to be related (ADRs).

① Certain
A clinical event occurring in a plausible time relationship to drug administration, and which cannot be explained by concurrent disease, or other drugs or chemicals. The response to drug withdrawal is clinically plausible. The event is definitive pharmacologically or phenomenologically upon re-challenge, if necessary.

② Probable, likely
A clinical event with a reasonable time sequence to administration of drug, unlikely to be attributed to concurrent disease, or other drugs or chemicals, and which follows a clinically reasonable response on drug withdrawal (re-challenge information is not available).

③ Possible
A clinical event with a reasonable time sequence to administration of drug, but which can also be explained by concurrent disease, other drugs or chemicals. Information on drug withdrawal may be lacking or unclear.

④ Unlikely
A clinical event with a temporal relationship to drug administration which makes a causal relationship improbable, and in which other drugs, chemicals or underlying disease provide plausible explanations.

⑤ Conditional, unclassified
A clinical event about which more data is essential for a proper assessment or the additional data are under examination.

⑥ Unassessable, unclassifiable
The event cannot be judged because information is insufficient or contradictory, and cannot be supplemented or verified

5) Actions taken for IP in relation to AEs

① Drug interrupted: IP treatment is stopped.

② Dose reduced: IP dose or dosing frequency is decreased.

③ Dose increased: IP dose or dosing frequency is increased.

④ Dose not changed: IP dose is not changed.

⑤ Unknown: Lack of information on actions taken.

⑥ Not applicable: Not applicable to general methods of IP treatment specified in the protocol (i.e., Ae occurring prior to IP dosing, AE occurring during the follow-up period, death during the study period, etc.)

6) Treatment for AEs

① No action taken

② Drug therapy

③ Non-drug therapy

④ Drug and non-drug therapy

⑤ Unknown

7) Outcome of AEs

① Recovered

② Recovering

③ Not recovered

④ Recovered with sequelae

⑤ Fatal

⑥ Unknown

8) Seriousness of AEs

AEs that constitute 4) SAEs in '10.4.1 Definition of Adverse Events' will be recorded in the CRF.

① Results in death

② Is life-threatening

③ Requires inpatient hospitalization or prolongation of existing hospitalization

④ Results in persistent or significant disability/incapacity

⑤ Is a congenital anomaly/birth defect

⑥ Other important medical events

10.4.4. Actions to Be Taken for Adverse Events

1) Training for AEs

The PI will train sub-investigators and subjects or their representatives for all AEs that might occur after IP treatment and instruct them to report all phenomena occurring after IP use. Subjects who experience AEs will be managed to immediately receive necessary tests and treatment from the attending physician. In the event of SADRs, affected subjects will be withdrawn from the study, and appropriate actions will be taken promptly.

2) Follow-up of AEs

If possible, any unresolved AEs at the safety follow-up visit (Visit 7) will be followed up until resolution. To follow up AEs, the investigator or his or her designee will collect relevant information including status and date of resolution of AEs via a telephone contact (telephone visit) or an on-site visit.

3) Treatment for worsening of symptoms

If a subject meets criterion 7) listed in '5.5.2 Criteria for Premature Withdrawal', i.e., Continued study participation is considered inappropriate due to SAEs or SADRs which occurred during the study, IP will immediately be stopped and replaced with appropriate therapy to ensure the safety of the subject.

10.4.5. Reporting Procedures for Adverse Drug Reactions

During the study, the PI and the sub-investigator should make every effort to ensure the safety of subjects. In the event of an unexpected SADR, the PI and the sub-investigator should stop the study and promptly take appropriate actions in order to minimize the event.
In the event of an unexpected SADR during the study, each party involved is obliged to be as follows.

1) Obligations of the sponsor

① The sponsor should report all unexpected SADRs to the investigator, Minister of Food and Drug Safety, and if necessary, the IRB within the time frames defined below.

- Events resulting in death or life-threatening: Within 7 days from when the event is reported or known to the sponsor. If all information required by Attachment No. 77 ADR report form of Rules on Safety of Medicinal Products etc., such as term of ADR, final outcome, summary of ADR, etc., is not reported, the sponsor should submit an additional report containing detailed information on the ADR within 15 days from when the event is initially reported or known.
- Other unexpected SADRs: Within 15 days from when the event is reported or known to the sponsor.

② Any additional information obtained about the ADR reported according to ① should be reported by the sponsor until resolution of the event (i.e., disappearing of the event or lost to follow-up).

③ To report an ADR to Minister of Food and Drug Safety according to ①, the sponsor should submit a report using Attachment No. 77 ADR report form of Rules on Safety of Medicinal Products etc. with an attachment of ADR summary using CIOMS-I form, etc.

2) Obligations of the PI

① The PI should record all SAEs (with the exception of those for which immediate reporting is unnecessary as specified in the protocol or other documents such as IB) on the SAE page of eCRF within 1 (business) day from when the event is reported or known. In this case, the PI should use subject identification codes instead of subjects' personal information such as name, resident registration number, and address, etc. to protect their privacy. If applicable, the PI should comply with AE reporting guidelines provided by the sponsor.

Contact information for safety reporting

- email: PV@medisafe.kr
- Fax: 02-6915-8005

② AEs or laboratory test abnormalities, etc. that are specifically defined in the protocol as important in relation to safety evaluation should be reported by the PI to the sponsor according to the protocol-defined reporting methods within the protocol-defined time frames.

③ When reporting a case of death, the PI should submit to the sponsor and the IRB additional information such as autopsy reports (if performed) and terminal medical reports, etc.

3) Obligations of the sub-investigator

If an unexpected SAE, etc. occurs during the course of the study, the sub-investigator should immediately report the event to the PI.

4) Obligations of the IRB/Ethics Committee (EC)

In the event of an unexpected SADR, the IRB should have necessary actions taken via the PI, such as stopping all or part of the study.

10.5. Pregnancy

If a woman of childbearing potential becomes pregnant after being enrolled in the study and treated with IP, the investigator should notify the sponsor immediately after confirmed pregnancy is known, if possible, and should instruct the subject to discontinue IP treatment. If the female partner of a male subject becomes pregnant, the investigator should obtain consent for pregnancy information sharing from the female partner. To provide information on the study and IP, the subject information sheet may be provided for the female partner. If the female partner agrees to share pregnancy information, pregnancy reporting will proceed; Without her agreement, pregnancy related information cannot be collected.

The investigator should report the minimum information, including information on the pregnant woman and pregnancy progress information up to the point, etc., to the sponsor by telephone, email, or fax, etc. within 1 (calendar) day from when the subject's (or the subject's female partner's) pregnancy is known, and will complete and submit Pregnancy Reporting Form to the sponsor within 7 (calendar) days from when the pregnancy is known.

Regardless of EOS, all pregnancies should be followed up until the outcome of the pregnancy is known; Follow-up will be performed up to 4 weeks (28 days) after childbirth. If there is additional pregnancy information or major change, the investigator should complete and submit Pregnancy Reporting & Outcome Form to the sponsor according to the same method and time frame as in the initial reporting.

While pregnancy itself is not considered an AE, pregnancy complication, spontaneous abortion, ectopic pregnancy, stillbirth, neonatal death, and congenital anomaly, etc. should be recorded and followed up as AEs or SAEs (if the criteria for SAEs are met). Voluntary artificial abortion without complications that is not for medical reasons will not be considered an AE. In addition, hospitalization for natural birth of a healthy fetus will not be considered an SAE.

10.6. Overdose

Overdose is defined as accidental or intentional administration of a drug at a dose that is considered by the investigator to be excessive and medically significant. Regardless of outcome of the AE, the investigator will consider overdose an important medical event and report to the sponsor accordingly.

11. Data Analysis and Statistical Considerations

11.1. Analysis Sets

In this study, efficacy endpoints will be analyzed primarily in the full analysis (FA) set and supplementarily in the per-protocol (PP) set, and safety endpoints will be analyzed in the safety set according to actual treatment received. Demographics and other baseline characteristics will be analyzed in all randomized subjects.

11.1.1. Efficacy Sets

Full Analysis Set (FA set)
The FA set is defined as subjects who have at least one dose of IP after randomization and have at least one post-dose measurement of tear production by Schirmer's test. The intention-to-treat (ITT) principle applies.

Per-Protocol Set (PP set)
The PP set is defined as subjects in the FA set who have completed the study without major protocol violations. Criteria for exclusion from the PP set are as follows:

1) Violation of the inclusion and exclusion criteria
2) Premature withdrawal
3) Treatment with concomitant drugs or therapies
4) Treatment compliance < 80% or > 120% during the treatment period
5) Other major protocol violations

11.1.2. Safety Set

Safety set
The safety set is defined as subjects who have at least one dose of IP after randomization and have post-dose safety evaluation.

11.2. Statistical Analysis Methods

11.2.1. General Principles for Analysis

All statistical analyses will be performed using SAS® (version 9.4 or above, SAS Institute, Cary, NC, USA). Continuous data will be presented with number of subjects, mean, standard deviation, median, minimum, and maximum; and categorical data will be presented with frequency and percentage by category. Normality tests on continuous data will be performed using Shapiro-Wilk test. Unless otherwise specified, two-sided tests will be performed at a significance level of 5%.

11.2.2. Handling of Missing Values

In the FA set, any missing values for the efficacy endpoints will be imputed using the last observation carried forward (LOCF) method. In the PP set, any missing values for the efficacy and safety endpoints will not be imputed, and analyses will be performed only with available data.

11.2.3. Covariate Adjustment

For inter-group analyses of some of the efficacy endpoints including tear production (Schirmer's test), corneal staining score – Oxford grading, conjunctival staining score – Oxford grading, TBUT, DED questionnaire score (SPEED), and OSDI, analysis of covariance (ANCOVA) will be performed with adjustment for baseline and institution as covariates.

Proportion of subjects with a ≥ 10 mm/5 min increase or above from baseline in tear production (Schirmer's test) and total number of administered doses of rescue medication will be analyzed with adjustment for institution as a covariate.

11.2.4. Interim Analysis

In this study, no interim analysis will be performed.

11.2.5. Multiple Comparison or Multiplicity

Given the therapeutic exploratory nature of the Phase 2 study, adjustment for type 1 error by comparing each of the test groups (SCAI-001 eye drops 0.01%, 0.02%) with the comparator group is not considered.

11.2.6. Demographics and Other Baseline Characteristics

Demographics and other baseline characteristics of subjects who are included in the study will be summarized by treatment group. Continuous variables will be presented with number of subjects, mean, standard deviation, median, minimum, and maximum and compared between each test group and the comparator group (SCAI-001 eye drops 0.01% vs comparator group, SCAI-001 eye drops 0.02% vs comparator group) using two sample *t*-test (if normality assumption is satisfied) or Wilcoxon's rank sum test (if normality assumption is not satisfied) depending on satisfaction of normality assumption. Categorical variables will be presented with frequency and percentage and compared between each test group and the comparator group (SCAI-001 eye drops 0.01% vs comparator group, SCAI-001 eye drops 0.02% vs comparator group) using Pearson's chi-square test (if no more than 20% of cells have expected frequency less than 5) or Fisher's exact test (if more than 20% of cells have expected frequency less than 5) depending on whether more than 20% of cells have expected frequency less than 5.

Medical history will be standardized using Medical Dictionary for Regulatory Activities (MedDRA), and each standardized medical history will be presented with frequency, percentage, and number of events by group. Prior/concomitant medication will be standardized by ATC code of WHO Drug dictionary, and each standardized concomitant medication will be presented with frequency, percentage, and number of events by group.

11.2.7. Efficacy Evaluation

Efficacy will be analyzed using test results collected from the study eye.

The study eye will be the eye with the worse corneal staining results. If both eyes have the same corneal staining score, the study eye will be the eye with the worse Schirmer's test results without anesthesia. If both eyes have the same Schirmer's test results without anesthesia, the right eye (OD) will be the study eye.

11.2.7.1. Primary Efficacy Evaluation

For change from baseline in tear production (Schirmer's test) at Week 12, descriptive statistics (number of subjects, mean, standard deviation, median, minimum, and maximum) will be presented by treatment group. To test the difference between each test group and the comparator group (SCAI-001 eye drops 0.01% vs comparator group, SCAI-001 eye drops 0.02% vs comparator group), ANCOVA will be performed with adjustment for baseline and institution. ANCOVA results will be summarized with least squares mean (LSM) and standard error (SE) by treatment group, and LSM difference between the treatment groups and its 95% confidence interval (CI) with two-sided *p*-value.

11.2.7.2. Secondary Efficacy Evaluation

1) Change from baseline in tear production (Schirmer's test) at Week 4 and Week 8

For change from baseline in tear production (Schirmer's test) at Week 4 and Week 8, descriptive statistics (number of subjects, mean, standard deviation, median, minimum, and maximum) will be presented by treatment group. To test the difference between each test group and the comparator group (SCAI-001 eye drops 0.01% vs comparator group, SCAI-001 eye drops 0.02% vs comparator group), ANCOVA will be performed with adjustment for baseline and institution.

ANCOVA results will be summarized with LSM and SE by treatment group, and LSM difference between the treatment groups and its 95% CI with two-sided *p*-value.

2) Proportion of subjects with a ≥ 10 mm/5 min increase or above from baseline in tear production (Schirmer's test) at Week 4, Week 8, and Week 12

Proportion of subjects with a ≥ 10 mm/5 min increase or above from baseline in tear production (Schirmer's test) at Week 4, Week 8, and Week 12 and its exact 95% CI will be presented. To test the difference between each test group and the comparator group (SCAI-001 eye drops 0.01% vs comparator group, SCAI-001 eye drops 0.02% vs comparator group), Cochran-Mantel-Haenszel test will be performed with institution as a covariate.

3) Change from baseline in corneal staining score – Oxford grading at Week 4, Week 8, and Week 12

For change from baseline in corneal staining score – Oxford grading at Week 4, Week 8, and Week 12, descriptive statistics (number of subjects, mean, standard deviation, median, minimum, and maximum) will be presented by treatment group. To test the difference between each test group and the comparator group (SCAI-001 eye drops 0.01% vs comparator group, SCAI-001 eye drops 0.02% vs comparator group), ANCOVA will be performed with adjustment for baseline and institution. ANCOVA results will be summarized with LSM and SE by treatment group, and LSM difference between the treatment groups and its 95% CI with two-sided *p*-value.

4) Change from baseline in conjunctival staining score – Oxford grading at Week 4, Week 8, and Week 12

For change from baseline in conjunctival staining score – Oxford grading at Week 4, Week 8, and Week 12, descriptive statistics (number of subjects, mean, standard deviation, median, minimum, and maximum) will be presented by treatment group. To test the difference between each test group and the comparator group (SCAI-001 eye drops 0.01% vs comparator group, SCAI-001 eye drops 0.02% vs comparator group), ANCOVA will be performed with adjustment for baseline and institution. ANCOVA results will be summarized with LSM and SE by treatment group, and LSM difference between the treatment groups and its 95% CI with two-sided *p*-value.

5) Change from baseline in TBUT at Week 4, Week 8, and Week 12

TBUT will be analyzed based on the mean of 3 measurements. For change from baseline in TBUT at Week 4, Week 8, and Week 12, descriptive statistics (number of subjects, mean, standard deviation, median, minimum, and maximum) will be presented by treatment group. To test the difference between each test group and the comparator group (SCAI-001 eye drops 0.01% vs comparator group, SCAI-001 eye drops 0.02% vs comparator group), ANCOVA will be performed with adjustment for baseline and institution. ANCOVA results will be summarized with LSM and SE by treatment group, and LSM difference between the treatment groups and its 95% CI with two-sided *p*-value.

6) Change from baseline in DED questionnaire score (SPEED) at Week 4, Week 8, and Week 12

For change from baseline in DED questionnaire score (SPEED) at Week 4, Week 8, and Week 12, descriptive statistics (number of subjects, mean, standard deviation, median, minimum, and maximum) will be presented by treatment group. To test the difference between each test group and the comparator group (SCAI-001 eye drops 0.01% vs comparator group, SCAI-001 eye drops 0.02% vs comparator group), ANCOVA will be performed with adjustment for baseline and institution. ANCOVA results will be summarized with LSM and SE by treatment group, and LSM difference between the treatment groups and its 95% CI with two-sided *p*-value.

7) Change from baseline in OSDI at Week 4, Week 8, and Week 12

For change from baseline in OSDI at Week 4, Week 8, and Week 12, descriptive statistics (number of subjects, mean, standard deviation, median, minimum, and maximum) will be presented by treatment group. To test the difference between each test group and the comparator group (SCAI-001 eye drops 0.01% vs comparator group, SCAI-001 eye drops 0.02% vs comparator group), ANCOVA will be performed with adjustment for baseline and institution. ANCOVA results will be summarized with LSM and SE by treatment group, and LSM difference between the treatment groups and its 95% CI with two-sided *p*-value.

8) Total number of administered doses of rescue medication during the study

For total number of administered doses of rescue medication during the study, descriptive statistics (number of subjects, mean, standard deviation, median, minimum, and maximum) will be presented by treatment group. To test the difference between each test group and the comparator group (SCAI-001 eye drops 0.01% vs comparator group, SCAI-001 eye drops 0.02% vs comparator group), ANCOVA will be performed with adjustment for institution. ANCOVA results will be summarized with LSM and SE by treatment group, and LSM difference between the treatment groups and its 95% CI with two-sided *p*-value.

11.2.8. Safety Evaluation

11.2.8.1. Adverse Events

AEs will be analyzed based on TEAEs. For AEs, ADRs, SAEs, and SADRs, number of subjects, incidence and its 95% CI will be presented by treatment group. To test intra-group difference in the incidence of AEs, Pearson's chi-square test (if no more than 20% of cells have expected frequency less than 5) or Fisher's exact test (if more than 20% of cells have expected frequency less than 5) will be performed depending on whether more than 20% of cells have expected frequency less than 5. AEs, ADRs, SAEs, and SADRs will be coded by System Organ Class (SOC) and Preferred Term (PT) using MedDRA; For coded AEs, number of subjects, incidence, and number of events will be presented by treatment group. In addition, status of AEs by severity and by relationship to IP will also be presented.

11.2.8.2. Ocular Tolerance Test

For ocular tolerance test results, descriptive statistics (number of subjects, mean, standard deviation, median, minimum, and maximum) will be presented by treatment group for test values at each visit. To test the difference between each test group and the comparator group (SCAI-001 eye drops 0.01% vs comparator group, SCAI-001 eye drops 0.02% vs comparator group), two sample *t*-test (if normality assumption is satisfied) or Wilcoxon's rank sum test (if normality assumption is not satisfied) will be performed depending on satisfaction of normality assumption.

11.2.8.3. Best Corrected Visual Acuity

For best corrected visual acuity, descriptive statistics (number of subjects, mean, standard deviation, median, minimum, and maximum) will be presented by treatment group for measurements at each visit and for change from baseline. To test the intra-group difference before and after IP treatment, paired *t*-test (if normality assumption is satisfied) or Wilcoxon's signed-rank test (if normality assumption is not satisfied) will be performed depending on satisfaction of normality assumption. To test the difference in change from baseline between each test group and the comparator group (SCAI-001 eye drops 0.01% vs comparator group, SCAI-001 eye drops 0.02% vs comparator group), two sample *t*-test (if normality assumption is satisfied) or Wilcoxon's rank sum test (if normality assumption is not satisfied) will be performed depending on satisfaction of normality assumption.

11.2.8.4. Ophthalmologic Examination (Intraocular Pressure, Fundus Examination and Slit Lamp Microscopy, Ocular Hyperemia Assessment)

Ophthalmologic examination (IOP – mean of 2 measurements, fundus examination and slit lamp microscopy, ocular hyperemia assessment) results will be presented as follows. For continuous variables, descriptive statistics (number of subjects, mean, standard deviation, median, minimum, and maximum) will be presented by treatment group for measurements at each visit and for change from baseline. To test the intra-group difference before and after IP treatment, paired *t*-test (if normality assumption is satisfied) or Wilcoxon's signed-rank test (if normality assumption is not satisfied) will be performed depending on satisfaction of normality assumption. To test the difference in change from baseline between each test group and the comparator group (SCAI-001 eye drops 0.01% vs comparator group, SCAI-001 eye drops 0.02% vs comparator group), two sample *t*-test (if normality assumption is satisfied) or Wilcoxon's rank sum test (if normality assumption is not satisfied) will be performed depending on satisfaction of normality assumption. For categorical variables, change from baseline after IP treatment will be presented as a shift table by treatment group, and intra-group comparison will be performed using McNemar's test or Bowker's test.

11.2.8.5. Vital Signs

For vital signs, descriptive statistics (number of subjects, mean, standard deviation, median, minimum, and maximum) will be presented by treatment group for measurements at each visit and for change from baseline. To test the intra-group difference before and after IP treatment, paired *t*-test (if normality assumption is satisfied) or Wilcoxon's signed-rank test (if normality assumption is not satisfied) will be performed depending on satisfaction of normality assumption. To test the difference in change from baseline between each test group and the comparator group (SCAI-001 eye drops 0.01% vs comparator group, SCAI-001 eye drops 0.02% vs comparator group), two sample *t*-test (if normality assumption is satisfied) or Wilcoxon's rank sum test (if normality assumption is not satisfied) will be performed depending on satisfaction of normality assumption.

11.2.8.6. Laboratory Tests

Laboratory test results will be presented as follows. For continuous variables, descriptive statistics (number of subjects, mean, standard deviation, median, minimum, and maximum) will be presented by treatment group for test values at each visit and for change from baseline. To test the intra-group difference before and after IP treatment, paired *t*-test (if normality assumption is satisfied) or Wilcoxon's signed-rank test (if normality assumption is not satisfied) will be performed depending on satisfaction of normality assumption. To test the difference in change from baseline between each test group and the comparator group (SCAI-001 eye drops 0.01% vs comparator group, SCAI-001 eye drops 0.02% vs comparator group), two sample *t*-test (if normality assumption is satisfied) or Wilcoxon's rank sum test (if normality assumption is not satisfied) will be performed depending on satisfaction of normality assumption. For categorical variables, change from baseline after IP treatment will be presented as a shift table by treatment group, and intra-group comparison will be performed using McNemar's test or Bowker's test.

11.3. Rationale for Sample Size Determination

The objectives of the study are to demonstrate the efficacy and safety of SCAI-001 eye drops by comparing the test groups (SCAI-001 eye drops 0.01%, 0.02%) with the active comparator group (cyclosporine 0.05%), and to explore the doses of SCAI-001 eye drops in patients with DED. Therefore, the sample size was determined not based on statistical assumptions but based on the exploratory nature of the study. Considering 30 subjects per group plus a 20% drop-out rate for exploratory investigation, the study plans to recruit 38 subjects per treatment group.

12. Data Management

12.1. Data Collection/Access

Source documents are patient records generated by attending physicians and retained at the institution. Most source documents are medical charts generated by hospitals or attending physicians, in which case, all information collected and recorded in the patient's CRF should be consistent with that in the applicable chart. In this study, data will be collected in eCRF which can be accessed and corrected only by authorized individuals. The investigator should allow the sponsor, CRA, auditors, or inspectors to have direct access to relevant data including electronic documents, such as essential documents, etc.

12.2. Data Protection/Retention

The PI should retain relevant data including essential documents, etc. for 3 years from the date of approval of the study drug. However, the duration of data retention should be extended if instructed by the MFDS or determined necessary by the sponsor. In addition, upon the completion of the CSR, the relevant data should be transferred to an individual responsible for data retention who will be decided in advance by the PI in consultation with the sponsor. To retain various study related data and records in good condition, a separate place will be prepared and kept secure. The sponsor should inform the investigator in writing of justification and duration of data retention; If the sponsor determines that data retention is no longer necessary, the sponsor must notify this in writing to the PI.

13. Ethical Considerations and Administrative Procedures

13.1. Applicable Laws and Regulations

This study will be conducted in compliance with all national and international laws and regulations including the Declaration of Helsinki (1964) and Korean Good Clinical Practice (KGCP), study plans approved by the MFDS and the IRB of the PI's institution, and standard operating procedures (SOPs) of the CRO, CMIC Korea. Co., Ltd.

13.2. Informed Consent

Before a subject participates in the study or undergoes any specific, non-routine procedure involving risk, written informed consent should be obtained from the subject. Prior to study initiation, ICF will be submitted by the PI to applicable IRB for review and approval.
Prior to subject recruitment and enrollment, subjects will voluntarily provide ICF to study participation after being fully informed of details of the study, effects and AEs of IP, and safety information.
If a subject or his or her legally acceptable representative is unable to read ICF, subject information sheet, and other documented information, an impartial witness will be present in the entire consenting process. In this case, after all documented information is read and explained to the subject or his or her legally acceptable representative, the subject or his or her legally acceptable representative will provide verbal consent to study participation, and if possible, sign and date the ICF, and the impartial witness should confirm that, before the ICF was signed, the documented information was accurately explained to and understood by the subject or his or her legally acceptable representative and that the consenting process was voluntary on the part of the subject or his or her legally acceptable representative.
The original ICF will be signed both by the patient and by the PI (or the PI's designee), and a copy of the signed original ICF will be provided by the PI or the sub-investigator for the subject. The original ICF will be retained at the institution by the investigator.

13.3. Subject Compensation Rules

If damage is incurred to a subject due to the study, the sponsor will compensate the subject according to Subject Compensation Rules. For Subject Compensation Rules, see Attachment 3.

13.4. Criteria for Post-Study Medical Care and Treatment

Subjects who are prematurely withdrawn from the study will be advised to receive other appropriate treatment. Medical care and treatment for subjects who ended the study will be based on general guidelines for medical care and treatment in patients with DED.

13.5. Compliance with Ethical Principles and Measures to Protect Subjects

The study will be conducted in a scientific and ethical manner according to KGCP and other laws and regulations relevant to clinical trials. Based on the Declaration of Helsinki, the study will be conducted with respect for human dignity and human rights and will not cause any disadvantage to subjects.

- Institution

Head of the institution should ensure appropriate conduct of the study by having clinical laboratories, facilities, and professional human resources in place as required for study conduct and by ensuring that actions are taken for emergencies, etc.

- IRB

The IRB should be constituted according to national laws and regulations/conventions. It should protect the rights, safety, and wellbeing of subjects; If vulnerable subjects are to participate in the study, the IRB should thoroughly review its justification. If informed consent is not properly obtained, the study is not conducted per protocol, or an SAE or ADR is reported, the IRB should have necessary actions taken via the PI, such as stopping all or part of the study. In compliance with KGCP, the IRB should assess/approve the protocol and check on a regular basis if the study is being conducted per protocol.

- Investigator

Investigators refer to PIs, sub-investigators, and coordinating investigators. Before enrolling subjects in the study, the investigator should examine their health status to confirm their eligibility for study participation. In addition, the investigator will be fully familiar with the study drug and make every effort to ensure the safety of subjects.
If the investigator came across a subject's concurrent disease which requires medical treatment, the investigator should inform the subject. The investigator are required to accurately understand and analyze the protocol and actively respond to subjects' problems.

- Sponsor

A sponsor refers to an individual, company, institution, or organization that is responsible for planning, managing, and financing a clinical trial. The sponsor should ensure that subjects, study methods, and forms and entries of CRFs, etc. are implemented according to protocol-defined procedures. The sponsor's audit plans and procedures should be determined based on the importance of the study, sample size, type and complexity of study, extent of potential risk to subjects, and known problems in study conduct, etc.

Information on subjects and their medical records obtained from the study will be shared only by the PI, sub-investigators, and the CRO and may be provided only upon the request of the IRB and competent authorities such as the MFDS.
If a subject experiences an AE due to the study, appropriate medical care will be provided until the subject recovers, and the injury caused by IP will be compensated according to Subject Compensation Rules.

13.5.1. Approval and Amendment of Study Plans

According to Article 24, Paragraph 1 of 「Rules on Safety of Medicinal Products etc.」, study plans will be submitted to the MFDS, and decisions made by the MFDS and the IRB on study conduct will be notified in writing to the investigator and the sponsor prior to study initiation. The protocol, ICF, advertisements for subject recruitment, and other written study information to be provided for subjects (or their legally acceptable representatives) must be approved by the MFDS and/or the IRB, and the relevant documents must be retained by the investigator and the sponsor.
The PI or the sub-investigator is responsible for continuously being reviewed for the study on a regular basis, at least annually or as specified by the IRB.
The PI and the sponsor should report study progress, SAEs, life-threatening problems, or deaths to the MFDS and/or the IRB and inform the MFDS and the IRB of EOS.

The protocol should be amended upon mutual agreement between the investigator and the sponsor. Protocol amendments must be approved by the MFDS and the IRB before they are implemented. However, according to Article 10, Paragraph 2 of 「Regulations on Approval of Clinical Trial Plans, etc.」, protocol amendments that are unlikely to have a significant impact on the safety of subjects or the reliability of study results may be implemented upon approval by the IRB, without the MFDS' approval. Administrative changes generally do not require the IRB's approval, but relevant information should be notified to the IRB.
Amendments related to the safety of subjects may be implemented without approval by the MFDS and the IRB if that is necessary to remove imminent harm from a subject, in which case, the investigator must submit the amendment to the MFDS and the IRB for approval within a predefined time frame after the treatment.

13.5.2. Protocol Violation

To remove imminent risk from a subject, the PI or the sub-investigator may violate or amend the protocol without the IRB's prior approval. In this case, the violation or amendment implemented and its justification as well as any proposed protocol amendment should be submitted to the IRB for review and approval, and if necessary, to regulatory authorities, as soon as possible after the fact.
The PI will report to the IRB any major protocol violations that may affect the safety and integrity of the study and other violations that are considered important, etc.

13.6. Publication

All data and results generated during the study are the properties of the sponsor, and upon EOS, a CSR for this study will be prepared by a medical writer of the sponsor or the CRO authorized by the sponsor. The CSR will be prepared in consultation with the sponsor and the PI. Publication of the CSR should be agreed upon, in advance, by and between the sponsor and the institution. In addition, the sponsor has the right to present study results at any time.

In terms of study registration, the sponsor will register study information on freely available websites for public access (e.g., www.clinicaltrials.gov, etc.).
On this website, basic information on each study is registered to provide sufficient information to subjects (or their health managers) who are interested in the respective study about how to be enrolled in the study. To ensure the transparency of study results, clinical trials are registered with a unique identifier, and clinical trial information is to be presented within 21 days after the first subject's enrollment.

13.7. Confidentiality of Patient Records

All laboratory samples, assessment forms, reports, and other records will be handled in a manner to maintain subject confidentiality, and all records will be retained in a secure storage area with limited access. All study related documents including CRFs, etc. will be documented and identified not with subjects' names but with their identification codes. Study related information will not be disclosed without the subject's written consent, except to the extent that it is necessary for monitoring and auditing by regulatory authorities or the IRB.
The PI or the sub-investigator will not disclose or use any of the study related records and other unpublished confidential information for any purpose other than the study conduct.
When the study results are published, subject identities will be kept in confidence.

13.8. Quality Control and Quality Assurance

13.8.1. Study Monitoring

CRA is divided into blinded and unblinded CRA. Besides maintaining necessary contacts by telephone calls and letters, the blinded CRA will visit the PI or the sub-investigator at each institution on a regular basis to monitor and discuss study progress. The CRA will maintain up-to-date knowledge about the study by reviewing observations and study records, directly accessing source documents, and discussing study conduct with the PI or the sub-investigator and relevant staff.
The unblinded CRA will review IP use and relevant documents such as IP accountability logs by monitoring the pharmacy.
In addition, the CRA will carefully monitor all aspects of the study to ensure that the study complies with applicable government regulations reflected in the latest KGCP and SOPs.
CRFs will be verified by the CRA for completeness, validity, and consistency, and any queries will be resolved by the investigator.

13.8.2. Audits and Inspections

To ensure the safety of subjects and to obtain accurate, complete, and reliable data, the investigator should retain clinical test results, clinical records, and subject's medical records as source documents. The PI or the sub-investigators and the institution that participate in the study should allow study related monitoring, audits, IRB's reviews, and regulatory authorities' inspections. The investigator should allow direct access to study related data upon the request of the MFDS and the EC.

14. Information on Sponsor and Names and Positions of Principal Investigators

14.1. Names and Addresses of Institutions

For the list of institutions, see Attachment 1. List of Institutions.

14.2. Principal Investigators and Other Study Participants

For information on coordinating investigators, principal investigators, and other study participants, see Attachment 1. List of Institutions.

14.3. Sponsor

Sponsor: SCAI Therapeutics
Address: 2nd Floor, 21, Gangnamdae-ro 107-gil, Seocho-gu, Seoul (Daeneung Building, Jamwon-dong)

14.4. Contract Research Organization

CRO: CMIC Korea. Co., Ltd.
Address: 10th Floor, Gangnam N Tower, 129, Teheran-ro, Gangnam-gu, Seoul

15. Others Required for Safe and Scientific Study Conduct

In an effort to minimize study violations, an investigator meeting will be held prior to study initiation to review relevant documents including the protocol and to invite input from investigators. In addition, an interim investigator meeting may be held if deemed necessary by the sponsor or the investigator to adjust issues between the institutions or address violations, etc.

<Matters required for study initiation>
Prior to IP shipping to the institution and prior to study initiation, the followings are required:

1) A copy of the approved protocol and (if necessary) a copy of the approved protocol amendment
2) Curricula vitae of PI and sub-investigators
3) Written approvals of study plans by the MFDS and the IRB
4) A copy of subject information sheet and ICF
5) Normal ranges of clinical laboratory tests
6) Other documents required by the government

Subjects must not be enrolled before an official meeting is held between the investigator and the sponsor for study initiation, and the meeting agenda should include reviewing the protocol and the CRF.

16. References

1. Nelson JD, et al. TFOS DEWS II Introduction. *Ocul Surf.* 2017;15(3):269-275.
2. Craig JP, et al. TFOS DEWS II Definition and Classification Report. *Ocul Surf.* 2017;15(3):276-283.
3. Farrand KF, et al. Prevalence of Diagnosed Dry Eye Disease in the United States Among Adults Aged 18 Years and Older. *Am J Ophthalmol*. 2017;182:90-98.
4. Health Insurance Review & Assessment Service, National Health Insurance Corporation. 2019 National Health Insurance Statistical Yearbook. Approved by National Statistical Office, No.920006. ISSN 1738-8945. October 2020.
5. Cho, Mi-Kyoung, et al. "Factors Influencing the Cognitive Degree of Dry Eyes in Nursing Students." *Perspectives in Nursing Science* 13.2 (2016): 110-118.
6. Jung, Hyun Ho, et al. "Clinical efficacy of topical 3% diquafosol tetrasodium in short tear film break-up time dry eye." *Journal of the Korean Ophthalmological Society* 56.3 (2015): 339-344.
7. Özcura, Fatih, Sayime Aydin, and Mehmet Rami Helvaci. "Ocular surface disease index for the diagnosis of dry eye syndrome." *Ocular immunology and inflammation* 15.5 (2007): 389-393.
8. Leonardi, Andrea, et al. "Efficacy and safety of 0.1% cyclosporine A cationic emulsion in the treatment of severe dry eye disease: a multicenter randomized trial." *European journal of ophthalmology* 26.4 (2016): 287-296.
9. Messmer, Elisabeth M. "The pathophysiology, diagnosis, and treatment of dry eye disease." *Deutsches Ärzteblatt International* 112.5 (2015): 71.
10. Bartlett, Jimmy D., et al. "Associations between signs and symptoms of dry eye disease: a systematic review." *Clinical ophthalmology (Auckland, NZ)* 9 (2015): 1719.
11. Yang, Soonwon, et al. "Comparisons for evaluation of efficacy and safety of cyclosporin a 0.05% ophthalmic emulsion treatment groups." *Journal of the Korean Ophthalmological Society* 57.12 (2016): 1849-1856.
12. Foulks GN, Bron AJ. Meibomian gland dysfunction: a clinical scheme for description, diagnosis, classification, and grading. *Ocul Surf.* 2003;1(3):107-126.
13. Uchino M. What We Know About the Epidemiology of Dry Eye Disease in Japan. *Invest Ophthalmol Vis Sci.* 2018;59(14):DES1-DES6.
14. Mehra D, Galor A. Digital Screen Use and Dry Eye: A Review. *Asia Pac J Ophthalmol (Phila).* 2020;9(6):491-497.
15. Koh S, Rhee MK. COVID-19 and Dry Eye. *Eye Contact Lens.* 2021;47(6):317-322.
16. Salinas-Toro D, et al. High frequency of digital eye strain and dry eye disease in teleworkers during the coronavirus disease (2019) pandemic [published online ahead of print, 2021 Jul 7]. *Int J Occup Saf Ergon.* 2021;1-6.
17. Hyon, Joon Young, et al. "Korean guidelines for the diagnosis and management of dry eye: development and validation of clinical efficacy." *Korean Journal of Ophthalmology* 28.3 (2014): 197-206.
18. Phadatare, Suvarna P., et al. "A comprehensive review on dry eye disease: diagnosis, medical management, recent developments, and future challenges." *Advances in Pharmaceutics* 2015 (2015).
19. The International Dry Eye Workshop. "The definition and classification of dry eye disease: report of the Definition and Classification Subcommittee of the International Dry Eye WorkShop." *Ocul Surf.* 5(2):75-92. 2007.
20. Tedesco, Dustin, and Lukas Haragsim. "Cyclosporine: a review." *Journal of transplantation* 2012 (2012).
21. Label of Restasis® 0.05% in South Korea
22. Babamohamadi, Hassan, et al. "Comparing vitamin A and moist chamber in preventing ocular surface disorders." *Clinical nursing research* 27.6 (2018): 714-729.
23. Nathan Efron, BScOptom, et al. "Contact Lens Complications, 3rd Edition" (2012)
