# Supplementary material for: Comparative study on the clinical effectiveness of low-concentration cyclosporine eye drops in patients with dry eye disease
Source: PLoS One. 2026 Sep 28;21(9):e0358007. doi: 10.1371/journal.pone.0358007 (PMC13618915; doi:10.1371/journal.pone.0358007)
Supplement: Supplementary file 2 [file pone.0358007.s001.docx]

**S1 Appendix. Exclusion criteria for the randomized controlled trial**

1. Patients with clinically significant ocular diseases not caused by dry eye (e.g., corneal surface disorders, abnormal corneal sensitivity)

2. Individuals with a history of systemic steroid or immunosuppressive agent use (e.g., azathioprine, tacrolimus, cyclosporine, mycophenolic acid) within the last 90 days before screening.

3. Individuals with a history of cyclosporine eye drop use within the last 6 weeks before screening.

4. Individuals with a history of using anti-inflammatory agents for dry eye disease, other than cyclosporine eye drops, within the last 30 days before screening.

5. Individuals with a history of taking other medications within the last 30 days before screening that may affect dry eye disease.

6. Those who have worn contact lenses within 72 hours before screening or are required to wear them during the clinical trial period.

7. Those with a history of eye surgery within the past 12 months before screening.

8. Those who have undergone procedures such as punctal plug insertion, punctal occlusion surgery, punctal dilation surgery, or ophthalmic laser procedures within the last 90 days before screening.

9. Those who have undergone vision correction surgeries such as LASIK or PRK within the last 12 months before screening.

10. Individuals with active symptoms of eye infections such as active allergies, anterior uveitis, or Stevens–Johnson syndrome.

11. Patients with Sjögren's syndrome or other autoimmune diseases.

12. Those with a history of corneal transplantation or neurotrophic keratopathy.

13. Individuals with a history of hepatitis B or C.

14. Those with a history of malignant tumors within the past 5 years before screening.

15. Uncontrolled hypertension (>160/100 mmHg), insulin-dependent diabetes, or HbA1c >8% at screening.

16. Individuals diagnosed with intraocular pressure exceeding 21 mmHg in one or both eyes or diagnosed with glaucoma.

17. Those anticipated to require ophthalmic procedures or surgery during the clinical trial period as determined by the investigator.

18. Participants with laboratory test results outside the normal range, specifically creatinine levels exceeding two times the upper limit of normal or ALT/AST levels exceeding two times the upper limit of normal.

19. Individuals with hypersensitivity reactions to the components of the investigational drug.

20. Individuals with mental disorders.

21. Pregnant or breastfeeding women.

22. Women with pregnancy plans or those with positive pregnancy test results at screening.

23. Those with a history on abusing medications or alcohol.

24. Those have received other investigational drugs 30 days before screening.

25. Those who are inappropriate for participating in this study according to investigator's judgement.
