# Supplementary material for: Comparative study on the clinical effectiveness of low-concentration cyclosporine eye drops in patients with dry eye disease
Source: PLoS One. 2026 Sep 28;21(9):e0358007. doi: 10.1371/journal.pone.0358007 (PMC13618915; doi:10.1371/journal.pone.0358007)
Supplement: Supplementary file 3 [file pone.0358007.s002.docx]

**S2 Appendix. Reason for withdrawal by treatment group, comparison of baseline characteristics between study completers and non-completers, and sensitivity analysis of the primary efficacy endpoint using a mixed-effects model for repeated measures.**

**Table 1. Reason for withdrawal by treatment group.**

| **Reason for withdrawal** | **SCAI-001 0.01% (N=40)** | **SCAI-001 0.02% (N=38)** | **Restasis (control) (N=38)** |
| --- | --- | --- | --- |
| Consent withdrawal | 3 | 1 | 0 |
| Adverse event | 1 | 0 | 0 |
| Inclusion/exclusion criteria deviation | 0 | 2 | 0 |
| Use of prohibited medication | 0 | 1 | 0 |
| **Total** | **4** | **4** | **0** |

**Table 2. Baseline demographic and clinical characteristics of study completers versus non-completers**

| **Characteristic** | **Completers (N=108)** | **Non-completers (N=8)** | **P-value**  **(Welch's t-test)** | **P-value (Wilcoxon rank-sum test)** |
| --- | --- | --- | --- | --- |
| Sex, male, n (%) | 10 (9.26) | 1 (12.50) | 0.5611* | 0.5611* |
| Sex, female, n (%) | 98 (90.74) | 7 (87.50) |  |  |
| Age (years) |  |  | 0.9565 | 0.9826 |
| n | 108 | 8 |  |  |
| Mean ± SD | 44.54 ± 12.91 | 44.88 ± 16.58 |  |  |
| Median | 42.00 | 46.50 |  |  |
| Min–Max | 23.00–71.00 | 24.00–70.00 |  |  |
| Baseline Schirmer's test score |  |  | 0.0909 | 0.1129 |
| n | 108 | 7 |  |  |
| Mean ± SD | 5.58 ± 2.34 | 4.14 ± 1.86 |  |  |
| Median | 5.50 | 5.00 |  |  |
| Min–Max | 1.00–10.00 | 2.00–7.00 |  |  |
| Baseline corneal staining score |  |  | 0.2416 | 0.0514 |
| n | 108 | 7 |  |  |
| Mean ± SD | 2.17 ± 0.40 | 2.71 ± 1.11 |  |  |
| Median | 2.00 | 2.00 |  |  |
| Min–Max | 2.00–4.00 | 2.00–5.00 |  |  |
| Baseline OSDI |  |  | 0.4589 | 0.4504 |
| n | 108 | 7 |  |  |
| Mean ± SD | 37.23 ± 20.09 | 42.73 ± 17.87 |  |  |
| Median | 38.05 | 41.70 |  |  |
| Min–Max | 0.00–81.80 | 18.20–70.00 |  |  |

*SD, standard deviation; OSDI, Ocular Surface Disease Index. Given the marked imbalance in group size between completers and non-completers, Welch's t-test was used as the primary comparison for continuous variables, and the Wilcoxon rank-sum test was performed as a sensitivity analysis; * denotes Fisher's exact test, used for the categorical variable sex.*

**Table 3. Sensitivity analysis of the change in tear volume (Schirmer's test) from baseline using a mixed-effects model for repeated measures (MMRM) (Full Analysis Set).**

| **Visit** | **n, 0.01%** | **n, 0.02%** | **n, control** | **LSM diff 0.01% vs control [95% CI], P** | **LSM diff 0.02% vs control [95% CI], P** |
| --- | --- | --- | --- | --- | --- |
| Week 4 | 40 | 37 | 38 | 1.34 [-0.59, 3.27], P=0.1700 | 1.04 [-0.99, 3.07], P=0.3107 |
| Week 8 | 38 | 35 | 38 | -0.67 [-2.94, 1.60], P=0.5591 | -0.41 [-2.77, 1.96], P=0.7350 |
| Week 12 | 36 | 34 | 38 | 0.82 [-1.74, 3.37], P=0.5285 | 1.29 [-1.36, 3.93], P=0.3376 |

*LSM, least squares mean; CI, confidence interval. The MMRM included treatment, visit, and treatment-by-visit interaction as fixed effects, and site and baseline value as covariates; the model uses all available observed data at each visit and does not carry forward missing values. Between-group comparisons from the MMRM were consistent with those from the primary ANCOVA/LOCF-based analysis at every visit, indicating that the study conclusions are robust to the method used to handle missing data.*
