# Supplementary material for: Comparative study on the clinical effectiveness of low-concentration cyclosporine eye drops in patients with dry eye disease
Source: PLoS One. 2026 Sep 28;21(9):e0358007. doi: 10.1371/journal.pone.0358007 (PMC13618915; doi:10.1371/journal.pone.0358007)
Supplement: Supplementary file 4 [file pone.0358007.s003.docx]

**S3 Appendix. Sensitivity analysis of change in tear volume by Schirmer’s test from baseline using a mixed model for repeated measures (MMRM)**

*As a post-hoc sensitivity analysis for the primary efficacy endpoint, change in tear volume from baseline was additionally analyzed using a mixed model for repeated measures (MMRM), with treatment, visit, and treatment-by-visit interaction as fixed effects and clinical trial site and baseline value as covariates. This analysis was performed to assess whether the between-group comparisons based on the pre-specified analysis of covariance (ANCOVA; Section 11.2.7 of the Statistical Analysis Plan in Protocol) were robust to a model that additionally accounts for the within-subject correlation across repeated visits. Results below are presented for the Full Analysis Set (FAS).*

**S3 Appendix Table. Change from baseline in tear volume by Schirmer’s test at 4, 8, and 12 weeks (FAS)**

| **Visit** | **Statistic** | **SCAI-001 0.01%**  **(N=40)** | **SCAI-001 0.02%**  **(N=37)** | **Control (Restasis)**  **(N=38)** |
| --- | --- | --- | --- | --- |
| Baseline | n | 40 | 37 | 38 |
|  | Mean±SD | 5.48±2.43 | 5.89±2.20 | 5.13±2.35 |
|  | Median | 5.00 | 6.00 | 5.00 |
|  | Min–Max | 1.00–10.00 | 2.00–10.00 | 1.00–9.00 |
|  |  |  |  |  |
| Week 4 | n | 40 | 37 | 38 |
|  | Mean±SD | 7.68±6.16 | 8.11±4.72 | 5.82±3.62 |
|  | Median | 6.50 | 7.00 | 5.00 |
|  | Min–Max | 1.00–35.00 | 1.00–22.00 | 1.00–15.00 |
|  |  |  |  |  |
| **Change from baseline to Week 4** | n | 40 | 37 | 38 |
|  | Mean±SD | 2.20±5.93 | 2.22±3.77 | 0.68±3.20 |
|  | LSM±SE | 2.19±0.72 | 1.89±0.79 | 0.84±0.79 |
|  | Median | 1.00 | 1.00 | 0.00 |
|  | Min–Max | −6.00–30.00 | −3.00–13.00 | −5.00–11.00 |
|  | P-value¹ | 0.0081* | 0.0010* | 0.2997 |
|  |  |  |  |  |
|  | LSM difference (Test−Control)±SE | 1.34±0.97 | 1.04±1.02 | – |
|  | 95% CI of LSM difference | −0.59, 3.27 | −0.99, 3.07 | – |
|  | P-value² | 0.1700 | 0.3107 | – |
|  |  |  |  |  |
| Week 8 | n | 38 | 35 | 38 |
|  | Mean±SD | 7.55±6.84 | 8.46±4.79 | 7.50±4.39 |
|  | Median | 6.00 | 8.00 | 7.00 |
|  | Min–Max | 2.00–35.00 | 2.00–20.00 | 1.00–20.00 |
|  |  |  |  |  |
| **Change from baseline to Week 8** | n | 38 | 35 | 38 |
|  | Mean±SD | 1.92±6.70 | 2.43±4.12 | 2.37±4.08 |
|  | LSM±SE | 1.86±0.84 | 2.12±0.91 | 2.53±0.89 |
|  | Median | 1.00 | 2.00 | 1.50 |
|  | Min–Max | −7.00–29.00 | −3.00–13.00 | −5.00–16.00 |
|  | P-value¹ | 0.1572 | 0.0014* | 0.0002* |
|  |  |  |  |  |
|  | LSM difference (Test−Control)±SE | −0.67±1.14 | −0.41±1.19 | – |
|  | 95% CI of LSM difference | −2.94, 1.60 | −2.77, 1.96 | – |
|  | P-value² | 0.5591 | 0.7350 | – |
|  |  |  |  |  |
| Week 12 | n | 36 | 34 | 38 |
|  | Mean±SD | 8.64±5.93 | 9.85±7.42 | 7.26±4.37 |
|  | Median | 7.00 | 7.50 | 7.00 |
|  | Min–Max | 2.00–25.00 | 1.00–34.00 | 2.00–22.00 |
|  |  |  |  |  |
| **Change from baseline to Week 12 (primary endpoint)** | n | 36 | 34 | 38 |
|  | Mean±SD | 2.97±5.62 | 3.85±7.12 | 2.13±4.52 |
|  | LSM±SE | 3.11±0.97 | 3.58±1.01 | 2.29±0.98 |
|  | Median | 1.00 | 2.50 | 1.00 |
|  | Min–Max | −6.00–16.00 | −4.00–26.00 | −4.00–18.00 |
|  | P-value¹ | 0.0034* | 0.0029* | 0.0075* |
|  |  |  |  |  |
|  | LSM difference (Test−Control)±SE | 0.82±1.29 | 1.29±1.34 | – |
|  | 95% CI of LSM difference | −1.74, 3.37 | −1.36, 3.93 | – |
|  | P-value² | 0.5285 | 0.3376 | – |

CI: confidence interval; FAS: full analysis set; LSM: least squares mean; SD: standard deviation; SE: standard error.

¹ Wilcoxon’s signed-rank test (within-group change from baseline).

² Mixed model for repeated measures (MMRM), with treatment, visit, and treatment-by-visit interaction as fixed effects, and clinical trial site and baseline value as covariates. LSM difference = LSM of the SCAI-001 group − LSM of the control group.

* Statistically significant (p<0.05).
