# Supplementary material for: Comparative study on the clinical effectiveness of low-concentration cyclosporine eye drops in patients with dry eye disease
Source: PLoS One. 2026 Sep 28;21(9):e0358007. doi: 10.1371/journal.pone.0358007 (PMC13618915; doi:10.1371/journal.pone.0358007)
Supplement: Supplementary file 5 [file pone.0358007.s004.docx]

**S4 Appendix. Baseline values and change from baseline for secondary efficacy endpoints by visit and treatment group.**

|  | **SCAI-001 0.01% (N=40)** | **P-value** | **SCAI-001 0.02% (N=37)** | **P-value** | **Control (Restasis) (N=38)** | **P-value** |
| --- | --- | --- | --- | --- | --- | --- |
| **Corneal stain** | | | | | | |
| Baseline | 2.23±0.58 |  | 2.19±0.40 |  | 2.18±0.46 |  |
| 4 weeks | 1.20±0.99 |  | 1.22±0.92 |  | 1.39±0.89 |  |
| Change from baseline | -1.03±0.86 | <0.0001* | -0.97±0.83 | <0.0001* | -0.79±0.96 | <0.0001* |
| 8 weeks | 0.93±0.89 |  | 0.89±0.70 |  | 1.13±0.81 |  |
| Change from baseline | -1.30±0.69 | <0.0001* | -1.30±0.70 | <0.0001* | -1.05±0.87 | <0.0001* |
| 12 weeks | 0.85±0.89 |  | 0.65±0.79 |  | 0.71±0.77 |  |
| Change from baseline | -1.38±0.67 | <0.0001* | -1.54±0.73 | <0.0001* | -1.47±0.73 | <0.0001* |
| **Conjunctival stain** | | | | | | |
| Baseline | 2.53±1.40 |  | 2.68±1.40 |  | 2.32±1.76 |  |
| 4 weeks | 1.33±1.25 |  | 1.92±1.46 |  | 2.08±1.62 |  |
| Change from baseline | -1.20±1.16 | <0.0001* | -0.76±1.53 | 0.0082* | -0.24±1.73 | 0.4160 |
| 8 weeks | 1.10±1.08 |  | 1.35±1.23 |  | 1.55±1.41 |  |
| Change from baseline | -1.43±1.39 | <0.0001* | -1.32±1.36 | <0.0001* | -0.76±1.87 | 0.0241* |
| 12 weeks | 1.00±1.06 |  | 1.05±1.13 |  | 0.97±1.08 |  |
| Change from baseline | -1.53±1.20 | <0.0001* | -1.62±1.26 | <0.0001* | -1.34±1.60 | <0.0001* |
|  | | | | | | |
| **TBUT** | | | | | | |
| Baseline | 3.79±1.31 |  | 3.57±1.18 |  | 3.59±1.32 |  |
| 4 weeks | 5.54±3.85 |  | 4.90±2.18 |  | 4.81±3.04 |  |
| Change from baseline | 1.74±4.04 | 0.0177* | 1.33±2.06 | 0.0001† | 1.22±2.97 | 0.0015* |
| 8 weeks | 5.94±2.81 |  | 5.38±2.89 |  | 5.15±2.27 |  |
| Change from baseline | 2.15±2.87 | <0.0001* | 1.81±2.99 | <0.0001* | 1.56±2.52 | 0.0005† |
| 12 weeks | 6.11±3.51 |  | 6.00±2.91 |  | 6.11±2.26 |  |
| Change from baseline | 2.32±3.47 | <0.0001* | 2.43±2.90 | <0.0001* | 2.52±2.47 | <0.0001† |
| **SPEED** | | | | | | |
| Baseline | 11.85±5.39 |  | 12.65±6.05 |  | 11.03±6.05 |  |
| 4 weeks | 7.95±5.66 |  | 7.30±4.81 |  | 8.18±5.85 |  |
| Change from baseline | -3.90±4.67 | <0.0001† | -5.35±6.31 | <0.0001† | -2.84±5.64 | 0.0036† |
| 8 weeks | 7.45±6.18 |  | 6.19±4.40 |  | 6.05±4.93 |  |
| Change from baseline | -4.40±5.81 | <0.0001* | -6.46±6.14 | <0.0001* | -4.97±5.57 | <0.0001* |
| 12 weeks | 6.70±5.36 |  | 5.08±4.82 |  | 5.58±5.73 |  |
| Change from baseline | -5.15±5.47 | <0.0001* | -7.57±6.01 | <0.0001* | -5.45±6.27 | <0.0001* |
| **OSDI** | | | | | | |
| Baseline | 38.41±16.72 |  | 39.78±20.41 |  | 34.51±22.56 |  |
| 4 weeks | 26.21±19.41 |  | 20.22±14.45 |  | 20.32±17.23 |  |
| Change from baseline | -12.20±18.66 | <0.0001* | -19.56±16.93 | <0.0001* | -14.19±22.44 | <0.0001* |
| 8 weeks | 24.38±18.69 |  | 16.94±12.23 |  | 17.82±15.14 |  |
| Change from baseline | -14.03±19.93 | <0.0001* | -22.84±18.89 | <0.0001† | -16.69±19.03 | <0.0001* |
| 12 weeks | 24.67±19.84 |  | 16.66±12.49 |  | 15.99±17.12 |  |
| Change from baseline | -13.74±22.08 | 0.0001* | -23.12±18.31 | <0.0001† | -18.52±20.01 | <0.0001* |

* Wilcoxon’s signed-rank test, †Paired t-test. TBUT, tear film break-up time; SPEED, Standard Patient Evaluation of Eye Dryness; OSDI, Ocular Surface Disease Index; Data are presented as mean ± standard deviation. P-values indicate the within-group comparison of each post-baseline value versus baseline
