# Supplementary material for: Comparative study on the clinical effectiveness of low-concentration cyclosporine eye drops in patients with dry eye disease
Source: PLoS One. 2026 Sep 28;21(9):e0358007. doi: 10.1371/journal.pone.0358007 (PMC13618915; doi:10.1371/journal.pone.0358007)
Supplement: Supplementary file 7 [file pone.0358007.s006.docx]

**CONSORT 2025 Flow Diagram**

Flow diagram of the progress through the phases of a randomised trial of two groups (that is, enrolment, intervention allocation, follow-up, and data analysis)

Allocated to Control (n=38)

Received allocated intervention (n=38)

Did not receive allocated intervention (n=0)

Allocated to SCAI-001 0.02% (n=38)

Received allocated intervention (n=37)

Did not receive allocated intervention

(n=1)

Not meeting inclusion criteria (n=1)

Allocated to SCAI-001 0.01% (n=40)

Received allocated intervention (n=40)

Did not receive allocated intervention (n=0)

Completed intervention (n=36)

Discontinued intervention (n=4)

Consent withdrawal (n=3)

Adverse event (n=1)

Completed intervention (n=38)

Completed intervention (n=34)

Discontinued intervention (n=4)

Not meeting inclusion criteria (n=2)

Consent withdrawal (n=1)

Protocol deviation (n=1)

Excluded (n=12)

Not meeting inclusion criteria (n=10)

Declined to participate (n=2)

Analysis

Analysed for primary outcome (n=38)

Excluded from analysis (n=0)

Analysed for primary outcome (n=40)

Excluded from analysis (n=0)

Analysed for primary outcome (n=37)

Excluded from analysis (n=1)

Did not receive intervention (n=1)

Allocation

Assessed for eligibility (n=128)

Enrolment

Randomised (n=116)
