# Supplementary material for: Comparative study on the clinical effectiveness of low-concentration cyclosporine eye drops in patients with dry eye disease
Source: PLoS One. 2026 Sep 28;21(9):e0358007. doi: 10.1371/journal.pone.0358007 (PMC13618915; doi:10.1371/journal.pone.0358007)
Supplement: Supplementary file 8 [file pone.0358007.s010.docx]

**Reviewer #1:**

1. **A sample size calculation is critical for this study design.**

: As it is a Phase 2 study as specified in the study protocol (Section 11.3), the sample size was determined not based on statistical power calculation but based on the exploratory nature of the study. Considering 30 subjects per group plus a 20% drop-out rate for exploratory investigation, the study plans to recruit 38 subjects per treatment group. We have revised the Methods section of the manuscript to state this rationale (page 8, line 122).

2. **For a longitudinal study, a linear mixed model or a generalized estimating equations (GEE) approach is recommended. Both within-group and between-group comparisons can be performed in a single model. GEE can accommodate non-normal outcomes, and baseline values can be adjusted as covariates. Linear mixed/GEE models also handle data that are missing at random reasonably well. T-tests use less data with reducing statistical power. Consult a statistician especially for the primary analysis.**

: We thank the reviewer for this comment. We would like to clarify that between-group comparisons for all efficacy outcomes were performed using analysis of covariance (ANCOVA) adjusting for baseline value and clinical trial site — not t-tests as you may have inferred. We have revised the Statistical Analysis subsection to state this explicitly (page 13, line 253). In addition, as suggested, we performed a post-hoc sensitivity analysis of the primary endpoint (change in tear volume) using a mixed model for repeated measures (MMRM), incorporating treatment, visit, and treatment-by-visit interaction as fixed effects and site and baseline value as covariates. The MMRM results were consistent with the pre-specified ANCOVA findings of comparable efficacy between SCAI-001 and Restasis. The MMRM results are presented in **S3 Appendix and added to the revised manuscript (page 15, line 286).**

3. **Present a table showing baseline values and change from baseline at each time point, at least for the primary outcome. P-values may need adjustment for secondary outcomes.**

: We have added a new table (Table 2) presenting baseline values, values at each post-baseline visit (Weeks 4, 8, and 12), and the corresponding change from baseline for the primary endpoint (Schirmer's test) in each treatment group. Values for all secondary endpoints (corneal staining, conjunctival staining, TBUT, SPEED, and OSDI) are now provided in S4 Appendix. As this was a Phase 2 exploratory study, no adjustment for multiplicity was pre-specified for secondary endpoints (Protocol Section 11.2.5), and p-values for these outcomes should be interpreted as exploratory (page 14, line 258).

4. **What was the block size used for randomization?**
   : Participants were randomized in a 1:1:1 ratio with variable block size of 6 and 9. It is added to Study protocol subsection (page 9, line 161).
5. **Table 1 should assess balance across all three groups, not only treatment vs control. There is no need to label the statistical method in the p-value column; instead, specify the methods in the Statistical Analysis section (e.g., continuous variables were compared using t-tests and categorical variables using chi-square tests).**
   : We revised Table 1 to compare baseline characteristics across all three treatment groups simultaneously and moved the statistical test labels from the p-value column to the Statistical Analysis section, as suggested.
6. **In Figure 1, “Safety” should replace “Saftey.” Replace “Safety Set” with “FAS,” and omit “Excluded from the FAS n=0.” There is no need to show safety set in the flow diagram.**
   : We have revised Figure 1 accordingly.
7. **In addition, the exclusion criteria for the PPS are unclear. For example, is use of a contraindicated drug considered a compliance issue or a protocol deviation/violation also?**
   We clarify that compliance is defined by dosing adherence rate, and is therefore distinct from contraindicated drug use. The apparent overlap arose because "Protocol deviation/violation" in Figure 1 was missing the word "Other," implying it could include contraindicated drug use as well. We have corrected this to "Other protocol deviation/violation" to clarify that these are separate, non-overlapping exclusion categories.
8. **Clarify the reasons for dropouts (e.g., lack of efficacy vs safety-related reasons).**

: Of the 8 dropouts, one was attributable to a drug-related adverse event; four were due to voluntary withdrawal of consent; three were due to protocol-related reasons—including use of a prohibited concomitant medication (antihistamine), prior use of cyclosporine eye drops within the screening exclusion window found out after randomization, and incomplete screening laboratory tests—none related to lack of efficacy. **This clarification has been added to the S2 Appendix.**

**Reviewer #2:** The manuscript entitled “Comparative study on the clinical effectiveness of low-concentration cyclosporine eye drops in patients with dry eye syndrome” addresses a clinically relevant question regarding whether lower-concentration nanoparticle-based cyclosporine formulations can provide comparable efficacy and tolerability to conventional cyclosporine 0.05% emulsion in dry eye syndrome. The multicenter, randomized, double-blind design is a strength, and the topic is potentially valuable. However, the present manuscript requires several important corrections before it can be considered acceptable. I recommend minor revision, provided that the authors address the following points rigorously.
**1. First, the trial-registration timing must be clarified transparently. The manuscript states that recruitment began on 4 January 2023 and that ClinicalTrials.gov registration was first posted on 17 February 2023. This raises concern that the trial may have been registered after participant recruitment had already started. The authors must explicitly state whether registration was prospective or retrospective, whether the protocol was finalized before enrollment, and whether any outcomes were changed after enrollment began.**

: This study was conducted as an MFDS-authorized clinical trial in Korea. Prior to enrollment of the first participant, the study protocol, informed consent form, and related study documents were reviewed and approved by both the Ministry of Food and Drug Safety (MFDS) and the Institutional Review Board (IRB). The study objectives, eligibility criteria, endpoints, and statistical analysis plan were finalized before participant enrollment commenced.

Although the ClinicalTrials.gov record was first posted on 17 February 2023, after enrollment had begun on 4 January 2023, registration on ClinicalTrials.gov was not a regulatory requirement for conduct of this trial in Korea. The study was registered to enhance transparency and public accessibility of trial information.

Importantly, no changes were made to the study objectives, primary endpoint, secondary endpoints, eligibility criteria, or statistical analysis plan after participant enrollment began. The timing of the ClinicalTrials.gov posting did not reflect any modification of the study design or outcome measures. This clarification has been added to the Study design section of the revised manuscript (page 8, line 132).

**2. Second, the interpretation of efficacy is too favorable relative to the actual primary endpoint. The primary endpoint was change in tear volume at 12 weeks, and although all groups improved from baseline, there were no significant intergroup differences. Therefore, the conclusion should not imply superiority of SCAI-001. The authors should state clearly that SCAI-001 showed comparable efficacy to Restasis, while the earlier 4-week tear-volume improvement was exploratory and not supported by significant between-group difference.**
We thank the reviewer for this important observation. We have revised the Abstract and Discussion to state clearly that SCAI-001 demonstrated efficacy comparable to Restasis, and that the earlier tear-volume improvement observed at 4 weeks was a secondary, exploratory finding not supported by a statistically significant between-group difference (page 19, line 360), (page 23, line 452).

**3. Third, the statistical analysis requires clearer correction for multiplicity. The manuscript reports many secondary outcomes across multiple time points, including Schirmer’s test, corneal staining, conjunctival staining, TBUT, SPEED, and OSDI at 4, 8, and 12 weeks. Without adjustment for multiple comparisons, isolated findings such as better conjunctival staining with SCAI-001 0.01% at weeks 4 and 8 may be false-positive. The authors should clarify whether multiplicity adjustment was planned or explicitly label these findings exploratory.**
As this was an exploratory Phase 2 trial, no multiplicity adjustment was applied across secondary endpoints and time points, per the pre-specified statistical analysis plan (Protocol Section 11.2.5). All secondary endpoint findings should therefore be interpreted as exploratory rather than confirmatory. This clarification has been added to Statistical analysis section of the revised manuscript (page 14, line 256).

**4. Fourth, the handling of missing data and attrition requires more detailed justification. Of 116 randomized participants, only 96 completed the study and were included in the per-protocol set. The authors used last observation carried forward for missing efficacy endpoints. This approach can bias treatment-effect estimation in dry eye trials with fluctuating symptoms. The authors should provide reasons for withdrawal by group, compare baseline features of completers and non-completers, and add sensitivity analyses using available-case or mixed-model methods.**
: We thank the reviewer for this comment. Reasons for withdrawal, baseline comparisons between completers and non-completers, and a sensitivity analysis using available cases are now provided in S2 Appendix. No significant baseline differences were observed between completers and non-completers, and the sensitivity analysis showed no meaningful differences from the primary analysis (page 14, line 270).

**5. Fifth, the conflict-of-interest and funding statements require careful revision. The submission form states that SCAI Therapeutics supported the study and had a role in study design, data collection, and analysis, yet the competing-interest statement says that no competing interests exist. This is not sufficiently transparent. The authors should explicitly disclose sponsor involvement in trial design, product supply, data management, statistical analysis, manuscript preparation, and publication decisions, and clarify whether any authors received payment, consulting support, or institutional funding.**
: We thank the reviewer for pointing out this inconsistency between the funding disclosure and the competing-interest statement. It is revised as follows.

- Competing Interests : The authors declare no personal competing interests. No authors received personal payments, or other financial compensation from SCAI Therapeutics in relation to this study.

- Funding statement : SCAI Therapeutics funded the study and provided the investigational product. Research funding was provided to the participating institutions to support study conduct in accordance with the clinical trial agreement. The sponsor participated in study design, data collection oversight, data management, and statistical analysis. However, SCAI Therapeutics did not participate in manuscript drafting or preparation. The authors retained responsibility for data interpretation and the decision to submit the manuscript for publication.

Changes in competing interests is provided in the comment field, as the "Competing Interests" section of the submission form was not editable.

**6. Sixth, the manuscript would benefit from more comprehensive contextualization within the current dry eye disease treatment landscape. Several relevant systematic reviews and meta-analyses addressing topical therapy, ocular surface management, and adjunctive dry eye interventions should be considered to strengthen the Introduction and Discussion. The following listed 4 studies are very helpful to consider for citation: 1. PMID: 40679590; DOI: 10.1007/s00210-025-04454-8, because it directly compares topical ophthalmic drugs for keratoconjunctivitis sicca and is highly relevant to positioning cyclosporine among current pharmacologic options; 2. PMID: 40393913; DOI: 10.1016/j.clae.2025.102438, because punctal plugs are a commonly used non-pharmacologic strategy and provide broader context for tear-volume improvement in dry eye disease; 3. PMID: 40352434; DOI: 10.1177/25158414251338775, because thermal pulsation therapy is relevant to meibomian gland dysfunction-associated dry eye and helps contextualize non-immunomodulatory treatment approaches; and 4. PMID: 40204900; DOI: 10.1038/s41433-025-03790-6, because botulinum toxin A represents an emerging dry eye intervention and broadens the discussion of current and developing therapeutic strategies.**
: We thank the reviewer for this valuable suggestion. We have added a citation to the Introduction (page 6, line 85).

Overall, this manuscript addresses an important therapeutic question and provides useful randomized clinical data. However, the authors must correct the trial-registration transparency issue, moderate efficacy claims, address multiplicity, clarify attrition and missing-data handling, and improve disclosure of sponsor involvement. After these minor but necessary revisions, the manuscript can be accepted for publication.

**Reviewer #3:**

1. **Missing data on baseline dry eye severity. Please supplement this part of the results. Baseline imbalance could confound results.**

: We have added new tables (Table 2 and S4 appendix) presenting baseline values.

2. **In this Clinical Trial, the primary efficacy endpoint was the change in tear volume after 12 weeks. While, as we all known, the Schirmer test, with poor reproducibility and high stimulation effect, has a high coefficient of variation, is of limited clinical relevance compared to objective signs like corneal staining and BUT. Please explain the reason for using tear volume as the primary efficacy endpoint.**

: We appreciate the reviewer's insightful comment regarding the choice of primary efficacy endpoint. We acknowledge that Schirmer's test is known to have relatively high inter-individual and intra-individual variability.

However, tear volume was selected as the primary endpoint for the following reasons. First, this design followed regulatory and academic precedent set by cyclosporine-class ophthalmic formulations. Restasis® (cyclosporine 0.05%) was approved by FDA in 2003 based on a statistically significant improvement in tear production measured by Schirmer’s test (De Oliveira et al., Clin Ophthalmol 2019). This same endpoint design was subsequently adopted as the sole primary efficacy endpoint in the phase 3 trial of OTX-101 (cyclosporine ophthalmic solution 0.09%, approved by the FDA in 2018 as Cequa®), in which a significantly greater proportion of eyes achieved improvement in Schirmer's test score (Goldberg et al., Ophthalmology 2019).

Second, SCAI-001 was developed with the primary mechanistic goal of enhancing corneal penetration and normalizing sensory nerve signaling to the lacrimal gland, thereby increasing quantitative tear secretion; tear volume change is therefore the endpoint most directly aligned with this proposed mechanism of action.

We have added a statement regarding this point as limitations in discussion section and noting it as a consideration for future trial design (page 22, line 440).
**3. As authors has mentioned in the introduction, SCAI eye drops (0.01% and 0.02% cyclosporine) with lower concentrations, aiming to alleviate discomfort such as ocular irritation and foreign body sensation caused by the formulation of conventional cyclosporine eye drop such as 0.05% cyclosporine (Restasis, Allergan Inc.). While, safety outcomes showed that in the ocular tolerability evaluation, no differences were determined in the severity scores of the seven symptoms between the study and control groups. Does this result deviate from the main purpose of the innovative low‑concentration cyclosporine eye drops?**

: We thank the reviewer for this insightful and important comment. We would like to note that this was a phase 2 clinical trial, which is generally intended to provide preliminary evidence on dose-response relationships and efficacy signals to help inform the design of subsequent phase 3 studies, and may not always be structured to yield a confirmatory, adequately powered comparison of safety and tolerability outcomes between groups. In this context, the sample size for the present study was primarily determined based on the primary efficacy endpoint (change in Schirmer's test score at 12 weeks), and it is possible that the study was not sufficiently powered to detect more subtle between-group differences in ocular tolerability; as such, we believe the absence of statistically significant differences in symptom severity scores may not necessarily indicate that the tolerability profiles are entirely equivalent. We agree that this is an important point, and further phase 3 studies incorporating tolerability-specific endpoints and appropriate power calculations may help to more definitively characterize this aspect in the future. We have accordingly added a statement addressing this point as a limitation in the Discussion section (page 21 line 416).

**Reviewer #4:** This study is good one in the field of dry eye management. There are some issues in introduction, methodology and discussion. This study has problems in language, sentence construction and some repetition. Best wishes!
**Line 63: please mention mechanism of action in detail.**

-> We have added a detailed description of cyclosporine's mechanism of action and its downstream effects on the ocular surface to the Introduction, with supporting references (page 5, line 56, 66). We have also revised the terminology throughout the manuscript, replacing "dry eye syndrome (DES)" with "dry eye disease (DED)," as the latter is now more widely used and consistently adopted in the current literature.

**Line 119: What sampling technique was used?**

-> We have used consecutive sampling during recruitment. We have added the sampling technique in the revised manuscript (page 8, line 138).
**Line 119:** **This study included adult men and women aged 19 years who were diagnosed to have DES. (need to change the present line with this one)**

🡪 We have revised the sentence as suggested by the reviewer (page 8, line 138).
**Line 122: Why is visual acuity significant in case of dry eye syndrome?**
-> The visual acuity criterion was applied to exclude participants with visually significant ocular comorbidities that could confound the assessment of dry eye–related outcomes. In addition, visual acuity was monitored throughout the study as a safety measure to detect any blurred vision associated with study medication use. We have added a sentence to the Study Population section clarifying the reason (page 9, line 145).

**Line 131: Why was washout period of 2 week considered?**

-> A 2-week washout period was implemented to eliminate the residual pharmacologic effect of any prior dry eye treatment (e.g., artificial tears, anti-inflammatory eye drops) before the baseline assessment, ensuring that eligibility criteria and baseline severity reflected the participant's untreated disease state rather than a treatment-influenced state. This duration is consistent with washout periods commonly adopted in similar dry eye disease trials evaluating topical cyclosporine formulations, and was considered sufficient given the short residual action of the prior topical agents typically used by this population, while minimizing excessive discomfort or dropout risk from prolonging the untreated period. We have added this rationale to the Methods section (page 9, line 155).

**Line 135: How have you differentiated treatment group and control group as restasis is not a placebo treatment?**

-> All study medications were identical in packaging to maintain blinding as stated in the study protocol section (page 9, line 166).
**Line 160: How have you addressed reflex lacrimation?**

-> Tear volume was assessed using the unanesthetized Schirmer's test which reflects both basal and reflex-stimulated tear secretion. Rather than a confound, we interpret this reflex component mechanistically: as noted in the Discussion, improved Schirmer's outcomes across all groups likely reflect reduced ocular surface inflammation, restored corneal epithelial integrity, and normalized sensory signaling to the lacrimal gland, consistent with prior reports linking Schirmer I improvement to recovery of reflex tearing tied to lacrimal gland function (page 19, line 364). However, we acknowledge, as a limitation, the inherent variability of Schirmer's testing, and have added a statement on this point in the Discussion (page 22, line 440).

**Line 199: Restructure the sentence.**
-> We have revised the sentence (page 12, line 223).

**Line 209: Does this mean the patients with only one post-drug follow up were also included in the analysis?**

-> Yes, under the intention-to-treat principle, the full analysis set includes participants with only one post-dose Schirmer's test measurement, and for those who discontinued early, the last observation carried forward (LOCF) method was used to impute subsequent time points. To address the potential bias this may introduce, we performed an additional sensitivity analysis using a mixed-effects model for repeated measures (MMRM), which uses all available observed data at each visit without carrying forward missing values. This analysis is provided in S2 Appendix, and the results were consistent with the primary analysis.

**Line 285: Restructure the sentence.**
-> We have revised the sentence (page 18, line 331).

**Line 291: Discussion portion lack comparison of the results with similar study.**

-> We thank the reviewer for this comment. We have added a paragraph to the Discussion comparing our findings with previous studies on nanotechnology-based cyclosporine formulations. These studies support the premise that nanosizing technology can preserve therapeutic efficacy while allowing a reduction in concentration or dosing frequency, consistent with the results of the present study (page 20, line 395).

We have also expanded the Discussion to compare our findings with similar studies, noting that SCAI-001's lower surfactant content relative to Restasis may explain the conjunctival staining advantage, supported by evidence of surfactant-related conjunctival cytotoxicity and by CyclASol, a surfactant-free formulation that similarly outperformed Restasis on conjunctival staining. (page 20, line 389)

**Line 292: This first paragraph of discussion seems similar to the introduction. So kindly omit repetition. Please add conclusion.**

-> We thank the reviewer for this comment. We have removed the mechanistic background sentences in the first paragraph of the Discussion and revised the remaining text to concisely summarize the study result (page 18, line 339).
