# Supplementary material for: Comparative study on the clinical effectiveness of low-concentration cyclosporine eye drops in patients with dry eye disease
Source: PLoS One. 2026 Sep 28;21(9):e0358007. doi: 10.1371/journal.pone.0358007 (PMC13618915; doi:10.1371/journal.pone.0358007)
Supplement: Peer review history

# Peer review: Decision Letter 0

**DOI:** 10.1371/journal.pone.0358007.r001

**Authors:** Kofi Asiedu

7 Jun 2026

Dear Dr. Lee,

Thank you for submitting your manuscript to PLOS ONE. After careful consideration, we feel that it has merit but does not fully meet PLOS ONE’s publication criteria as it currently stands. Therefore, we invite you to submit a revised version of the manuscript that addresses the points raised during the review process.

==============================

For Lab, Study and Registered Report Protocols: These article types are not expected to include results but may include pilot data.

==============================

Please submit your revised manuscript by Jul 22 2026 11:59PM. If you will need more time than this to complete your revisions, please reply to this message or contact the journal office at plosone@plos.org. When you're ready to submit your revision, log on to https://www.editorialmanager.com/pone/ and select the 'Submissions Needing Revision' folder to locate your manuscript file.

- A letter that responds to each point raised by the academic editor and reviewer(s). You should upload this letter as a separate file labeled 'Response to Reviewers'.
- A marked-up copy of your manuscript that highlights changes made to the original version. You should upload this as a separate file labeled 'Revised Manuscript with Track Changes'.
- An unmarked version of your revised paper without tracked changes. You should upload this as a separate file labeled 'Manuscript'.

If you would like to make changes to your financial disclosure, please include your updated statement in your cover letter. Guidelines for resubmitting your figure files are available below the reviewer comments at the end of this letter.

If applicable, we recommend that you deposit your laboratory protocols in protocols.io to enhance the reproducibility of your results. Protocols.io assigns your protocol its own identifier (DOI) so that it can be cited independently in the future. For instructions see: https://journals.plos.org/plosone/s/submission-guidelines#loc-laboratory-protocols. Additionally, PLOS ONE offers an option for publishing peer-reviewed Lab Protocol articles, which describe protocols hosted on protocols.io. Read more information on sharing protocols at https://plos.org/protocols?utm_medium=editorial-email&utm_source=authorletters&utm_campaign=protocols.

As the corresponding author, your ORCID iD is verified in the submission system and will appear in the published article. PLOS supports the use of ORCID, and we encourage all coauthors to register for an ORCID iD and use it as well. Please encourage your coauthors to verify their ORCID iD within the submission system before final acceptance, as unverified ORCID iDs will not appear in the published article. *Only*  the individual author can complete the verification step; PLOS staff *cannot*  verify ORCID iDs on behalf of authors.

We look forward to receiving your revised manuscript.

Kind regards,

Kofi Asiedu, O.D., Ph.D. FAAO

Academic Editor

PLOS One

Journal Requirements:

When submitting your revision, we need you to address these additional requirements.

1. Please ensure that your manuscript meets PLOS ONE's style requirements, including those for file naming. The PLOS ONE style templates can be found at

https://journals.plos.org/plosone/s/file?id=wjVg/PLOSOne_formatting_sample_main_body.pdf and

https://journals.plos.org/plosone/s/file?id=ba62/PLOSOne_formatting_sample_title_authors_affiliations.pdf

2. Please upload a new copy of Figure 8 as the detail is not clear. Please follow the link for more information:  https://journals.plos.org/plosone/s/figures

3. Original Protocol Contains Confidentiality Notice.

4. Original Protocol Contains Logo.

5. Please include captions for your Supporting Information files at the end of your manuscript, and update any in-text citations to match accordingly. Please see our Supporting Information guidelines for more information: http://journals.plos.org/plosone/s/supporting-information.

6. Thank you for providing your underlying data as Supporting Information.

We note that the data set contains text or data that is not in English. Please note that PLOS is an English-language publisher, so we require data sets to be provided in English as well. Please upload an English-language version of your data set.

This will also allow us to determine if your data follows PLOS standards per our Data Availability policy here: https://journals.plos.org/plosone/s/data-availability

7. We note that there is identifying data in the Supporting Information file “Supporting information.zip”. Due to the inclusion of these potentially identifying data, we have removed this file from your file inventory. Prior to sharing human research participant data, authors should consult with an ethics committee to ensure data are shared in accordance with participant consent and all applicable local laws.

Data sharing should never compromise participant privacy. It is therefore not appropriate to publicly share personally identifiable data on human research participants. The following are examples of data that should not be shared:

-Name, initials, physical address

-Ages more specific than whole numbers

-Internet protocol (IP) address

-Specific dates (birth dates, death dates, examination dates, etc.)

-Contact information such as phone number or email address

-Location data

-ID numbers that seem specific (long numbers, include initials, titled “Hospital ID”) rather than random (small numbers in numerical order)

Data that are not directly identifying may also be inappropriate to share, as in combination they can become identifying. For example, data collected from a small group of participants, vulnerable populations, or private groups should not be shared if they involve indirect identifiers (such as sex, ethnicity, location, etc.) that may risk the identification of study participants.

Additional guidance on preparing raw data for publication can be found in our Data Policy (https://journals.plos.org/plosone/s/data-availability#loc-human-research-participant-data-and-other-sensitive-data) and in the following article: http://www.bmj.com/content/340/bmj.c181.long.

Please remove or anonymize all personal information “ID”, ensure that the data shared are in accordance with participant consent, and re-upload a fully anonymized data set. Please note that spreadsheet columns with personal information must be removed and not hidden as all hidden columns will appear in the published file.

8. If the reviewer comments include a recommendation to cite specific previously published works, please review and evaluate these publications to determine whether they are relevant and should be cited. There is no requirement to cite these works unless the editor has indicated otherwise.

9. Please review your reference list to ensure that it is complete and correct. If you have cited papers that have been retracted, please include the rationale for doing so in the manuscript text, or remove these references and replace them with relevant current references. Any changes to the reference list should be mentioned in the rebuttal letter that accompanies your revised manuscript. If you need to cite a retracted article, indicate the article’s retracted status in the References list and also include a citation and full reference for the retraction notice.

[Note: HTML markup is below. Please do not edit.]

Reviewers' comments:

Reviewer's Responses to Questions

**Comments to the Author**

1. Is the manuscript technically sound, and do the data support the conclusions?

Reviewer #1: Partly

Reviewer #2: Yes

Reviewer #3: Partly

Reviewer #4: Partly

**********

2. Has the statistical analysis been performed appropriately and rigorously?

Reviewer #1: No

Reviewer #2: Yes

Reviewer #3: Yes

Reviewer #4: Yes

**********

3. Have the authors made all data underlying the findings in their manuscript fully available?

The PLOS Data policy

Reviewer #1: No

Reviewer #2: Yes

Reviewer #3: No

Reviewer #4: Yes

**********

4. Is the manuscript presented in an intelligible fashion and written in standard English?

Reviewer #1: No

Reviewer #2: Yes

Reviewer #3: Yes

Reviewer #4: No

**********

Reviewer #1: A sample size calculation is critical for this study design.

For a longitudinal study, a linear mixed model or a generalized estimating equations (GEE) approach is recommended. Both within-group and between-group comparisons can be performed in a single model. GEE can accommodate non-normal outcomes, and baseline values can be adjusted as covariates. Linear mixed/GEE models also handle data that are missing at random reasonably well. T-tests use less data with reducing statistical power. Consult a statistician especially for the primary analysis.

Present a table showing baseline values and change from baseline at each time point, at least for the primary outcome. P-values may need adjustment for secondary outcomes.

What was the block size used for randomization?

Table 1 should assess balance across all three groups, not only treatment vs control. There is no need to label the statistical method in the p-value column; instead, specify the methods in the Statistical Analysis section (e.g., continuous variables were compared using t-tests and categorical variables using chi-square tests).

In Figure 1, “Safety” should replace “Saftey.” Replace “Safety Set” with “FAS,” and omit “Excluded from the FAS n=0.” There is no need to show safety set in the flow diagram.

In addition, the exclusion criteria for the PPS are unclear. For example, is use of a contraindicated drug considered a compliance issue or a protocol deviation/violation also?

Clarify the reasons for dropouts (e.g., lack of efficacy vs safety-related reasons).

Reviewer #2: The manuscript entitled “Comparative study on the clinical effectiveness of low-concentration cyclosporine eye drops in patients with dry eye syndrome” addresses a clinically relevant question regarding whether lower-concentration nanoparticle-based cyclosporine formulations can provide comparable efficacy and tolerability to conventional cyclosporine 0.05% emulsion in dry eye syndrome. The multicenter, randomized, double-blind design is a strength, and the topic is potentially valuable. However, the present manuscript requires several important corrections before it can be considered acceptable. I recommend minor revision, provided that the authors address the following points rigorously.

First, the trial-registration timing must be clarified transparently. The manuscript states that recruitment began on 4 January 2023 and that ClinicalTrials.gov registration was first posted on 17 February 2023. This raises concern that the trial may have been registered after participant recruitment had already started. The authors must explicitly state whether registration was prospective or retrospective, whether the protocol was finalized before enrollment, and whether any outcomes were changed after enrollment began.

Second, the interpretation of efficacy is too favorable relative to the actual primary endpoint. The primary endpoint was change in tear volume at 12 weeks, and although all groups improved from baseline, there were no significant intergroup differences. Therefore, the conclusion should not imply superiority of SCAI-001. The authors should state clearly that SCAI-001 showed comparable efficacy to Restasis, while the earlier 4-week tear-volume improvement was exploratory and not supported by significant between-group difference.

Third, the statistical analysis requires clearer correction for multiplicity. The manuscript reports many secondary outcomes across multiple time points, including Schirmer’s test, corneal staining, conjunctival staining, TBUT, SPEED, and OSDI at 4, 8, and 12 weeks. Without adjustment for multiple comparisons, isolated findings such as better conjunctival staining with SCAI-001 0.01% at weeks 4 and 8 may be false-positive. The authors should clarify whether multiplicity adjustment was planned or explicitly label these findings exploratory.

Fourth, the handling of missing data and attrition requires more detailed justification. Of 116 randomized participants, only 96 completed the study and were included in the per-protocol set. The authors used last observation carried forward for missing efficacy endpoints. This approach can bias treatment-effect estimation in dry eye trials with fluctuating symptoms. The authors should provide reasons for withdrawal by group, compare baseline features of completers and non-completers, and add sensitivity analyses using available-case or mixed-model methods.

Fifth, the conflict-of-interest and funding statements require careful revision. The submission form states that SCAI Therapeutics supported the study and had a role in study design, data collection, and analysis, yet the competing-interest statement says that no competing interests exist. This is not sufficiently transparent. The authors should explicitly disclose sponsor involvement in trial design, product supply, data management, statistical analysis, manuscript preparation, and publication decisions, and clarify whether any authors received payment, consulting support, or institutional funding.

Sixth, the manuscript would benefit from more comprehensive contextualization within the current dry eye disease treatment landscape. Several relevant systematic reviews and meta-analyses addressing topical therapy, ocular surface management, and adjunctive dry eye interventions should be considered to strengthen the Introduction and Discussion. The following listed 4 studies are very helpful to consider for citation: 1. PMID: 40679590; DOI: 10.1007/s00210-025-04454-8, because it directly compares topical ophthalmic drugs for keratoconjunctivitis sicca and is highly relevant to positioning cyclosporine among current pharmacologic options; 2. PMID: 40393913; DOI: 10.1016/j.clae.2025.102438, because punctal plugs are a commonly used non-pharmacologic strategy and provide broader context for tear-volume improvement in dry eye disease; 3. PMID: 40352434; DOI: 10.1177/25158414251338775, because thermal pulsation therapy is relevant to meibomian gland dysfunction-associated dry eye and helps contextualize non-immunomodulatory treatment approaches; and 4. PMID: 40204900; DOI: 10.1038/s41433-025-03790-6, because botulinum toxin A represents an emerging dry eye intervention and broadens the discussion of current and developing therapeutic strategies.

Overall, this manuscript addresses an important therapeutic question and provides useful randomized clinical data. However, the authors must correct the trial-registration transparency issue, moderate efficacy claims, address multiplicity, clarify attrition and missing-data handling, and improve disclosure of sponsor involvement. After these minor but necessary revisions, the manuscript can be accepted for publication.

Reviewer #3: 1. Missing data on baseline dry eye severity. Please supplement this part of the results. Baseline imbalance could confound results.

2. In this Clinical Trial, the primary efficacy endpoint was the change in tear volume after 12 weeks. While, as we all known, the Schirmer test, with poor reproducibility and high stimulation effect, has a high coefficient of variation, is of limited clinical relevance compared to objective signs like corneal staining and BUT. Please explain the reason for using tear volume as the primary efficacy endpoint.

3. As authors has mentioned in the introduction, SCAI eye drops (0.01% and 0.02% cyclosporine) with lower concentrations, aiming to alleviate discomfort such as ocular irritation and foreign body sensation caused by the formulation of conventional cyclosporine eye drop such as 0.05% cyclosporine (Restasis, Allergan Inc.). While, safety outcomes showed that in theocular tolerability evaluation, no differences were determined in the severity scores of the sev-en symptoms between the study and control groups. Does this result deviate from the mai-n purpose of the innovative low‑concentration cyclosporine eye drops?

Reviewer #4: This study is good one in the field of dry eye management. There are some issues in introduction, methodology and discussion. This study has problems in language, sentence construction and some repetition. Best wishes!

Line 63: please mention mechanism of action in detail.

Line 119: What sampling technique was used?

Line 119: This study included adult men and women aged 19 years who were diagnosed to have DES. (need to change the present line with this one)

Line 122: Why is visual acuity significant in case of dry eye syndrome?

Line 131: Why was washout period of 2 week considered?

Line 135: How have you differentiated treatment group and control group as restasis is not a placebo treatment?

Line 160: How have you addressed reflex lacrimation?

Line 199: Restructure the sentence.

Line 209: Does this mean the patients with only one post-drug follow up were also included in the analysis?

Line 285: Restructure the sentence.

Line 291: Discussion portion lack comparison of the results with similar study.

Line 292: This first paragraph of discussion seems similar to the introduction. So kindly omt repetition.

Please add conclusion.

**********

what does this mean?). If published, this will include your full peer review and any attached files.

If you choose “no”, your identity will remain anonymous but your review may still be made public.

**Do you want your identity to be public for this peer review?**  For information about this choice, including consent withdrawal, please see our Privacy Policy

Reviewer #1: No

Reviewer #2: No

Reviewer #3: No

Reviewer #4: **Yes:** Raju Kaiti

**********

[NOTE: If reviewer comments were submitted as an attachment file, they will be attached to this email and accessible via the submission site. Please log into your account, locate the manuscript record, and check for the action link "View Attachments". If this link does not appear, there are no attachment files.]

To ensure your figures meet our technical requirements, please review our figure guidelines: https://journals.plos.org/plosone/s/figures

You may also use PLOS’s free figure tool, NAAS, to help you prepare publication quality figures: https://journals.plos.org/plosone/s/figures#loc-tools-for-figure-preparation.

NAAS will assess whether your figures meet our technical requirements by comparing each figure against our figure specifications.

# Author comment: Author response to Decision Letter 1

**DOI:** 10.1371/journal.pone.0358007.r002

5 Aug 2026

Reviewer #1:

1. A sample size calculation is critical for this study design.

: As it is a Phase 2 study as specified in the study protocol (Section 11.3), the sample size was determined not based on statistical power calculation but based on the exploratory nature of the study. Considering 30 subjects per group plus a 20% drop-out rate for exploratory investigation, the study plans to recruit 38 subjects per treatment group. We have revised the Methods section of the manuscript to state this rationale (page 8, line 122).

2. For a longitudinal study, a linear mixed model or a generalized estimating equations (GEE) approach is recommended. Both within-group and between-group comparisons can be performed in a single model. GEE can accommodate non-normal outcomes, and baseline values can be adjusted as covariates. Linear mixed/GEE models also handle data that are missing at random reasonably well. T-tests use less data with reducing statistical power. Consult a statistician especially for the primary analysis.

: We thank the reviewer for this comment. We would like to clarify that between-group comparisons for all efficacy outcomes were performed using analysis of covariance (ANCOVA) adjusting for baseline value and clinical trial site — not t-tests as you may have inferred. We have revised the Statistical Analysis subsection to state this explicitly (page 13, line 253). In addition, as suggested, we performed a post-hoc sensitivity analysis of the primary endpoint (change in tear volume) using a mixed model for repeated measures (MMRM), incorporating treatment, visit, and treatment-by-visit interaction as fixed effects and site and baseline value as covariates. The MMRM results were consistent with the pre-specified ANCOVA findings of comparable efficacy between SCAI-001 and Restasis. The MMRM results are presented in S3 Appendix and added to the revised manuscript (page 15, line 286).

3. Present a table showing baseline values and change from baseline at each time point, at least for the primary outcome. P-values may need adjustment for secondary outcomes.

: We have added a new table (Table 2) presenting baseline values, values at each post-baseline visit (Weeks 4, 8, and 12), and the corresponding change from baseline for the primary endpoint (Schirmer's test) in each treatment group. Values for all secondary endpoints (corneal staining, conjunctival staining, TBUT, SPEED, and OSDI) are now provided in S4 Appendix. As this was a Phase 2 exploratory study, no adjustment for multiplicity was pre-specified for secondary endpoints (Protocol Section 11.2.5), and p-values for these outcomes should be interpreted as exploratory (page 14, line 258).

4. What was the block size used for randomization?

: Participants were randomized in a 1:1:1 ratio with variable block size of 6 and 9. It is added to Study protocol subsection (page 9, line 161).

5. Table 1 should assess balance across all three groups, not only treatment vs control. There is no need to label the statistical method in the p-value column; instead, specify the methods in the Statistical Analysis section (e.g., continuous variables were compared using t-tests and categorical variables using chi-square tests).

: We revised Table 1 to compare baseline characteristics across all three treatment groups simultaneously and moved the statistical test labels from the p-value column to the Statistical Analysis section, as suggested.

6. In Figure 1, “Safety” should replace “Saftey.” Replace “Safety Set” with “FAS,” and omit “Excluded from the FAS n=0.” There is no need to show safety set in the flow diagram.

: We have revised Figure 1 accordingly.

7. In addition, the exclusion criteria for the PPS are unclear. For example, is use of a contraindicated drug considered a compliance issue or a protocol deviation/violation also?

We clarify that compliance is defined by dosing adherence rate, and is therefore distinct from contraindicated drug use. The apparent overlap arose because "Protocol deviation/violation" in Figure 1 was missing the word "Other," implying it could include contraindicated drug use as well. We have corrected this to "Other protocol deviation/violation" to clarify that these are separate, non-overlapping exclusion categories.

8. Clarify the reasons for dropouts (e.g., lack of efficacy vs safety-related reasons).

: Of the 8 dropouts, one was attributable to a drug-related adverse event; four were due to voluntary withdrawal of consent; three were due to protocol-related reasons—including use of a prohibited concomitant medication (antihistamine), prior use of cyclosporine eye drops within the screening exclusion window found out after randomization, and incomplete screening laboratory tests—none related to lack of efficacy. This clarification has been added to the S2 Appendix.

Reviewer #2: The manuscript entitled “Comparative study on the clinical effectiveness of low-concentration cyclosporine eye drops in patients with dry eye syndrome” addresses a clinically relevant question regarding whether lower-concentration nanoparticle-based cyclosporine formulations can provide comparable efficacy and tolerability to conventional cyclosporine 0.05% emulsion in dry eye syndrome. The multicenter, randomized, double-blind design is a strength, and the topic is potentially valuable. However, the present manuscript requires several important corrections before it can be considered acceptable. I recommend minor revision, provided that the authors address the following points rigorously.

1. First, the trial-registration timing must be clarified transparently. The manuscript states that recruitment began on 4 January 2023 and that ClinicalTrials.gov registration was first posted on 17 February 2023. This raises concern that the trial may have been registered after participant recruitment had already started. The authors must explicitly state whether registration was prospective or retrospective, whether the protocol was finalized before enrollment, and whether any outcomes were changed after enrollment began.

: This study was conducted as an MFDS-authorized clinical trial in Korea. Prior to enrollment of the first participant, the study protocol, informed consent form, and related study documents were reviewed and approved by both the Ministry of Food and Drug Safety (MFDS) and the Institutional Review Board (IRB). The study objectives, eligibility criteria, endpoints, and statistical analysis plan were finalized before participant enrollment commenced.

Although the ClinicalTrials.gov record was first posted on 17 February 2023, after enrollment had begun on 4 January 2023, registration on ClinicalTrials.gov was not a regulatory requirement for conduct of this trial in Korea. The study was registered to enhance transparency and public accessibility of trial information.

Importantly, no changes were made to the study objectives, primary endpoint, secondary endpoints, eligibility criteria, or statistical analysis plan after participant enrollment began. The timing of the ClinicalTrials.gov posting did not reflect any modification of the study design or outcome measures. This clarification has been added to the Study design section of the revised manuscript (page 8, line 132).

2. Second, the interpretation of efficacy is too favorable relative to the actual primary endpoint. The primary endpoint was change in tear volume at 12 weeks, and although all groups improved from baseline, there were no significant intergroup differences. Therefore, the conclusion should not imply superiority of SCAI-001. The authors should state clearly that SCAI-001 showed comparable efficacy to Restasis, while the earlier 4-week tear-volume improvement was exploratory and not supported by significant between-group difference.

We thank the reviewer for this important observation. We have revised the Abstract and Discussion to state clearly that SCAI-001 demonstrated efficacy comparable to Restasis, and that the earlier tear-volume improvement observed at 4 weeks was a secondary, exploratory finding not supported by a statistically significant between-group difference (page 19, line 360), (page 23, line 452).

3. Third, the statistical analysis requires clearer correction for multiplicity. The manuscript reports many secondary outcomes across multiple time points, including Schirmer’s test, corneal staining, conjunctival staining, TBUT, SPEED, and OSDI at 4, 8, and 12 weeks. Without adjustment for multiple comparisons, isolated findings such as better conjunctival staining with SCAI-001 0.01% at weeks 4 and 8 may be false-positive. The authors should clarify whether multiplicity adjustment was planned or explicitly label these findings exploratory.

As this was an exploratory Phase 2 trial, no multiplicity adjustment was applied across secondary endpoints and time points, per the pre-specified statistical analysis plan (Protocol Section 11.2.5). All secondary endpoint findings should therefore be interpreted as exploratory rather than confirmatory. This clarification has been added to Statistical analysis section of the revised manuscript (page 14, line 256).

4. Fourth, the handling of missing data and attrition requires more detailed justification. Of 116 randomized participants, only 96 completed the study and were included in the per-protocol set. The authors used last observation carried forward for missing efficacy endpoints. This approach can bias treatment-effect estimation in dry eye trials with fluctuating symptoms. The authors should provide reasons for withdrawal by group, compare baseline features of completers and non-completers, and add sensitivity analyses using available-case or mixed-model methods.

: We thank the reviewer for this comment. Reasons for withdrawal, baseline comparisons between completers and non-completers, and a sensitivity analysis using available cases are now provided in S2 Appendix. No significant baseline differences were observed between completers and non-completers, and the sensitivity analysis showed no meaningful differences from the primary analysis (page 14, line 270).

5. Fifth, the conflict-of-interest and funding statements require careful revision. The submission form states that SCAI Therapeutics supported the study and had a role in study design, data collection, and analysis, yet the competing-interest statement says that no competing interests exist. This is not sufficiently transparent. The authors should explicitly disclose sponsor involvement in trial design, product supply, data management, statistical analysis, manuscript preparation, and publication decisions, and clarify whether any authors received payment, consulting support, or institutional funding.

: We thank the reviewer for pointing out this inconsistency between the funding disclosure and the competing-interest statement. It is revised as follows.

- Competing Interests : The authors declare no personal competing interests. No authors received personal payments, or other financial compensation from SCAI Therapeutics in relation to this study.

- Funding statement : SCAI Therapeutics funded the study and provided the investigational product. Research funding was provided to the participating institutions to support study conduct in accordance with the clinical trial agreement. The sponsor participated in study design, data collection oversight, data management, and statistical analysis. However, SCAI Therapeutics did not participate in manuscript drafting or preparation. The authors retained responsibility for data interpretation and the decision to submit the manuscript for publication.

Changes in competing interests is provided in the comment field, as the "Competing Interests" section of the submission form was not editable.

6. Sixth, the manuscript would benefit from more comprehensive contextualization within the current dry eye disease treatment landscape. Several relevant systematic reviews and meta-analyses addressing topical therapy, ocular surface management, and adjunctive dry eye interventions should be considered to strengthen the Introduction and Discussion. The following listed 4 studies are very helpful to consider for citation: 1. PMID: 40679590; DOI: 10.1007/s00210-025-04454-8, because it directly compares topical ophthalmic drugs for keratoconjunctivitis sicca and is highly relevant to positioning cyclosporine among current pharmacologic options; 2. PMID: 40393913; DOI: 10.1016/j.clae.2025.102438, because punctal plugs are a commonly used non-pharmacologic strategy and provide broader context for tear-volume improvement in dry eye disease; 3. PMID: 40352434; DOI: 10.1177/25158414251338775, because thermal pulsation therapy is relevant to meibomian gland dysfunction-associated dry eye and helps contextualize non-immunomodulatory treatment approaches; and 4. PMID: 40204900; DOI: 10.1038/s41433-025-03790-6, because botulinum toxin A represents an emerging dry eye intervention and broadens the discussion of current and developing therapeutic strategies.

: We thank the reviewer for this valuable suggestion. We have added a citation to the Introduction (page 6, line 85).

Overall, this manuscript addresses an important therapeutic question and provides useful randomized clinical data. However, the authors must correct the trial-registration transparency issue, moderate efficacy claims, address multiplicity, clarify attrition and missing-data handling, and improve disclosure of sponsor involvement. After these minor but necessary revisions, the manuscript can be accepted for publication.

Reviewer #3:

1. Missing data on baseline dry eye severity. Please supplement this part of the results. Baseline imbalance could confound results.

: We have added new tables (Table 2 and S4 appendix) presenting baseline values.

2. In this Clinical Trial, the primary efficacy endpoint was the change in tear volume after 12 weeks. While, as we all known, the Schirmer test, with poor reproducibility and high stimulation effect, has a high coefficient of variation, is of limited clinical relevance compared to objective signs like corneal staining and BUT. Please explain the reason for using tear volume as the primary efficacy endpoint.

: We appreciate the reviewer's insightful comment regarding the choice of primary efficacy endpoint. We acknowledge that Schirmer's test is known to have relatively high inter-individual and intra-individual variability.

However, tear volume was selected as the primary endpoint for the following reasons. First, this design followed regulatory and academic precedent set by cyclosporine-class ophthalmic formulations. Restasis® (cyclosporine 0.05%) was approved by FDA in 2003 based on a statistically significant improvement in tear production measured by Schirmer’s test (De Oliveira et al., Clin Ophthalmol 2019). This same endpoint design was subsequently adopted as the sole primary efficacy endpoint in the phase 3 trial of OTX-101 (cyclosporine ophthalmic solution 0.09%, approved by the FDA in 2018 as Cequa®), in which a significantly greater proportion of eyes achieved improvement in Schirmer's test score (Goldberg et al., Ophthalmology 2019).

Second, SCAI-001 was developed with the primary mechanistic goal of enhancing corneal penetration and normalizing sensory nerve signaling to the lacrimal gland, thereby increasing quantitative tear secretion; tear volume change is therefore the endpoint most directly aligned with this proposed mechanism of action.

We have added a statement regarding this point as limitations in discussion section and noting it as a consideration for future trial design (page 22, line 440).

3. As authors has mentioned in the introduction, SCAI eye drops (0.01% and 0.02% cyclosporine) with lower concentrations, aiming to alleviate discomfort such as ocular irritation and foreign body sensation caused by the formulation of conventional cyclosporine eye drop such as 0.05% cyclosporine (Restasis, Allergan Inc.). While, safety outcomes showed that in the ocular tolerability evaluation, no differences were determined in the severity scores of the seven symptoms between the study and control groups. Does this result deviate from the main purpose of the innovative low concentration cyclosporine eye drops?

: We thank the reviewer for this insightful and

Attachment Submitted filename: Response to Reviewers.docx

# Peer review: Decision Letter 1

**DOI:** 10.1371/journal.pone.0358007.r003

**Authors:** Kofi Asiedu

25 Aug 2026

Comparative study on the clinical effectiveness of low-concentration cyclosporine eye drops in patients with dry eye disease

PONE-D-25-46652R1

Dear Dr. Lee,

We’re pleased to inform you that your manuscript has been judged scientifically suitable for publication and will be formally accepted for publication once it meets all outstanding technical requirements.

Within one week, you’ll receive an e-mail detailing the required amendments. When these have been addressed, you’ll receive a formal acceptance letter and your manuscript will be scheduled for publication.

An invoice will be generated when your article is formally accepted. Please note, if your institution has a publishing partnership with PLOS and your article meets the relevant criteria, all or part of your publication costs will be covered. Please make sure your user information is up-to-date by logging into Editorial Manager at Editorial Manager® and clicking the ‘Update My Information' link at the top of the page. For questions related to billing, please contact billing support.

If your institution or institutions have a press office, please notify them about your upcoming paper to help maximize its impact. If they’ll be preparing press materials, please inform our press team as soon as possible -- no later than 48 hours after receiving the formal acceptance. Your manuscript will remain under strict press embargo until 2 pm Eastern Time on the date of publication. For more information, please contact onepress@plos.org.

Kind regards,

Kofi Asiedu, O.D., Ph.D. FAAO

Academic Editor

PLOS One

Additional Editor Comments (optional):

Reviewers' comments:

Reviewer's Responses to Questions

**Comments to the Author**

Reviewer #1: All comments have been addressed

**********

2. Is the manuscript technically sound, and do the data support the conclusions?

Reviewer #1: (No Response)

**********

3. Has the statistical analysis been performed appropriately and rigorously?

Reviewer #1: (No Response)

**********

4. Have the authors made all data underlying the findings in their manuscript fully available?

The PLOS Data policy

Reviewer #1: (No Response)

**********

5. Is the manuscript presented in an intelligible fashion and written in standard English?

Reviewer #1: (No Response)

**********

Reviewer #1: (No Response)

**********

what does this mean?). If published, this will include your full peer review and any attached files.

If you choose “no”, your identity will remain anonymous but your review may still be made public.

**Do you want your identity to be public for this peer review?**  For information about this choice, including consent withdrawal, please see our Privacy Policy

Reviewer #1: No

**********

# Editor report: Acceptance letter

**DOI:** 10.1371/journal.pone.0358007.r004

**Authors:** Kofi Asiedu

PONE-D-25-46652R1

PLOS One

Dear Dr. Lee,

I'm pleased to inform you that your manuscript has been deemed suitable for publication in PLOS One. Congratulations! Your manuscript is now being handed over to our production team.

At this stage, our production department will prepare your paper for publication. This includes ensuring the following:

* All references, tables, and figures are properly cited

* All relevant supporting information is included in the manuscript submission,

* There are no issues that prevent the paper from being properly typeset

You will receive further instructions from the production team, including instructions on how to review your proof when it is ready. Please keep in mind that we are working through a large volume of accepted articles, so please give us a few days to review your paper and let you know the next and final steps.

Lastly, if your institution or institutions have a press office, please let them know about your upcoming paper now to help maximize its impact. If they'll be preparing press materials, please inform our press team within the next 48 hours. Your manuscript will remain under strict press embargo until 2 pm Eastern Time on the date of publication. For more information, please contact onepress@plos.org.

You will receive an invoice from PLOS for your publication fee after your manuscript has reached the completed accept phase. If you receive an email requesting payment before acceptance or for any other service, this may be a phishing scheme. Learn how to identify phishing emails and protect your accounts at https://explore.plos.org/phishing.

If we can help with anything else, please email us at customercare@plos.org.

Thank you for submitting your work to PLOS One and supporting open access.

Kind regards,

PLOS One Editorial Office Staff

on behalf of

Dr. Kofi Asiedu

Academic Editor

PLOS One
